# Intracranial volume: To adjust or not to adjust? It is not a matter of if, but how

**DOI:** 10.1162/IMAG.a.1235

**Published:** 2026-05-22

**Authors:** Aliza Brzezinski-Rittner, Roqaie Moqadam, Yashar Zeighami, Mahsa Dadar

**Affiliations:** Cerebral Imaging Center, Douglas Mental Health University Institute, Québec, Canada; Department of Psychiatry, McGill University, Montréal, QC, Canada; Integrated Program in Neuroscience, McGill University, Montréal, QC, Canada; Faculty of Medicine, University of Montreal, Montréal, QC, Canada; Centre de Recherche, Institut Universitaire de Gériatrie de Montréal, Montréal, QC, Canada

**Keywords:** aging, sex, magnetic resonance imaging, cortical thickness, surface area, volume, deformation-based morphometry, head size, brain size, intracranial volume, correction

## Abstract

Total intracranial volume (TIV) is a major confounding factor in neuroimaging studies, particularly when studying sex differences in the brain. Different methods have been proposed to adjust for this effect; however, their impact has not been directly studied and compared. Furthermore, when studying cortical metrics at the vertex level, the choice of smoothing level can impact analysis outcomes which can in turn impact the degree of TIV-based biases and the effectiveness of the correction methods. In this study, we sought to evaluate the impact of four most commonly used adjustment methods in the literature on the estimations of neuroanatomical sex differences. These methods included the proportions method, the residuals method, the power-corrected proportions method, and adding TIV as a covariate in a regression analysis. Leveraging data from the UK Biobank, we employed a matching approach to devise a gold standard as reference for comparing TIV correction methods. To achieve this, we matched the male and female participants based on age and TIV to remove the impact of TIV differences between sexes. We further modeled aging trajectories at the regional level, vertexwise using data with different smoothing levels, and voxelwise, using raw and adjusted values, and compared the obtained estimates against the gold standard. We found that across different metrics, adding TIV as a covariate was the best-performing method for removing the effect of TIV, in terms of the correlation between the estimates of the different subsamples and the gold standard as well as the degree of estimation bias. Furthermore, we showed that the commonly used smoothing of the morphometric measures can result in biased estimation of sex differences in these measures. Finally, we showed that while small in effect size, there still remains some neuroanatomically specific uncorrected effects for all adjustment methods.

## 1 Introduction

Magnetic resonance imaging (MRI) is one of the most commonly used tools for studying structural brain changes, as well as diagnosis and monitoring of certain pathologies. Regional brain volumes have been used in numerous studies, comparing healthy individuals and diseased populations, advancing our understanding of the mechanisms involved in and progression of neurodegenerative disorders. For example, volumetric studies consistently report smaller hippocampal volumes in patients with Alzheimer’s disease (Amaral et al., 2018; Planche et al., 2022; Sabuncu et al., 2011). Another commonly used metric to assess brain changes is cortical thickness (CT), which measures the depth of the cortical layers as the distance between the white matter and gray matter boundaries and the brain’s pial surface (Lerch, 2015). For example, Parkinson’s disease patients reportedly experience greater levels of cortical thinning than matched healthy controls (Yau et al., 2018). Similarly, cortical surface area (SA) can be used as a measure of “cortical column generation” (Rakic, 2009). Other commonly used metrics to study brain atrophy are deformation-based morphometry (DBM), which captures local atrophy at the voxel level by estimating the deformation a preprocessed image goes through in a non-linear registration to an average template (Ashburner et al., 1998), and voxel-based morphometry (VBM), which derives tissue probability maps modulated by DBM as an estimation of density for different tissue types. For example, frontotemporal dementia patients experience widespread atrophy as measured by DBM in frontal and temporal brain regions (Manera et al., 2019; Metz et al., 2025). Regional volumetric measures in stereotaxic space can be further estimated based on these voxelwise measures. Interestingly, these metrics (volumes, CT, SA, DBM, and VBM) can scale differently in relation to brain size as measured by total intracranial volume (TIV) (Im et al., 2008).

Imaging-derived findings based on these metrics are inevitably impacted by a series of methodological and processing choices. Of particular interest are the choices made to account for the effect of intracranial volume size differences when performing population level analyses such as investigating group differences in case–control studies. For example, it has been shown that brain size impacts brain asymmetry findings, and that appropriate adjustment of this effect reveals specific age and sex effects on brain asymmetry (C. M. Williams et al., 2022). Another methodological choice pertains to smoothing (at vertex or voxel level), which is commonly used to improve registration and inter-subject alignment, reduce noise, and increase statistical power (Lerch & Evans, 2005; Lerch et al., 2006; Worsley et al., 1999; Zeighami & Evans, 2021; Zhao et al., 2013). However, when it comes to regional analyses, the smoothing level and resolution of the selected parcelation might have a differential effect on the results (Zeighami & Evans, 2021). It remains unclear how the selection of a given degree of smoothing and the method used to account for the impact of TIV in brain morphometry studies might interact.

It has been shown that many of the reported sex differences in the brain are driven by intracranial volume, which is on average 12% larger in males than in females (Eliot et al., 2021; Ruigrok et al., 2014). While most cortical and subcortical brain regions appear to have significantly larger volumes in males than in females, females present with greater overall cortical thickness, and the effect of these differences decrease, or reveal different patterns when the analyses appropriately account for intracranial volume (Brzezinski-Rittner et al., 2025; Ritchie et al., 2018). A common metric used for adjustments (also referred to as normalization) is the TIV, as it is assumed to be constant across the individual’s lifespan and is largely unaffected by aging or pathological processes (Farias et al., 2012; Hentschel & Kruggel, 2004; O’Brien et al., 2006). Furthermore, when it comes to cortical measures such as cortical volume (CV), CT, or SA, researchers have used specific “global metrics” to adjust statistical models for these values, namely total cortical volume, mean CT, or total SA (C. M. Williams et al., 2021). However, the impact of these choices has also not been systematically studied.

There have been multiple proposed methods for adjusting for TIV, as well as attempts to evaluate and compare these methods (Greenberg et al., 2008; Nordenskjöld et al., 2013, 2013; O’Brien et al., 2006, 2011; Pell et al., 2008; Pintzka et al., 2015; Sanchis-Segura et al., 2019, 2020; Sanfilipo et al., 2004; van Eijk et al., 2020; Voevodskaya et al., 2014). Most studies have focused on a small number of brain structures, instead of taking a brainwide approach. Furthermore, the vast majority of these studies centered exclusively on volumetric or VBM data, and few have used an approach that permits comparisons with a gold standard (i.e., matching). More recently, studies have utilized correction approaches to account for the confounding effect of brain size on sex differences, with a focus on accurate classification of females vs males based on structural MRI measures (beyond brain size) (Sanchis-Segura et al., 2020, 2022), and in relation to cognition (Dhamala et al., 2022; J. Wang et al., 2024). While employing correction methods removes (some) of the TIV-related differences, each method might also introduce different types of bias when assessing the potential presence of sex differences in normalized values, and in the absence of a gold standard approach, the results remain inconclusive.

Brain size correction methods have been mainly studied in the context of regional brain volumes. Among these, one of the first to be proposed was the *proportions* method, which consists of dividing any given volume of interest (VOI) by TIV, and assumes a proportional relationship between any VOI and TIV, which contradicts the allometry principle in brain development (de Jong et al., 2017; Deacon, 1988; Huxley & Teissier, 1936; Reardon et al., 2018). The *residuals* method has been implemented using different formulations through the literature and has been frequently evaluated (Arndt et al., 1991; Mathalon et al., 1993; Sanfilipo et al., 2004), but overall, it involves “performing a linear regression of each of the raw measures on head size within the normal control group and then using the residuals from this ‘control regression line’ as the head-size-corrected unit of analysis for each participant (i.e., control participant and patients).” (Mathalon et al., 1993), thus expressing the adjusted VOI as a deviation from the predicted value in a healthy participant with a given TIV. In the case of comparing two groups, as it can be the study of patients versus controls, the underlying assumption for calculating the slope only in the control group is that, in that case, the slope represents a “normal” relationship between any given VOI and TIV, which might not be the case in the disease group (Voevodskaya et al., 2014). When looking at sex differences, it has been shown that if each sex is normalized separately, the normalized values for males are larger than the normalized values in females, due to the baseline difference in TIV and raw values (Nordenskjöld et al., 2015). The same study by Nordenskjöld et al. (2015) concluded that “selecting the residual method using one group to normalize the cohort is motivated when studying differences between healthy and diagnosed subjects, but not when studying gender differences” (Nordenskjöld et al., 2015). An equation commonly used is *VOI_adj_ = VOI – b(TIV – TIV_mean_)*, where *b* is the slope of the regression line between *VOI* and *TIV* (Jack et al., 1989; Nordenskjöld et al., 2015; Sanchis-Segura et al., 2019, 2020; Voevodskaya et al., 2014), and *TIV_mean_* is the mean of the TIV of all the participants included in the regression, which serves to center the adjusted values around the expected TIV value. The *power-corrected-proportions* (PCP) method (Liu et al., 2014) was developed with the idea that the relationship between several VOIs and TIV follows the power law principle (Im et al., 2008; Lüders et al., 2002), instead of being either proportional or linear. According to this principle, VOI and TIV are related through $VOI\;=\;\alpha ICV^{\beta }$, and one can use an estimation of β to account for this power relationship when using the proportion of VOI to TIV. Lastly, a common practice in neuroimaging studies is adding TIV as a covariate in statistical models, or regressing it out with a simple linear regression before performing any additional analyses (Griffiths-King et al., 2024; Kotikalapudi et al., 2023; Raykov et al., 2025; Wen et al., 2025; M. E. Williams et al., 2025; Wuestefeld et al., 2025; Young et al., 2018), which similar to the regression method, while accounting for regional differences, is based on the same assumption of linearity.

In this work, we aim to (a) evaluate the effectiveness of different brain size adjustment methods in removing the effect of brain size on sex differences in aging trajectories using different structural imaging metrics, (b) identify the remaining bias after applying different correction methods, and (c) assess the effect of smoothing levels on derived sex differences for different adjustment methods. To address these questions, we used a matching approach similar to the one used previously by Brzezinski-Rittner et al. (2025); Kurth et al. (2018); Luders et al. (2009, 2014); Pintzka et al. (2015); Sanchis-Segura et al. (2019); Sowell et al. (2007); Voevodskaya et al. (2014); L. Wang et al. (2012), taking advantage of the large sample size provided by the UK Biobank (UKBB) dataset.

## 2 Methods

### 2.1 Datasets

Data included participants from the UK Biobank, an open-access large prospective study with phenotypic, genotypic, and neuroimaging data from 500,000 individuals recruited between 2006 and 2010 at 40–69 years old in Great Britain (Bycroft et al., 2018; Littlejohns et al., 2020; Sudlow et al., 2015). All participants provided informed consent (“Resources tab” at https://biobank.ctsu.ox.ac.uk/crystal/field.cgi?id=200). The UK Biobank received ethical approval from the Research Ethics Committee (reference 11/NW/0382), and the present study was conducted based on application 45551. Sex was assigned according to the “Sex” variable (data field 31). Brain imaging data were acquired from a subset of participants.

### 2.2 Image processing

Data included in this study were obtained from the first imaging visit (instance 2). We extracted regional and vertexwise volume, cortical thickness, and surface area information by processing the raw T1-weighted images using FreeSurfer v.7.4.1.(Fischl, 2012), via the recon-all command. For regional information, we used the Desikan–Killiany parcellation (Desikan et al., 2006) and aseg segmentations for the cortical and subcortical regions, respectively. For vertexwise data, we extracted the values for each of the three metrics, registered to the standard FsAverage template, and smoothed with all available levels (i.e., 0, 5, 10, 15, 20, and 25 mm at full-width half-maximum), without any further modification. Furthermore, we used PELICAN (Dadar et al., 2025), an extensively validated and widely used (Kamal et al., 2025; Lajoie et al., 2025; Metz et al., 2025; Moqadam et al., 2025; Qiu et al., 2024) in-house pipeline based on the open source MINC and ANTs tools to obtain accurate TIV estimations as well as regional and voxelwise DBM values to capture local atrophy (tissue shrinkage) or expansions (Dadar et al., 2018, 2020, 2021, 2022; Manera et al., 2021; Misquitta et al., 2020; Zeighami et al., 2019). We used PELICAN-derived TIV values as it has been previously shown that the estimated TIV (eTIV) from Freesurfer is biased and might not be optimal for TIV normalization (Crowley et al., 2018; Klasson et al., 2018; Nordenskjöld et al., 2013). The preprocessing procedure consisted of denoising based on optimized non-local means filtering (Coupe et al., 2008), correction for intensity inhomogeneity (Sled et al., 1998), and intensity normalization using linear histogram matching. The resulting images were linearly registered to the MNI152-2009c template (nine parameters) via a hierarchical linear registration method (Dadar et al., 2018). Scaling factors derived from the linear transformations (reflecting the TIV difference between the participant and MNI152-2009c template) were used to estimate TIVs for each participant (Dadar et al., 2025). Following linear registration, each scan was non-linearly registered to the MNI152-2009c template. We obtained voxelwise DBM maps by using the non-linear transformations to calculate the Jacobian determinant of each participant’s deformation matrix at each voxel. Finally, we also calculated individual regional volumes based on the CerebrA atlas, whose regions are equivalent to the DKT atlas used by FreeSurfer, and further includes subcortical structures (Manera et al., 2020).

Linear and non-linear registrations were visually quality controlled (QC) by two experienced raters (Y.Z. and M.D.), using a procedure previously described in Dadar et al. (2018). In summary, for the linear registration, QC images were generated by overlaying the contours of the MNI152-2009c template on the registered images. The raters assessed the alignment of the contours with the registered image on sagittal, coronal, and axial views. To assess the alignment, the outline of the brain, central sulcus, cingulate sulcus, and parieto-occipital fissure was used as anatomical landmarks. Ensuring the accuracy of the linear registration process guarantees the accuracy of the scaling factors used for TIV estimation.

### 2.3 Matching process

Exclusion criteria included: missing demographic information (e.g., age, sex), failed visual quality control of image processing and registration steps, and failure in FreeSurfer’s reconstruction. After these evaluations, our final sample included 35,732 participants (54% females). From this sample, we created four subsamples, which included an equal number of female and male participants, using the same method described in our previous work (Brzezinski-Rittner et al., 2025). Briefly, the first subset is referred to as the **matched sample**, and was matched by TIV and age, calculated as months between the participant’s month and year of birth (data fields 52 and 34), and the date of visiting the assessment center (data field 53) for the imaging visit (instance 2). The matching process consisted of first splitting the whole sample by sex, randomizing the order within each group, and using an iterative process to find a female whose age in months and estimated TIV were within 0.02% of the corresponding values for a given male. We chose to find females to match to their male counterparts since the female group was larger than the male group (19,281 F vs 16,451 M). This process yielded a sample of 11,294 matched participants (5,647 per sex). We created a second subsample with the same size (11,294 participants) matched only by age, referred to as the **age-matched sample**. The third sample we created, referred to as **not-matched sample**, had the same size; however, it mimicked the age and TIV distribution of the original full sample, to allow for comparisons of the results across matching strategies with the same statistical power, and allowing us to investigate the results in a sample characteristic of the complete sample while matching the sample size of the other subsamples. The next sample is referred to as **extreme sample**, in which we exaggerated the TIV difference between females and males by excluding all the participants who were included in the matched sample and keeping 5,647 participants per sex whose age distribution was similar to the original sample. Table 1 summarizes the information for all generated samples.

**Table 1.** Description of the characteristics of the subsamples included in this study.

Sex	N	Age range	Age (mean, sd)	TIV (mean, sd)
**Full dataset**				
Female	19,281 (54%)	45 - 81 years	62.8, (7.36)	1,336,544.0, (103,196.89)
Male	16,451 (46%)	44 - 82 years	64.0, (7.64)	1,525,386.9, (119,264.92)
**TIV and age-matched sample**				
Female	5,647 (50%)	47 - 80 years	63.3, (7.06)	1,426,776.1, (83,886.29)
Male	5,647 (50%)	47 - 80 years	63.3, (7.06)	1,426,823.3, (83,880.34)
**Age-matched sample**				
Female	5,647 (50%)	45 - 81 years	64.0, (7.67)	1,336,051.7, (103,836.41)
Male	5,647 (50%)	45 - 81 years	64.0, (7.67)	1,525,577.3, (117,849.54)
**Not-matched sample**				
Female	5,647 (50%)	46 - 81 years	62.8, (7.39)	1,338,532.7, (103,282.83)
Male	5,647 (50%)	44 - 81 years	64.0, (7.67)	1,524,394.1, (118,165.63)
**Extreme sample**				
Female	5,647 (50%)	45 - 81 years	63.6, (7.02)	1,297,634.6, (85,362.83)
Male	5,647 (50%)	44 - 82 years	64.4, (7.95)	1,574,611.6, (101,046.49)

TIV: total intracranial volume, sd: standard deviation.

### 2.4 Modeling

The modeling strategies described below were used for our eight metrics of interest: vertexwise cortical volume, cortical thickness, and surface area, voxelwise DBM, regional volume, cortical thickness, and surface area, and regional volumes derived from DBM values. Each model was evaluated for each of the four subsamples.

We modeled the aging trajectory of each metric using the following linear regression model (Eq. 1), accounting for age, sex, and their interaction:

MOI ~ 1 + age + sex + age:sex, (1)

where *MOI* denotes the measure of interest, *age* is the age at MRI acquisition time in months, and *sex* is the participants’ sex. In all cases, all the numerical variables were z-scored.

To assess whether commonly used adjustment methods can account for TIV differences with regard to accurately estimating sex-differentiated aging trajectories, we repeated the same aging trajectory analyses (Eq. 1) using adjusted values instead of the raw values. An ideal correction method that has appropriately captured the effects of TIV differences would yield similar sex-differentiated aging trajectories to those obtained based on the matched sample. We examined the following correction methods:

1. Proportions method: for each participant, the regional volume is divided by their TIV, using the following equation:
  $$MOI_{adj}\;=\;MOI/TIV.$$ (2)
  This method has the underlying assumption that all brain VOIs are proportional to TIV.
2. Power-corrected proportions method (PCP) (Liu et al., 2014): the regional volume is adjusted via:
  $$MOI_{adj}\;=\;MOI/TIV^{b},$$ (3)
  where *b* denotes the slope (regression coefficient) associated with TIV in the model
  log(MOI)∼1+log(TIV).
3. Residuals method (Arndt et al., 1991; Jack et al., 1989; Mathalon et al., 1993): where the *MOI* is adjusted via the following equation:
  $$MOI_{adj}\;=\;MOI-b\left(TIV-\bar{TIV}\right),$$ (4)
  where *b* denotes the slope associated with TIV in the model *VOI∼1+TIV*, and $\bar{TIV}$ is the mean TIV of the whole sample (following the formulation by Jack et al. (1989), and recommendations by Sanchis-Segura et al. (2019)).
4. TIV as a covariate (or covariate method): we added TIV as a covariate in the regression model, assuming a linear relationship between MOI and TIV:
  MOI ~ 1 + age + sex + age:sex + TIV. (5)

For all of the regression models, the outcome variable was scaled (z-scored) after the correction method was implemented. We repeated this procedure in each of the subsamples and compared the model estimates against those obtained based on the raw values using the matched sample, by calculating the correlation between the model estimates obtained in the gold standard and those obtained using different adjustment methods in the not-matched sample, as well as the slope of the linear regression between those same estimates, to assess the degree of bias in the estimate’s distribution. Further, we computed paired t-tests and Cohen’s d tests to determine the differences between the standardized estimates obtained using adjusted metrics in the not-matched sample, and the estimates obtained in the matched sample. The p-values associated with the t-tests were corrected using the false discovery rate (FDR) correction. Finally, we obtained the adjusted R^2^ value for all the regression models using TIV as the adjustment metric, and compared them within each sample.

Furthermore, we examined the impact of different smoothing levels (0, 5, 10, 15, 20, and 25 mm at FWHM) at the vertex level, using vertexwise FreeSurfer outcomes for cortical volumes, CT, and SA. For voxelwise DBM data, we performed the analyses per tissue type. For this, we classified the tissue types based on BISON (Dadar & Collins, 2021) segmentations of the MNI152-2009c template.

Finally, we repeated the analyses replacing TIV with each measure’s “global measure” for adjustment. In other words, we used total cortical volume (TCV) as the adjustment measure for the vertexwise cortical volume analyses, total surface area (TSA) for the vertexwise SA, and mean cortical thickness (MCT) for the vertexwise CT, as employed in some studies using surface-based measurements (Ritchie et al., 2018; Westman et al., 2013; Zhou et al., 2014). Regional SA and CT were also adjusted using TSA and MCT.

### 2.5 Bias estimation

We estimated a bias term for each method and metric, representing the residual bias left for the sex estimation after the corrections were performed for each measure. To achieve this, we compared the local (i.e., regional/vertexwise/voxelwise) sex estimates based on the gold standard matched sample against the corrected estimate in the non-matched sample. In an ideal case, the correction results in an identical estimation to that of the gold standard. We calculated the local estimate differences from this ideal scenario (i.e., the orthogonal distance of each region from the reference line) as the remaining bias in the corrected estimation for each adjustment method. The results were then projected on the brain and visualized to examine whether there is any systematic anatomical pattern to this remaining uncorrected bias. Finally, for the regional volumetric data, we evaluated the correlation between the estimated bias and the regional volume allometry maps presented in our prior work (Brzezinski-Rittner et al., 2025) for males and females in the matched and not-matched samples. For this, we performed a spatial permutation (“spin”) test with 10,000 permutations to ensure the results were not a consequence of the intrinsic spatial autocorrelation between brain regions (Alexander-Bloch et al., 2018). For each rotation, we computed the correlation between maps to generate a null distribution, and calculated the empirical p-value as the proportion of permuted correlations exceeding the observed correlation.

### 2.6 Additional validations

In addition, we assessed whether our matched subsample was representative of the full sample by performing the following comparisons between our matched samples and the remaining unmatched participants: (i) chi-squared tests to compare the prevalence of hypertension, diabetes, cardiovascular disease, and dyslipidemias, based on the UKBB category 1712 (“first occurrences”, which contains data showing the “first occurrence” of any code mapped to 3-character ICD-10), (ii) t-tests to compare physical features such as height (data field 12144) and body mass index (BMI; data field 21001), and (iii) chi-squared tests to compare socioeconomic (data field 738, “Average total household income before tax”) and educational variables (via a composite variable considering UKBB data fields 6138 (“Qualifications”) and 845 (“Age completed full time education”).

As a last validation, since the UKBB is an epidemiological dataset, we evaluated the presence of neurodegenerative disorders (i.e., dementias, and Parkinson’s disease or Secondary Parkinsonism) in our sample, based on data field 1712. We assessed the number of participants in the whole sample that presented those diagnoses and further used chi-squared tests to evaluate their prevalence in the matched sample in comparison with the rest of the sample. Finally, we used the same modeling approach for the four samples and five adjustment methods on regional volumetric data after excluding participants who presented a dementia, Parkinson’s disease, or Secondary Parkinsonism diagnosis, and compared the obtained estimates without removing those participants.

Furthermore, we compared the TIV estimates from FreeSurfer versus the TIV computed by PELICAN and replicated the regional analyses in the same subsamples but utilizing the TIV estimations obtained from FreeSurfer (thereafter referred to as FS-TIV) instead of the TIV values extracted with PELICAN. We evaluated the degree in which the estimates obtained with the two TIV values differed from the estimates obtained for the gold standard.

All statistical analyses were performed using the R statistical language and Python.

## 3 Results

### 3.1 Regional data

#### 3.1.1 Regional FreeSurfer volumes

**Fig. 1.**
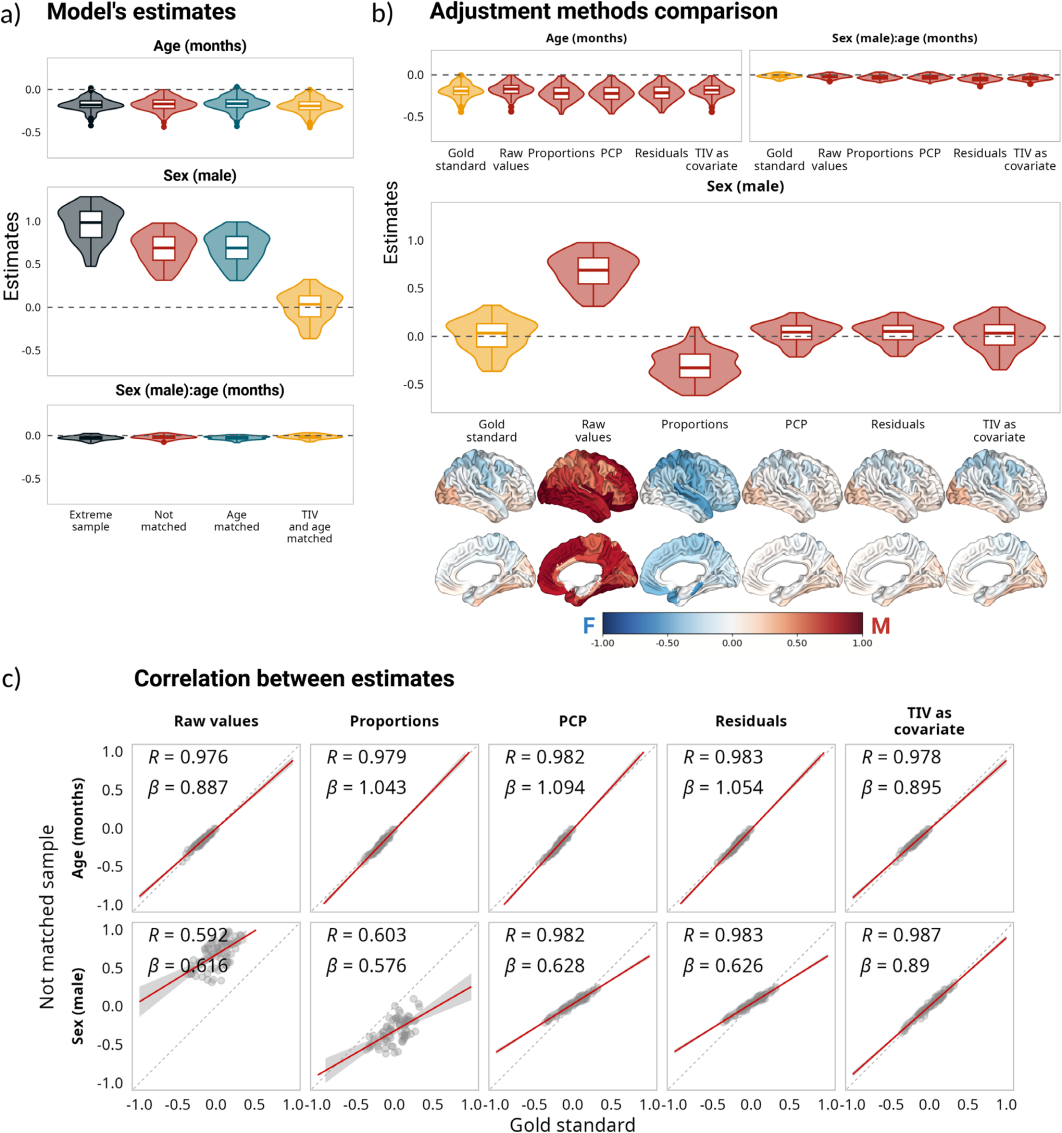
Impact of correction methods on model estimations based on regional volumes. (a) Distribution of estimates for all samples, based on the following model vol ~ 1 + age + sex + age:sex. (b) Comparison of estimates for the matched sample without adjustment and the estimates after using different adjustment methods for the not-matched sample. Below the sex estimates are the corresponding cortical projections of the same regional values. (c) Correlations and linear regression slopes between estimates for the matched sample without adjustment and the estimates after using different adjustment methods for the not-matched sample. Note that the estimates for the interaction term (panels a and b) are small in comparison with the main effects of age and sex. For a comparison of all the estimates, see Supplementary Figure 1.

Figure 1a shows the distribution of the standardized model estimates across the 78 brain regions (62 cortical regions from the DKT atlas and 16 subcortical regions from aseg segmentation) for each sample. While age estimations were consistent across the four samples, sex estimations changed between samples due to the TIV differences between females and males. For instance, both non-matched and age-matched samples had positive sex estimates (with the female group used consistently as reference), indicating that regional volumes were larger for males. The estimations for the extreme sample followed a similar distribution, but were even larger. Meanwhile, in the matched sample, the sex estimates were centered around zero, indicating that although there were some sex differences in the trajectories, they were more subtle and, importantly, bidirectional (i.e., positive for some regions and negative for others). See Supplementary Table 1 for details on the estimates distributions.

Figure 1b compares the distribution of the standardized model estimates between the matched and unmatched samples for different correction methods across the same regions and their projection on the brain surface. For the age estimations, while applying correction methods increased their variability, the adjustments had little effect on them (Supplementary Fig. 1). For the sex estimates, the proportions method reversed the trend of the not-matched sample, and was the only method that significantly changed and reversed the intercept estimations. PCP and residuals methods yielded virtually identical results, both showing the same trend as the gold standard with regard to the sex estimate, but changing the distribution and decreasing the range of the estimations. Interestingly, this decrease in the estimate’s range was stronger for the extreme sample, with a systematic bias underestimating larger effect sizes in both directions.

Figure 1c shows the correlation and the slope of the regression line between the model estimates using different adjustment methods in the not-matched sample and the gold standard. The correlation values reflect the strength of the linear association between the two sets of estimates (i.e., how closely they fit), while the regression slope can indicate systematic over or under estimation; therefore, an ideal correction method would yield both a high correlation and have a slope value close to 1. Adding TIV as a covariate consistently achieved the best results across methods. Residuals and PCP methods also showed high correlations with the gold standard (>0.98 for intercept, age, and sex estimations; see Table 2 for correlation and slope values for the sex estimates). Finally, the model estimates yielded the closest results to the gold standard when TIV was added as a covariate, with an overall distribution that was similar to the estimates of the matched sample without any major bias. For the sex estimate, there was no statistically significant difference between the estimates obtained in the matched sample versus the estimates obtained when we added TIV as a covariate (*t*(77) = -0.64; *p_adj_* = 0.52), and the effect size was negligible (*d* = -0.073). Meanwhile, the effect sizes for the PCP and residuals methods were small (*d* = -0.4, *d* = -0.45 respectively). In the case of age, the effect size of the difference between the estimates in the gold standard and in the not-matched sample without adjustment was large (*d* = -0.9), despite the values having a high correlation and low degree of bias; the smallest difference was between the gold standard and adding TIV as a covariate (*d* = -0.4). For the full set of comparisons for all estimates and all samples, see Supplementary Table 1. To further compare the methods, we evaluated the model’s goodness of fit within the not-matched sample across all the regions through the adjusted R^2^. The mean adjusted R^2^ was higher for adding TIV as a covariate (mean adjusted R^2^ = 0.32) than for any of the other methods, including not using any correction (mean adjusted R^2^ = 0.15; see Table 3 for the mean adjusted R^2^ values for all the methods). The R^2^ difference between not using any correction and adding TIV as a covariate was statistically significant and had a large effect size (*t*(77) = -22.62, *p_adj_* < 0.001; *d* = -2.56). See Supplementary Table 2 for details on adjusted R^2^ values and their comparison.

**Table 2.** Summary of correlations (r) and slopes (b) between the standardized estimates for age and sex obtained in the not-matched sample, before and after adjustment, with the standardized estimates for sex obtained in the matched sample without adjustment.

Level	Metric		Raw values	Proportions	Power-corrected proportions	Residuals	TIV as a covariate
Regional	Volume		r: 0.59 (b: 0.62)	r: 0.6 (b: 0.58)	r: 0.98 (b: 0.63)	r: 0.98 (b: 0.63)	**r: 0.99 (b: 0.89)**
	Surface area		r: 0.46 (b: 0.63)	r: 0.45 (b: 0.61)	r: 0.98 (b: 0.66)	r: 0.98 (b: 0.65)	**r: 0.97 (b: 0.9)**
	Cortical thickness		r: 0.72 (b: 0.53)	r: 0.55 (b: 0.2)	r: 0.99 (b: 0.54)	r: 0.99 (b: 0.54)	**r: 0.99 (b: 0.96)**
	DBM		r: 0.51 (b: 0.53)	r: 0.29 (b: 0.26)	r: 0.99 (b: 0.63)	r: 0.99 (b: 0.63)	**r: 0.99 (b: 1.03)**
Vertex	Smoothing: 0 mm FWHM	Volume	r: 0.7 (b: 0.71)	r: 0.69 (b: 0.74)	r: 0.89 (b: 0.56)	r: 0.93 (b: 0.56)	**r: 0.93 (b: 0.94)**
		Surface area	r: 0.61 (b: 0.7)	r: 0.57 (b: 0.71)	r: 0.83 (b: 0.53)	r: 0.88 (b: 0.56)	**r: 0.88 (b: 0.92)**
		Cortical thickness	r: 0.74 (b: 0.73)	r: 0.55 (b: 0.54)	r: 0.95 (b: 0.54)	r: 0.95 (b: 0.55)	**r: 0.96 (b: 0.96)**
	Smoothing: 20 mm FWHM	Volume	r: 0.82 (b: 0.8)	r: 0.63 (b: 0.57)	r: 0.99 (b: 0.61)	r: 0.99 (b: 0.61)	**r: 0.99 (b: 0.92)**
		Surface area	r: 0.65 (b: 0.71)	r: 0.48 (b: 0.62)	r: 0.98 (b: 0.64)	r: 0.98 (b: 0.64)	**r: 0.98 (b: 0.9)**
		Cortical thickness	r: 0.75 (b: 0.63)	r: 0.59 (b: 0.21)	r: 0.99 (b: 0.55)	r: 0.99 (b: 0.56)	**r: 0.99 (b: 0.97)**
Voxel	DBM: Cortical gray matter		r: 0.73 (b: 0.77)	r: 0.48 (b: 0.63)	r: 0.96 (b: 0.59)	r: 0.97 (b: 0.58)	**r: 0.97 (b: 1)**
	DBM: Deep gray matter		r: 0.49 (b: 0.44)	r: 0.43 (b: 0.3)	r: 0.98 (b: 0.6)	r: 0.98 (b: 0.6)	**r: 0.99 (b: 0.98)**
	DBM: White matter		r: 0.51 (b: 0.55)	r: 0.45 (b: 0.73)	r: 0.97 (b: 0.6)	r: 0.97 (b: 0.6)	**r: 0.97 (b: 1.01)**

In bold, the values that indicate the best adjustment.

**Table 3.** Fit measures. Mean, standard deviation, and range across regions, vertices, or voxels, of the adjusted R^2^ values for each analyzed measure in the not-matched sample, for each adjustment method.

Level	Metric		Raw values	Proportions	Power-corrected proportions	Residuals	TIV as a covariate
Regional	Volume		0.153 (0.066), [0.029, 0.276]	0.104 (0.061), [0.011, 0.287]	0.07 (0.052), [0.003, 0.224]	0.071 (0.052), [0.003, 0.227]	**0.322 (0.111), [0.083, 0.511]**
	Surface area		0.15 (0.055), [0.047, 0.253]	0.054 (0.029), [0.002, 0.131]	0.025 (0.017), [0.003, 0.072]	0.025 (0.017), [0.003, 0.074]	**0.346 (0.116), [0.099, 0.536]**
	Cortical thickness		0.054 (0.038), [0.003, 0.148]	**0.339 (0.047), [0.237, 0.422]**	0.05 (0.036), [0.002, 0.134]	0.05 (0.036), [0.002, 0.134]	0.059 (0.036), [0.014, 0.147]
	DBM		0.038 (0.049), [0.001, 0.285]	**0.211 (0.074), [0.081, 0.404]**	0.03 (0.037), [0, 0.2]	0.029 (0.036), [0, 0.198]	0.059 (0.076), [0.001, 0.386]
Vertex	Smoothing: 0 mm FWHM	Volume	0.005 (0.003), [0, 0.043]	0.005 (0.003), [0, 0.043]	0.003 (0.002), [0, 0.033]	0.003 (0.002), [0, 0.034]	**0.019 (0.009), [0, 0.078]**
		Surface area	0.012 (0.006), [0, 0.041]	0.003 (0.002), [0, 0.024]	0.001 (0.001), [0, 0.012]	0.001 (0.001), [0, 0.012]	**0.027 (0.01), [0, 0.089]**
		Cortical thickness	0.008 (0.007), [0, 0.104]	**0.077 (0.02), [0.002, 0.178]**	0.006 (0.006), [0, 0.095]	0.006 (0.006), [0, 0.095]	0.009 (0.008), [0, 0.103]
	Smoothing: 20 mm FWHM	Volume	0.108 (0.051), [0.008, 0.258]	0.054 (0.023), [0, 0.156]	0.041 (0.022), [0, 0.14]	0.042 (0.022), [0, 0.14]	**0.242 (0.08), [0.05, 0.525]**
		Surface area	0.118 (0.045), [0.022, 0.249]	0.03 (0.019), [0, 0.12]	0.017 (0.014), [0, 0.076]	0.018 (0.014), [0, 0.077]	**0.274 (0.084), [0.062, 0.561]**
		Cortical thickness	0.046 (0.026), [0, 0.139]	**0.207 (0.026), [0.09, 0.287]**	0.041 (0.023), [0, 0.136]	0.041 (0.023), [0, 0.136]	0.051 (0.025), [0, 0.142]
Voxel	DBM: Cortical gray matter		0.007 (0.01), [0, 0.246]	**0.036 (0.026), [0, 0.282]**	0.005 (0.007), [0, 0.222]	0.005 (0.007), [0, 0.219]	0.009 (0.012), [0, 0.266]
	DBM: Deep gray matter		0.067 (0.054), [0, 0.337]	**0.156 (0.05), [0.015, 0.353]**	0.049 (0.044), [0, 0.298]	0.049 (0.044), [0, 0.297]	0.094 (0.066), [0, 0.365]
	DBM: White matter		0.018 (0.028), [0, 0.346]	**0.073 (0.047), [0, 0.357]**	0.015 (0.026), [0, 0.308]	0.015 (0.026), [0, 0.307]	0.022 (0.034), [0, 0.375]

In bold, the method that yields the higher average R^2^.

#### 3.1.2 DBM volumes

**Fig. 2.**
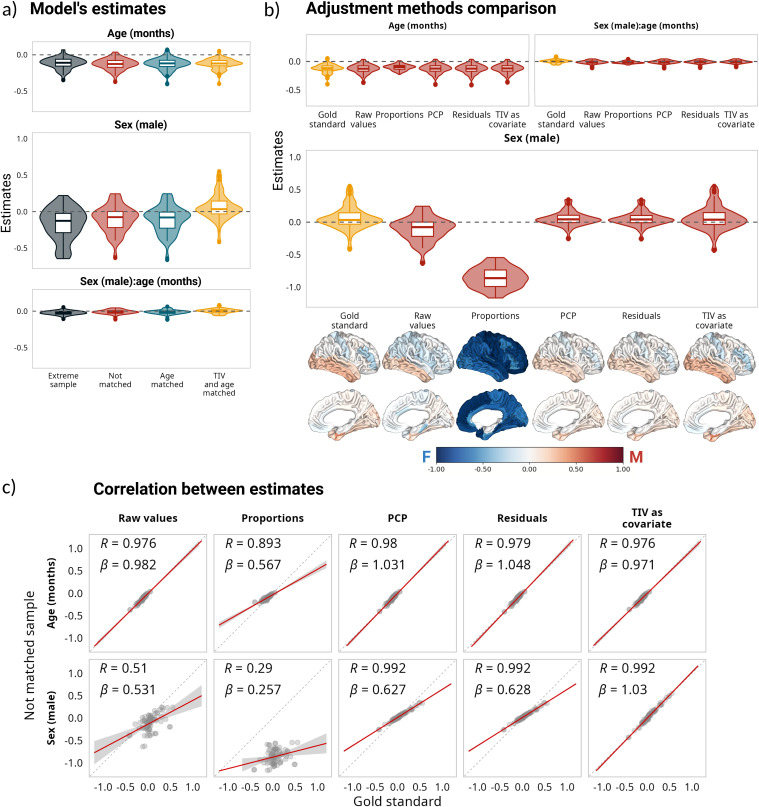
Impact of correction methods on model estimations based on deformation-based morphometry (DBM) measures. (a) Distribution of estimates for all samples, based on the following model DBM ~ 1 + age + sex + age:sex. (b) Comparison of estimates for the matched sample without adjustment and the estimates after using different adjustment methods for the not-matched sample. Below the sex estimates are the corresponding cortical projections of the same values. (c) Correlations and linear regression slopes between estimates for the matched sample without adjustment and the estimates after using different adjustment methods for the not-matched sample. Note that the estimates for the interaction term (panels a and b) are small in comparison with the main effects of age and sex. For a comparison of all the estimates, see Supplementary Figure 3.

Similar to the previous section, Figure 2a shows the distribution of the standardized model estimates across the 78 brain regions for each sample. The age estimates remained constant across the four samples; however, compared with the volumes extracted in the participants’ native space, both the intercepts and the sex estimates were smaller in magnitude and had the opposite direction for the extreme, the non-matched, and the age-matched samples. This behavior is potentially explained by DBM values already being normalized to a reference template (i.e., MNI-ICBM152) via a linear registration prior to estimating the non-linear transformation. The sex estimates for the matched sample (i.e., gold standard method) were centered around zero and the different adjustment methods (Fig. 2b) yielded results equivalent to those obtained with the regional volumes (Fig. 1b), with the residuals and PCP methods showing high correlations with the gold standard (⍴ = 0.98 for the intercept, age, and sex estimations; Fig. 2c). Additionally, from the different correction methods, only the proportions method had a distinguishable effect on the age estimations. Once again, the model estimates yielded the closest results to the gold standard when TIV was added as a covariate, in terms of correlation and bias. For the sex estimate, there was no statistically significant difference between the estimates obtained in the matched sample versus the estimates obtained when we added TIV as a covariate (*t*(77) = 0.31; *p_adj_* = 1), and the effect size of the difference was negligible (*d* = 0.036). The effect sizes for the PCP and residuals methods were also negligible (*d* = 0.1, *d* = 0.13, respectively), and the difference was not statistically significant either. In the case of age, the effect size of the difference between the estimates in the gold standard and in the not-matched sample without adjustment was small (*d* = 0.22), and the difference was not statistically significant (*t*(77) = 1.97, *p_adj_* = 0.2); the smallest difference was between the gold standard and adding TIV as a covariate (*d* = 0.09). The mean adjusted R^2^ was higher for the proportions method (mean adjusted R^2^ = 0.21), followed by adding TIV as a covariate (mean adjusted R^2^ = 0.059); however, when performing a partial correlation to control for the effect of TIV before computing the R^2^ for the proportions method, the R^2^ value decreased from 0.21 to 0.006 (*t* = 25.8, *p* < 0.001, *d* = 2.92), suggesting that dividing DBM by TIV introduced information about TIV otherwise not present. The R^2^ difference between not using any correction and adding TIV as a covariate was statistically significant and had a moderate effect size (*t*(77) = -5.34, *p_adj_* < 0.001; *d* = -0.6). Interestingly, as opposed to the regional findings, the proportions method did not reverse the trend for the sex estimates but exaggerated the differences. It is worth noting that the normalization that results from linear registration to the ICBM-template is not equivalent to using the proportions method on volumetric information extracted in the participants’ native space (Supplementary Fig. 2).

#### 3.1.3 Regional surface area

**Fig. 3.**
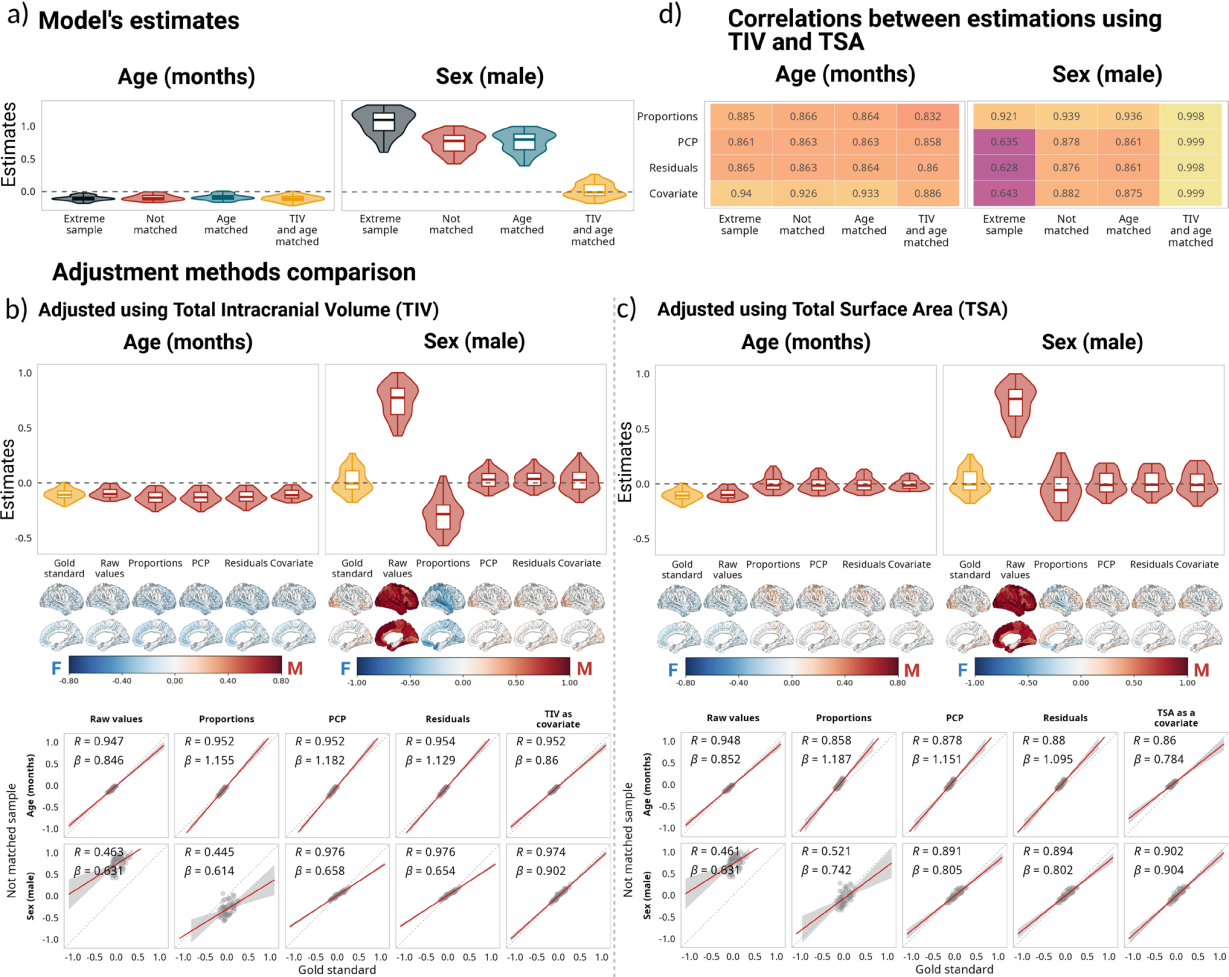
Impact of correction methods on model estimations based on surface area. (a) Distribution of estimates for all samples, based on the following model SA ~ 1 + age + sex + age:sex. Note that for all cases, the estimates for age are negative, small, and follow the same trend in the four samples. (b, c) Comparison of estimates for the matched sample without adjustment and the estimates after using different adjustment methods for the not-matched sample. (b) Presents the estimates using TIV for the corrections, and (c) reflects the same results using TSA. Below the estimates are the corresponding cortical projections of the same values. On the bottom, the correlations between estimates for the matched sample without adjustment and the estimates after using different adjustment methods for the not-matched sample. (d) Correlations between the estimates using different global measures (i.e., TIV and TSA) for adjustment. For a comparison of all the estimates using TIV as the adjustment metric, see Supplementary Figures 4 and 5 for comparisons using TSA.

SA estimates followed a very similar pattern to those of the regional volumes, albeit they only included the 62 cortical regions provided in the DKT atlas. Age was negatively associated with SA for all brain regions and samples. Sex estimations differed across samples, following the same pattern as the regional volumes (Fig. 3a). While none of the adjustment methods had a significant effect on the age estimates, in the case of the sex estimates, the proportions method reversed the trends for the non-matched, age-matched, and extreme sample, with all estimates being negative. PCP, residuals, and adding TIV as a covariate yielded similar results, which were highly correlated with the estimates obtained for the matched sample (Fig. 3b). For the sex estimate, there was no statistically significant difference between the estimates obtained in the matched sample versus the estimates obtained when we added TIV as a covariate (*t*(61) = -1.15; *p_adj_* = 0.25), and the effect size of the difference was negligible (*d* = -0.146). The effect sizes for the PCP and residuals methods were small (*d* = -0.27, *d* = -0.37, respectively); however, the difference between the gold standard and PCP was not statistically significant (*t*(61) = -2.12; *p_adj_* = 0.11), while the difference between the gold standard and the residuals method was significant (*t*(61) = -2.9; *p_adj_* = 0.02). In the case of age, the effect size of the difference between the estimates in the gold standard and in the not-matched sample without adjustment was large (*d* = -0.85), despite the values having a high correlation and low degree of bias (⍴ = 0.97, β = 0.98); the smallest difference was between the gold standard and adding TIV as a covariate (*d* = -0.16). The mean adjusted R^2^ was higher for adding TIV as a covariate (mean adjusted R^2^ = 0.35) than for any of the other methods, including not using any correction (mean adjusted R^2^ = 0.15). The R^2^ difference between not using any correction and adding TIV as a covariate was statistically significant and had a large effect size (*t*(61) = -22.26, *p_adj_* < 0.001; *d* = -2.82).

While the correlation values were smaller, the results were also very similar when TSA was used for adjustment instead of TIV (r ~ 0.9 versus 0.97 for PCP, residuals, and covariates; Fig. 3b bottom for the adjustment with TIV, and Fig. 3c bottom for the adjustment with TSA). We further examined the correlation between the estimates obtained while using the two different metrics as adjustment variables. For the age estimate, the correlations for all samples and methods were over ⍴ = 0.82, with adding the adjusting variable as a covariate showing the highest correlations (⍴ = 0.88 for the matched sample, ⍴ = 0.92 for the not matched, and ⍴ = 0.94 for the extreme sample; Fig. 3d top panel). For the sex estimate, the proportions method had the highest correlation (over ⍴ = 0.92 for all samples). From the other three methods, adding the adjusting variable as a covariate had the highest correlation in all samples. Interestingly, the correlations for the three methods were lower for the extreme sample and had the highest values for the matched sample (Fig. 3d bottom panel).

#### 3.1.4 Regional cortical thickness

**Fig. 4.**
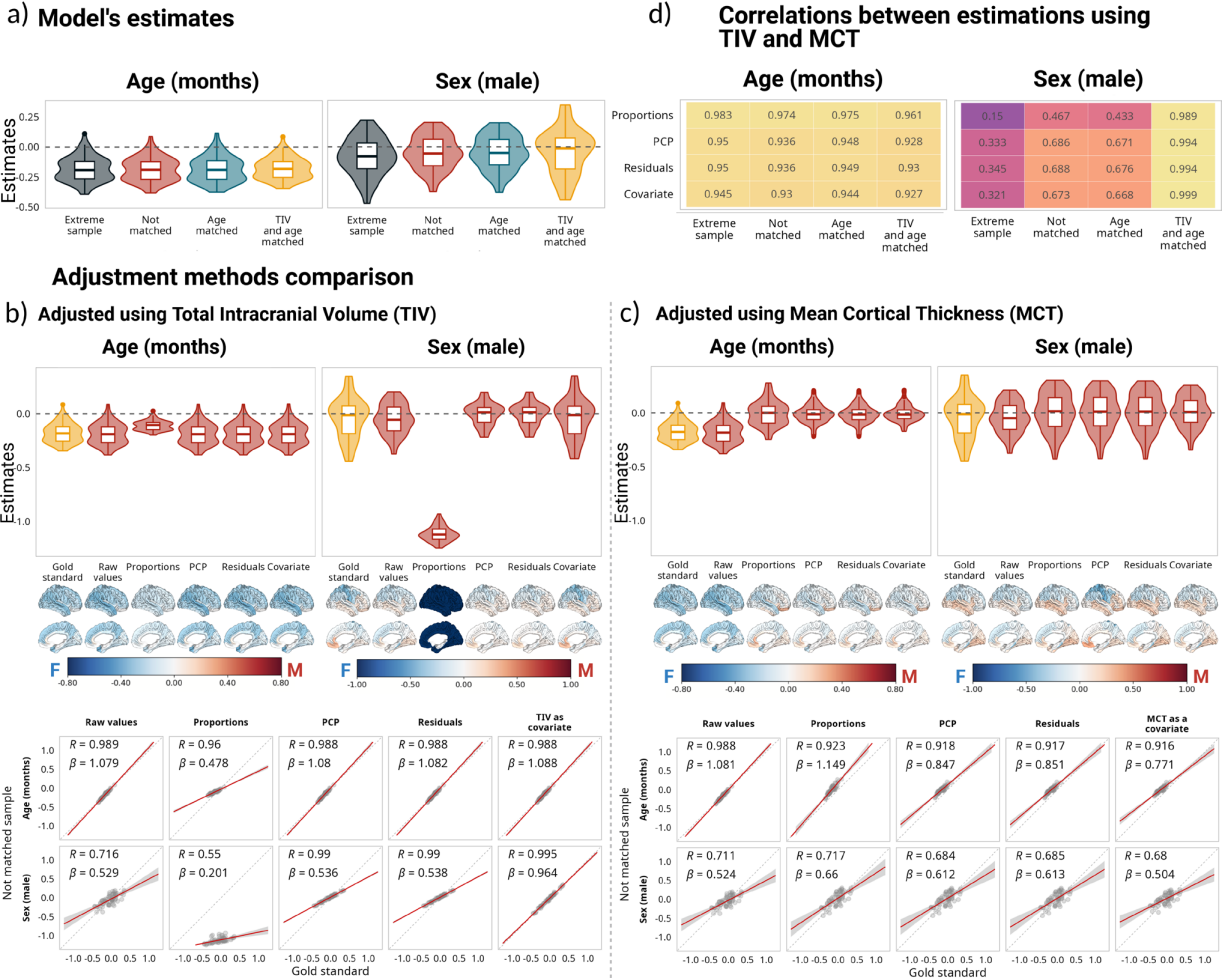
Impact of correction methods on model estimations based on cortical thickness. (a) Distribution of estimates for all samples, based on the following model CT ~ 1 + age + sex + age:sex. (b, c) Comparison of estimates for the matched sample without adjustment and the estimates after using different adjustment methods for the not-matched sample. (b) Presents the estimates using TIV for the corrections, and (c) reflects the same results using TSA. Below the estimates are the corresponding cortical projections of the same values. On the bottom, the correlations between estimates for the matched sample without adjustment and the estimates after using different adjustment methods for the not-matched sample. (d) Correlations between the estimates using different global measures (i.e., TIV and MCT) for adjustment. For a comparison of all the estimates using TIV as the adjustment metric, see Supplementary Figures 6 and 7 for comparisons using MCT.

Regional CT was negatively associated with age across all samples. Sex estimates in the age-matched, not-matched, and extreme samples leaned toward negative values, suggesting that CT is overall larger in females, while in the matched sample, this distribution was centered around zero (Fig. 4a). Proportions was the only adjustment method that affected the age estimations. Both PCP and residuals method estimates were highly correlated with the gold standard; however, using TIV as a covariate yielded better results, without leading to a high degree of over- or underestimations (Fig. 4b). Note the regression slopes in Figure 4b (bottom) for which TIV yielded β values close to one, indicating excellent correspondence to gold standard estimates. For the sex estimate, there was no statistically significant difference between the estimates obtained in the matched sample versus the estimates obtained in the not-matched sample without using corrections (*t*(61) = 0.39; *p_adj_* = 1), nor when TIV was added as a covariate (*t*(61) = -0.45; *p_adj_* = 1), and the effect size of the difference was negligible in both cases (*d* = 0.05 and *d* = -0.05 correspondingly). The effect sizes for the PCP and residuals methods were small (*d* = -0.45, *d* = -0.45, respectively), and the difference was statistically significant (*t*(61) = -3.53, *p_adj_* < 0.01, and *t*(61) = -3.57, *p_adj_* < 0.01); meanwhile the effect size of the difference between the gold standard and the proportions method was large (*d* = 6.66) and the difference was statistically significant (*t*(61) = 52.51, *p* < 0.001). In the case of age, the effect size of the difference between the estimates in the gold standard and in the not-matched sample without adjustment was moderate (*d* = 0.5); the smallest difference was between the gold standard and adding TIV as a covariate, and with PCP (*d* = 0.47 in both cases). The mean adjusted R^2^ was higher for the proportions method (mean adjusted R^2^ = 0.34), followed by adding TIV as a covariate (mean adjusted R^2^ = 0.06); however, when performing a partial correlation to control for the effect of TIV before computing the R^2^ for the proportions method, the mean R^2^ value decreased from 0.33 to 0.014 (*t* = 70.3, *p* < 0.001, *d* = 8.93), suggesting that dividing DBM by TIV introduced information about TIV otherwise not present. The R^2^ difference between not using any correction and adding TIV as a covariate was statistically significant and had a large effect size (*t*(61) = -6.69, *p_adj_* < 0.001; *d* = -0.85). Using MCT as the adjustment metric led to a shift in the age estimates (Fig. 4c top left panel). Sex estimates were centered around zero for all methods; however, the correlations with the gold standard were below ⍴ = 0.7.

### 3.2 Vertexwise analyses

We extracted cortical metrics for 327,684 vertices. However, only 298,901 (87.8%) of these were analyzed, including only vertices for which at least 80% of the participants in the full sample had non-missing values across the different smoothing levels.

#### 3.2.1 Vertexwise surface area

**Fig. 5.**
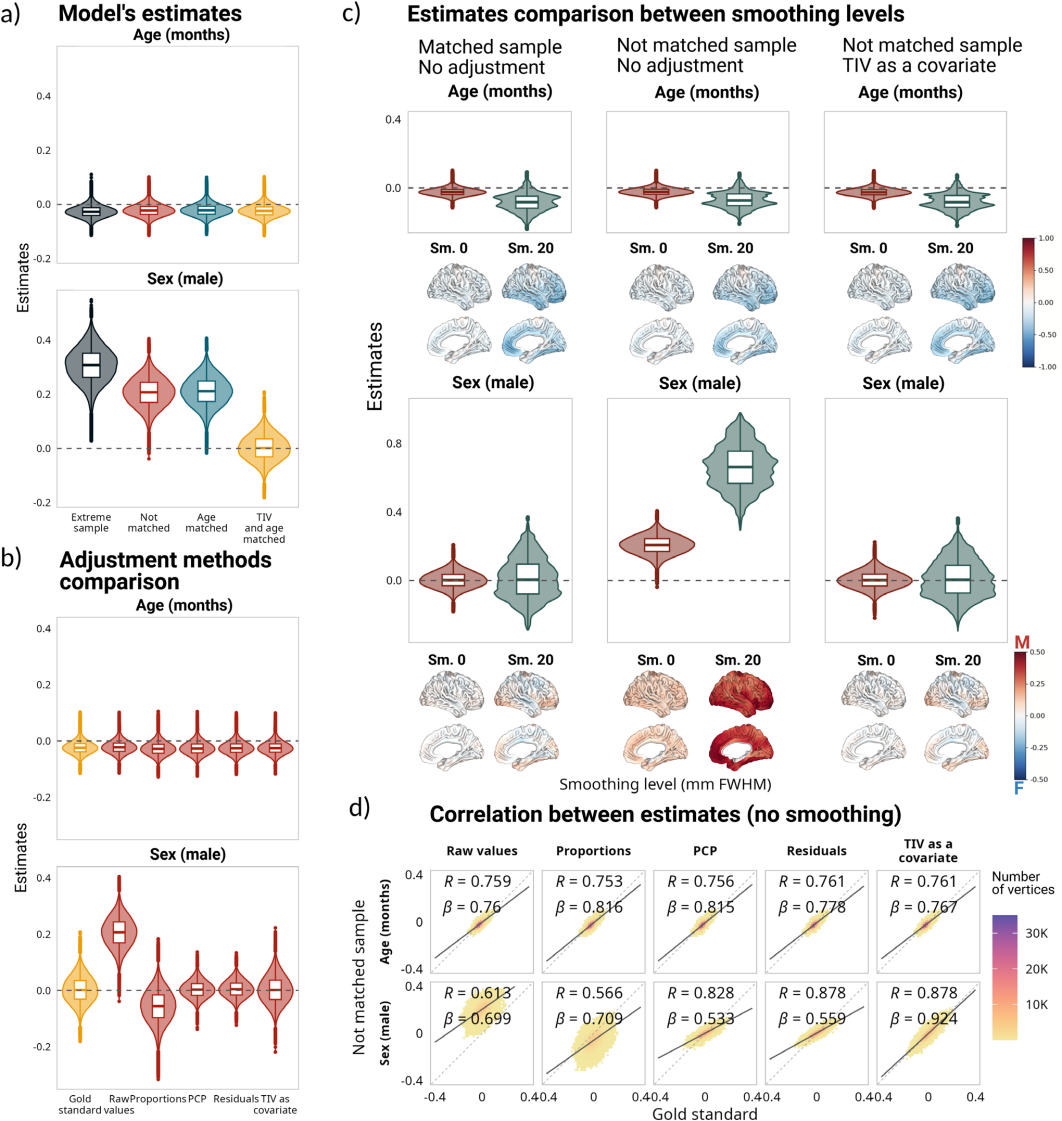
Impact of correction methods on model estimations based on vertexwise surface area with and without smoothing. (a) Distribution of estimates for all samples, for the following model SA ~ 1 + age + sex + age:sex, on data without smoothing. (b) Comparison of estimates for the matched sample without adjustment and the estimates after using different adjustment methods for the not-matched sample. (c) Comparison between the distribution of the estimates using data without smoothing versus data smoothed at 20 mm FWHM. Below each estimate are the corresponding cortical projections of the same values. (d) Correlations between estimates for the matched sample without adjustment and the estimates after using different adjustment methods for the not-matched sample. The analyses performed on data smoothed at 20 mm FWHM are included as Supplementary Figures 13 to 16.

For 0 mm smoothing, SA estimates followed the same pattern as that of regional SA estimates (Fig. 5a; full comparison in Supplementary Fig. 8). None of the adjustment methods had a distinct effect on the age estimations when TIV was used as the adjustment metric (Fig. 5b), but they shifted from more negative values to being centered around zero when TSA was used for correction (Supplementary Fig. 9). Residuals, PCP, and adding TIV as a covariate centered the sex estimates around zero, mirroring the results obtained in the matched sample. Nonetheless, both residuals and PCP led to a decrease in the range of the estimates, with the same biased underestimation as discussed before (Fig. 5d). Across the cortex, the adjusted sex estimates correlated with gold standard estimates with ⍴ = 0.82 for PCP, and ⍴ = 0.87 for residuals and adding TIV as a covariate (Fig. 5d). For the sex estimate, the effect size of the difference between the matched sample versus the estimates obtained when we added TIV as a covariate was negligible (*d* = -0.008), as well as for the PCP and residuals methods (*d* = -0.04, *d* = -0.1, respectively). In the case of age, the effect size of the difference between the estimates in the gold standard and in the not-matched sample without adjustment was negligible (*d* = -0.11); the smallest difference was between the gold standard and adding TIV as a covariate (*d* = -0.08). The mean adjusted R^2^ was higher for adding TIV as a covariate (mean adjusted R^2^ = 0.03) than for any of the other methods, including not using any correction (mean adjusted R^2^ = 0.01). The R^2^ difference between not using any correction and adding TIV as a covariate had a large effect size (*d* = -2.39).

Interestingly, using TSA as the adjustment variable yielded very similar results in terms of correlations and biases; however, in this case, the proportions method also shifted the sex estimates to be centered around zero. Despite this, it is worth noting that the correlations with the estimates obtained in the matched sample were lower (⍴ = 0.58 for proportions, ⍴ = 0.75 PCP, ⍴ = 0.81 for residuals, and ⍴ = 0.82 for adding TSA as a covariate; see Supplementary Fig. 10. Supplementary Fig. 34 includes the correlations between the estimates obtained using TIV and TSA as the global metric).

For the impact of smoothing on the results, the data smoothed at 5–25 mm FWHM yielded similar results regarding the general observed patterns (Supplementary Fig. 11 for adjustments with TIV, and Supplementary Fig. 12 for adjustments with TSA). Here, we use the no smoothing (0 mm) and 20 mm FWHM smoothing levels to highlight the overall findings from this analysis (Fig. 5c). We observed four remarkable differences: First, the magnitude of the estimates, and thus variance increased with increase in smoothing levels, which led to large biases in regional sex estimations. Second, when using a 20 mm FWHM smoothing level, the correlations between gold standard and not-matched sample estimates increased to ⍴ > 0.95 for the age and sex estimates after applying different correction methods (except for the proportions method) when using TIV as the adjustment variable, and ⍴ > 0.9 when using TSA (Supplementary Figs. 15 and 16). Third, the effect sizes of the differences between the estimates obtained as the gold standard and in the matched sample with adjusted data increased with an increase in smoothing. Fourth, for all the adjustment methods, the adjusted R^2^ value had an increase of an order of magnitude comparing no smoothing with a smoothing of 20 mm FWHM. Note that the analyses performed on vertexwise cortical volumes yielded very similar results to those obtained for surface area, and are included as Supplementary Figures 17 to 24 for brevity.

#### 3.2.2 Vertexwise cortical thickness

**Fig. 6.**
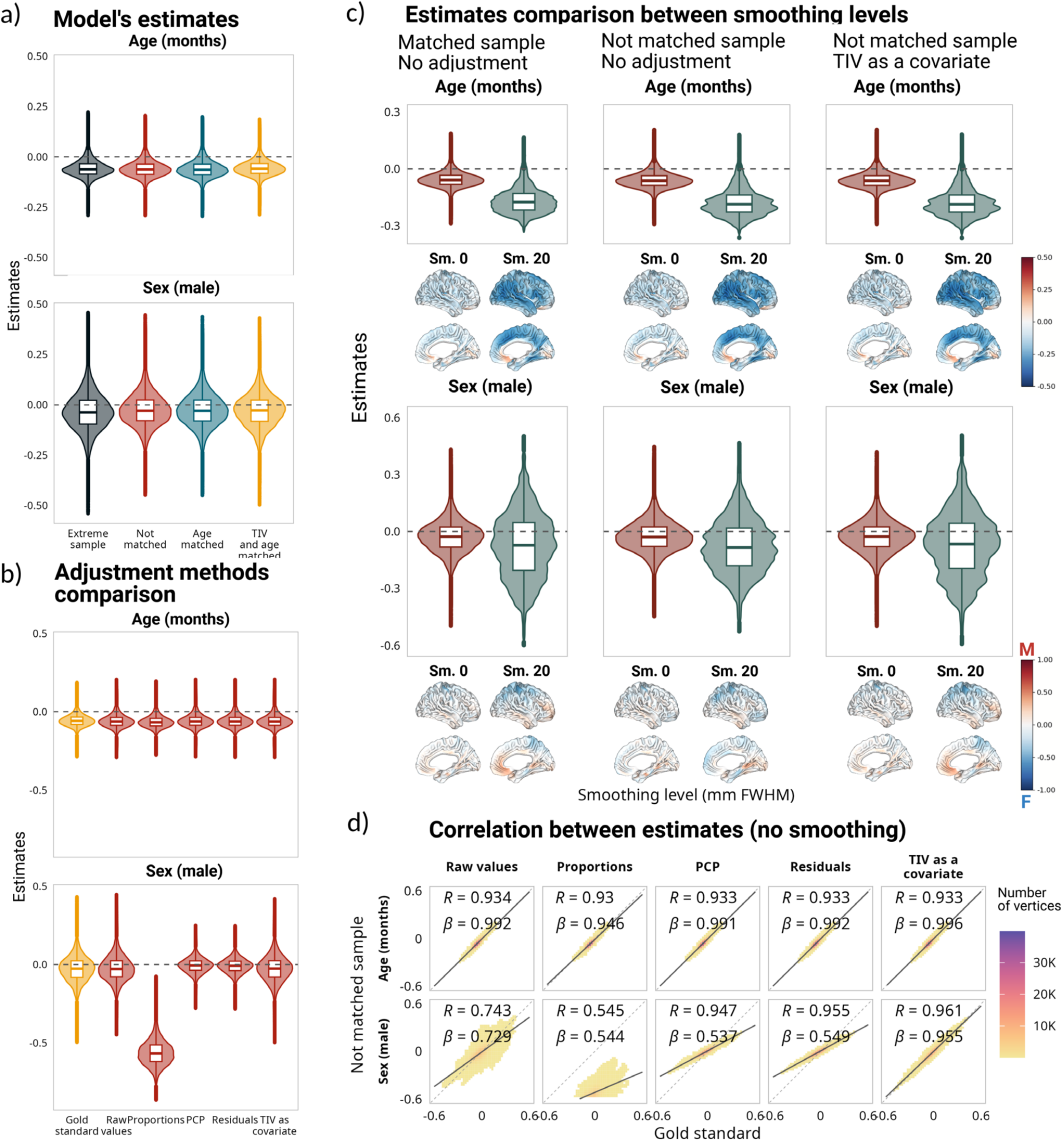
Impact of correction methods on model estimations based on vertexwise cortical thickness with and without smoothing. (a) Distribution of estimates for all samples, for the following model CT ~ 1 + age + sex + age:sex, on data without smoothing. (b) Comparison of estimates for the matched sample without adjustment and the estimates after using different adjustment methods for the not-matched sample. (c) Comparison between the distribution of the estimates using data without smoothing versus data smoothed at 20 mm FWHM. Below each estimate are the corresponding cortical projections of the same values. (d) Correlations between estimates for the matched sample without adjustment and the estimates after using different adjustment methods for the not-matched sample. For additional information on the analyses performed with data smoothed at 20 mm FWHM and using MCT, see Supplementary Figures 25 to 33.

For 0 mm FWHM smoothing, vertexwise CT estimates followed the same pattern across samples as those of regional CT. In contrast to the findings for SA, the general pattern was similar across samples, even for the sex coefficient (Fig. 6a; full comparison in Supplementary Fig. 25). Using TIV as the adjusting metric, applying different adjustment methods did not have a significant effect on the age estimations. However, using MCT for adjustment led to a shift from more negative values to values centered around zero (Supplementary Fig. 26). The residuals and PCP methods centered the sex estimates around zero, mirroring the results obtained in the matched sample, and further led to a decrease in the variability of the estimates. Across the cortex, the adjusted sex estimates correlated with gold standard estimates with ⍴ = 0.94 for PCP, ⍴ = 0.95 for residuals, and ⍴ = 0.96 for adding TIV as a covariate (Fig. 6d). Using MCT as the adjustment variable led to different results, with all the methods centering the sex estimations around zero.

Using TIV as the adjustment variable, for the sex estimate, the effect size of the difference between the matched sample versus the estimates obtained when we added TIV as a covariate was negligible (*d* = -0.06), and it was small for the PCP and residuals methods (*d* = -0.5, *d* = -0.46 respectively); however, it was large for the proportions method (*d* = 6.51). In the case of age, the effect size of the difference between the estimates in the gold standard and in the not-matched sample without adjustment was negligible (*d* = 0.19), as well as the difference between the gold standard and adding TIV as a covariate (*d* = 0.19). The mean adjusted R^2^ was higher for the proportions method (mean adjusted R^2^ = 0.08), followed by adding TIV as a covariate (mean adjusted R^2^ = 0.009). The R^2^ difference between not using any correction and adding TIV as a covariate had a small effect size (*d* = -0.49).

For the impact of smoothing on the results, the data smoothed at 5–25 mm FWHM revealed that increase in smoothing leads to (i) an increase in the variability of the estimates, (ii) the age and sex estimates leaning toward more negative values in both matched and non-matched samples (Supplementary Fig. 32), (iii) an increase in the effect size of the differences between the gold standard estimates and the estimates obtained after adjusting the CT, and (iv) an increase in the adjusted R^2^ value. Contrary to the smoothing-dependent bias in sex estimations in SA, applying different adjustment methods to the smoothed data resulted in the same trends previously observed in non-smoothed data across smoothing levels, indicating minimal impact of smoothing on CT results after correction.

### 3.3 Voxelwise DBM

**Fig. 7.**
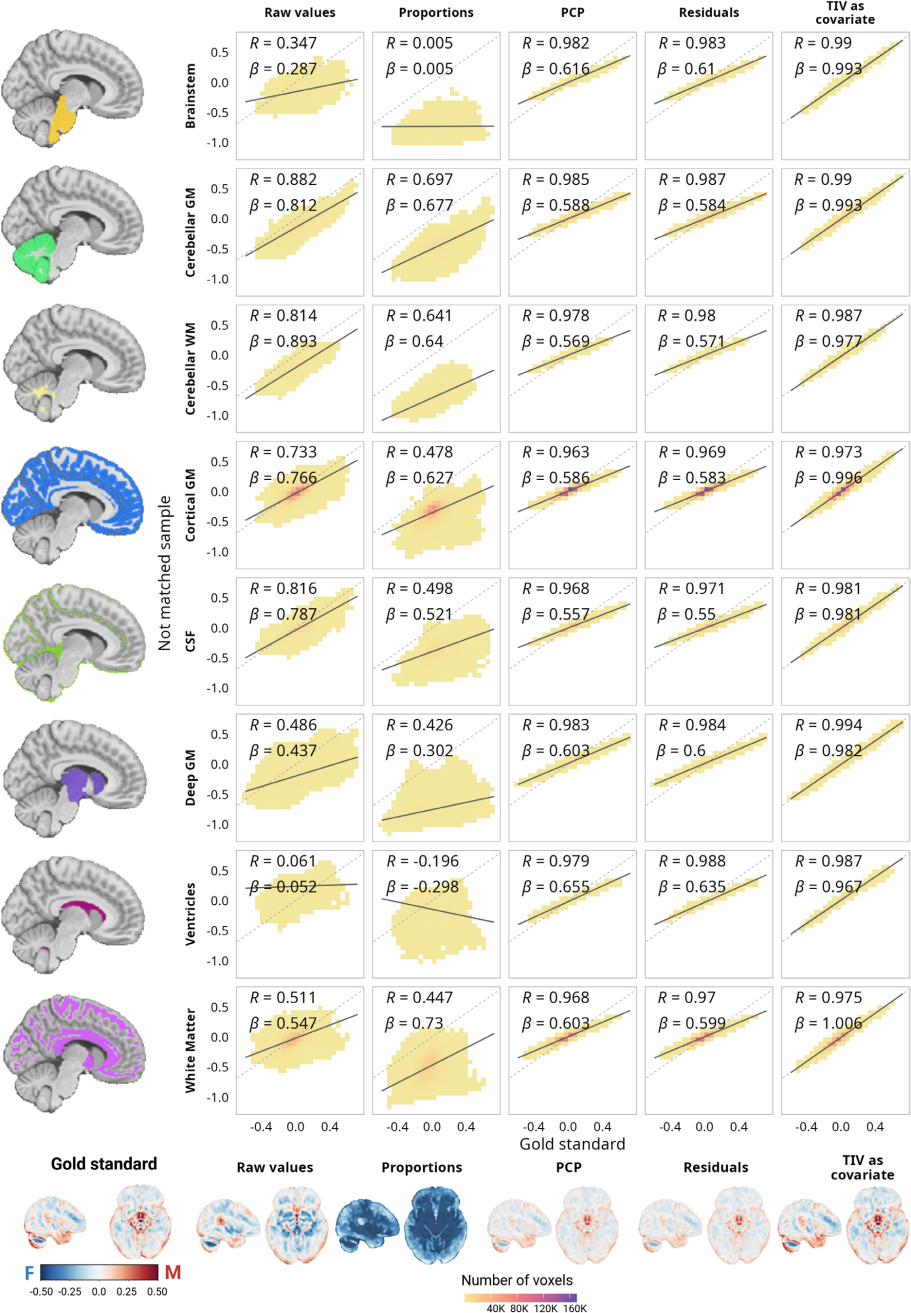
Correlations between the sex estimates for the matched sample without adjustment and the estimates after using different adjustment methods for the not-matched sample using voxelwise deformation-based morphometry (DBM) values across different tissue types. All the brain projections are in the same color scale. The same correlations for the age estimates are presented in Supplementary Figure 38. For a full comparison between the samples and methods in gray and white matter, see Supplementary Figures 35 to 37.

For voxelwise DBM values, instead of only focusing on the gray matter (GM), we analyzed the whole brain using BISON (Dadar & Collins, 2021) segmentation, which provided further insight in both GM and white matter (WM). In both GM and WM, the age estimates leaned toward negative values across all samples. Cortical GM sex estimates (Supplementary Fig. 35) were highly correlated between the matched and the non-matched sample (⍴ = 0.73; Fig. 7); however, most estimates were over- or underestimated in the not-matched sample (note the regression slopes presented in Fig. 7). A similar trend was present in the deep GM (Supplementary Fig. 36), while in this case, the correlation was ⍴ = 0.48 and the estimates were mostly underestimated in the non-matched sample in comparison with the gold standard. WM estimates of the two samples were correlated at ⍴ = 0.51 (Fig. 7), and were either over- or underestimated in the non-matched sample (Supplementary Fig. 37). Interestingly, in the case of the ventricles (i.e., areas that can be indicative of atrophy), there were no correlations between the sex estimates obtained for the two samples (Fig. 7).

The different correction methods behaved similarly at voxel level across different tissues. First of all, the adjustments did not have a significant impact on the age estimations (Supplementary Fig. 38). Across the white and gray matter, the effect size of the difference between the gold standard estimates and the estimates obtained after adjustment was *d* < 0.3 (i.e., small or negligible). In the case of the sex estimates, the proportions method not only reversed the direction of the estimations but also inflated them (*d* = 2.92 for cortical GM, *d* = 3.82 for deep GM, and *d* = 2.86 for WM). Both residuals and PCP methods yielded very similar results, with ⍴ > 0.96 across all tissues. However, they led to a decrease in variability across the estimates and the values were still over- or underestimated. Adding TIV as a covariate generated the optimal results, with a correlation between the non-matched sample and the gold standard of ⍴ > 0.97 across all the tissue types, as well as almost no over- or underestimation bias.

### 3.4 Biases in the estimations

**Fig. 8.**
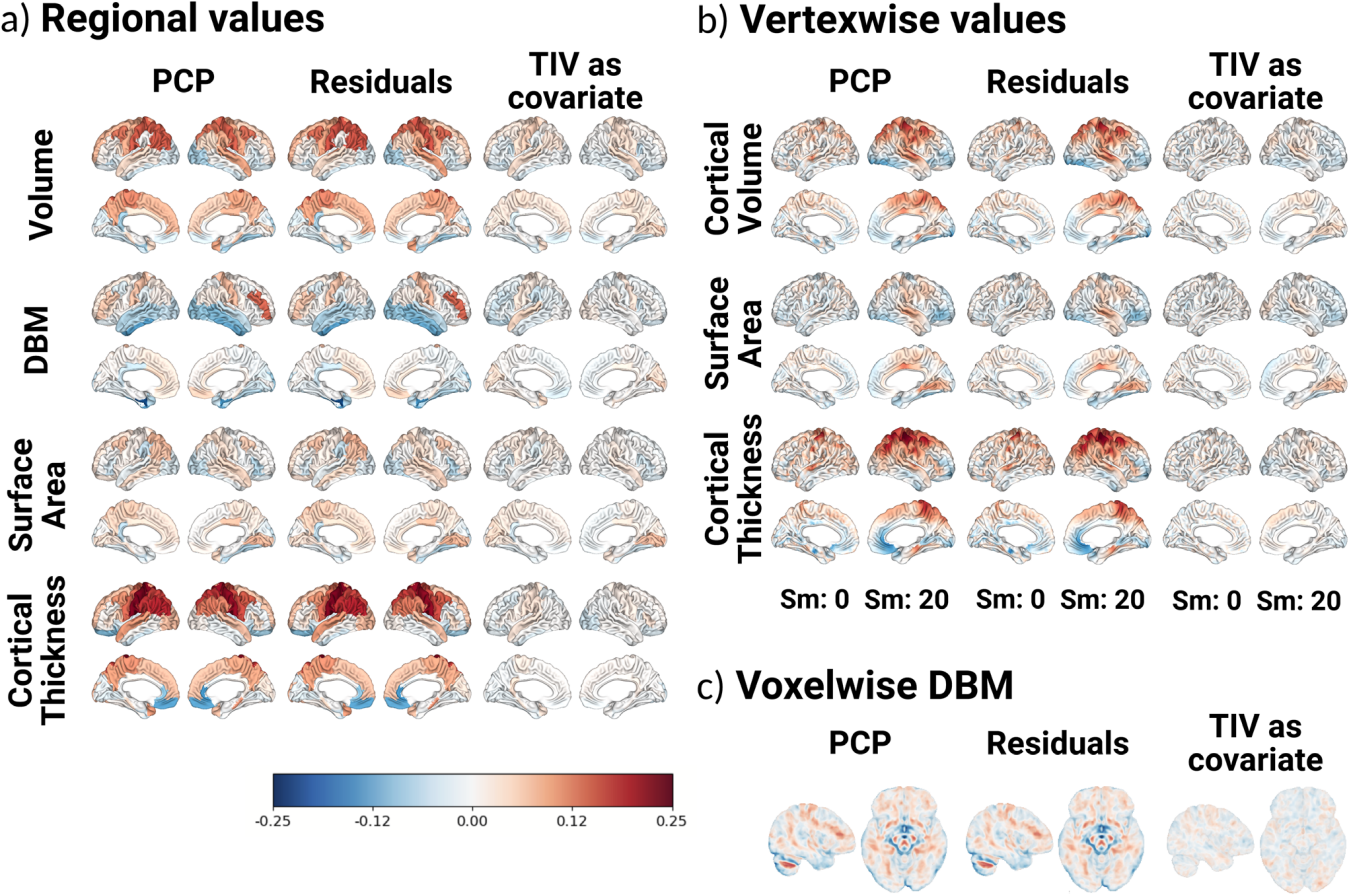
Residual biases in sex estimations after the implementation of the different correction methods. (a) Biases in the regional estimations. (b) Biases on the vertexwise estimations. For each correction method, the left hemisphere (on the left) represents the biases when the data were not smoothed, and on the right hemisphere, when the data were smoothed 20 mm FWHM. (c) Biases at the voxel level based on deformation-based morphometry data.

Finally, we assessed the bias on the sex estimations that remained after using each correction method (using TIV as the adjusting variable), in terms of both magnitude and spatial distribution. Figure 8 shows the results at the regional, vertexwise, and voxelwise analysis levels. Overall, the spatial distribution of the biases was similar across the methods. However, in terms of magnitude, adding TIV as a covariate was best at removing the biases from estimates across the brain. Residuals and PCP results were virtually identical and both left higher biases after correction than after the TIV adjustments, while following a similar pattern to it. Interestingly, at both regional and vertex levels, biases in corrected estimates were larger for cortical thickness and smaller for surface area, suggesting a more linear scaling in the case of surface area across the regions. For the volumetric and cortical thickness data, the biases were mainly localized on dorsal areas, while for regional DBM, we observed a positive bias (undercorrection, i.e., a higher bias toward male estimates than suggested by the gold standard) on temporal regions when using the residuals or PCP methods. For volumetric and DBM data, overall, the bias was stronger in subcortical regions. It is worth noting that for vertexwise data, higher smoothing levels were associated with increased biases, however, adding TIV as a covariate reduced the bias at higher smoothing levels.

Additionally, we evaluated the relationship between the residual bias maps and allometry estimates for the volumetric regional data. The results are presented in Table 4 (for a representation of the correlations and null distribution, see Supplementary Fig. 39). Regardless of the allometry map used, the residual biases after utilizing the PCP and residuals methods were positively correlated with allometry, and this correlation was statistically significant (considering the empirical *p*-values derived from the null distribution). Meanwhile, the residual bias after adding TIV as a covariate was not significantly correlated with allometry. Utilizing the same procedure, we observed that the residual bias maps for the PCP, residuals, and covariate methods had a statistically significant correlation (*p_spin_* < 0.05). Furthermore, the correlation between the residual bias map for the PCP and residuals method was ⍴ = 0.99, and the correlations between those and the residual bias map of adding TIV as a covariate were ⍴ = 0.74 for both cases.

**Table 4.** Correlation and spin test between regional volumes bias maps and allometry estimates.

	Allometry estimate: matched sample		Allometry estimate: not-matched sample	
Bias map (method)	Female estimate	Male estimate	Female estimate	Male estimate
Power-corrected proportions	r: 0.62, p_spin_: 0.002	r: 0.59, p_spin_: 0.005	r: 0.59, p_spin_: 0.003	r: 0.61, p_spin_: 0.002
Residuals	r: 0.64, p_spin_: 0.001	r: 0.6, p_spin_: 0.004	r: 0.61, p_spin_: 0.002	r: 0.63, p_spin_: 0.001
Adding TIV	r: 0.36, p_spin_: 0.119	r: 0.35, p_spin_: 0.129	r: 0.33, p_spin_: 0.151	r: 0.36, p_spin_: 0.103

### 3.5 Additional samples validation

We evaluated whether the matching process might have introduced potential biases in the subsamples by assessing whether the matched sample was representative of the whole population. First, we performed chi-squared tests to compare the prevalence of hypertension, diabetes, cardiovascular disease, and dyslipidemias, between the matched sample and the remaining participants, and did not observe statistically significant differences (*p* > 0.05). Second, we assessed height and BMI. As expected, females in the matched sample were taller than the unmatched participants (*t*(10,548) = 17.32, *p* < 0.001), and males in the matched sample were shorter than the unmatched participants (*t*(11,511) = 17.96, *p* < 0.001); however, the effect sizes of these differences were small (females: *d* = -0.27; males: *d* = 0.29). We found no statistically significant BMI difference for both sexes combined (*t*(21,605) = -0.41571, *p* = 0.67, *d* = -0.004), females (*t*(10,148) = 1.23, *p* = 0.21, *d* = 0.019), or males (*t*(11,317) = -0.66, *p* = 0.50, *d* = -0.011), and the effect sizes were negligible. Finally, there were no statistically significant differences between the two groups in educational level (*X^2^*(11, *N* = 35,732) = 17.02, *p* = 0.1) or socioeconomic status (*X^2^*(7, *N* = 35,732) = 12.01, *p* = 0.1).

Additionally, since the UKBB is an epidemiological dataset, we evaluated the presence of neurodegenerative disorders in our sample. We found that from the 35,732 participants originally included in this study, only 17 (0.05%) had a diagnosis of dementia before the MRI acquisition date, and 71 participants (0.19%) had a diagnosis of Parkinson’s disease or Secondary Parkinsonism. There were no statistically significant differences in the presence of dementias between the matched sample and the rest of the sample (*X^2^*(1, *N* = 35,732) ≤ 0.001, *p* = 1), nor for Parkinson’s disease and Secondary Parkinsonism (*X^2^*(1, *N* = 35,732) ≤ 0.001, *p* = 1). Due to this very low prevalence, we did not expect these cases to have a severe impact on our study. We confirmed this by performing the same analyses for all the regional volumetric data, removing all participants with a dementia, a Parkinson’s disease, or a Secondary Parkinsonism diagnosis, and found that for all the samples and all the adjustment methods, the models’ estimates did not change (⍴ > 0.99 in all cases) compared with the original estimates without removing these participants.

### 3.6 TIV estimate selection

**Fig. 9.**
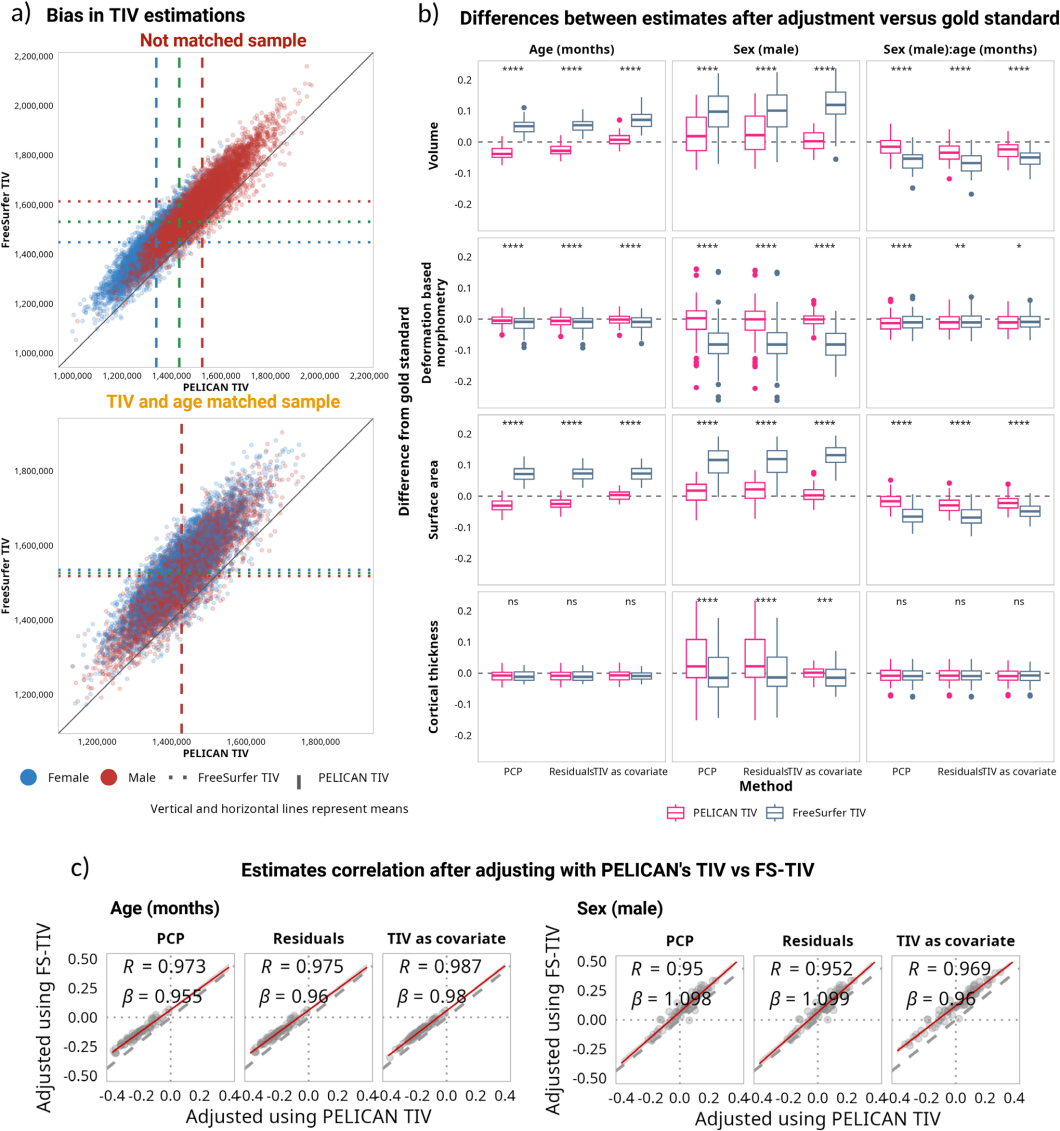
(a) Comparison between PELICAN and FreeSurfer TIV estimates. Top: not-matched sample. Bottom: matched sample. Blue represents females, red represents males, and the green lines represent the whole sample. (b) Comparison of the differences between the standardized estimates in the not-matched sample after adjustment, using PELICAN or FreeSurfer estimations, and the estimates obtained in the matched sample without any adjustment. (c) Correlation between model estimates using PELICAN versus FreeSurfer TIV estimations as the adjustment variable. See Supplementary Figures 40 and 41 for extended versions of the panels.

First, we compared the FreeSurfer TIV estimates versus the TIV values computed by PELICAN. While the two TIVs were strongly correlated (⍴ = 0.94 for the not-matched sample; ⍴ = 0.85 for the matched sample), FS-TIV estimations were generally larger than PELICAN (Fig. 9a) (not matched: *d* = 1.96, *t*(11,293) = 208.35, *p* < 0.001; matched: *d* = 2, *t*(11,293) = 212.52, *p* < 0.001).

For the four regional measures, we repeated the analyses using the FS-TIV, and compared the results with those obtained when using the TIV computed by PELICAN. Figure 9b presents a comparison of the difference between the gold standard estimates and the estimates obtained with adjusted data in the not-matched sample with the two different TIV parameters. In general, across the four metrics, the difference between the gold standard estimates and the adjusted estimates was larger when using FS-TIV than when using PELICAN-TIV, with a few exceptions such as the sex estimate for the proportions method (see Supplementary Fig. 40), and the age and sex interaction term for DBM. Overall, the estimates obtained with both TIV measures were highly correlated for both age and sex (⍴ ≥ 0.95; Fig. 9c). There was also a degree of bias, particularly when adding TIV as a covariate, where age estimates obtained using FS-TIV were smaller than those obtained using PELICAN’s TIV, while the sex estimates tended to be larger. When using the covariate method, the model estimates across the four metrics were statistically different when using PELICAN-TIV and FS-TIV, except for age and age:sex interaction terms for cortical thickness (see Table 5).

**Table 5.** Summary results of paired t-tests and Cohen’s d tests between the estimates obtained for the not-matched sample for all regional metrics when adding TIV as a covariate versus adding FreeSurfer TIV as a covariate.

Metric	Term	t-stat (p_corr)	Cohen’s d
Volume	Age (months)	t: -41.8, (p: <0.001)	d: -4.73 (large)
	Sex (male)	t: -27.79, (p: <0.001)	d: -3.15 (large)
	Sex (male):age (months)	t: 42.03, (p: <0.001)	d: 4.76 (large)
	TIV	t: 14.83, (p: <0.001)	d: 1.68 (large)
Surface Area	Age (months)	t: -41.09, (p: <0.001)	d: -5.22 (large)
	Sex (male)	t: -33.64, (p: <0.001)	d: -4.27 (large)
	Sex (male):age (months)	t: 41.19, (p: <0.001)	d: 5.23 (large)
	TIV	t: 21.95, (p: <0.001)	d: 2.79 (large)
Cortical Thickness	Age (months)	t: -0.13, (p: 0.91)	d: -0.02 (negligible)
	Sex (male)	t: 3.65, (p: 0.001)	d: 0.46 (small)
	Sex (male):age (months)	t: 1.34, (p: 0.219)	d: 0.17 (negligible)
	TIV	t: -5.2, (p: <0.001)	d: -0.66 (moderate)
Deformation-Based Morphometry	Age (months)	t: 5.27, (p: <0.001)	d: 0.6 (moderate)
	Sex (male)	t: 15.74, (p: <0.001)	d: 1.78 (large)
	Sex (male):age (months)	t: -2.19, (p: 0.032)	d: -0.25 (small)
	TIV	t: -16.35, (p: <0.001)	d: -1.85 (large)

## 4 Discussion

In this study, we evaluated the performance of different methods in adjusting MRI-derived brain structural measures to control for the effect of brain size in neuroimaging studies with a focus on age and sex estimation in later adulthood (45–80-year olds). Taking advantage of the UKBB dataset, we implemented a matching approach to create a subsample of participants where females and males were perfectly matched by age and TIV, thus having a **gold standard** to compare four different adjustment methods, namely the proportions, residuals, power-corrected proportions, and adding TIV as a covariate. Each method was evaluated at regional, vertex, and voxel levels, using a consistent linear regression approach for all analyses and comparing the standardized beta coefficients between methods and samples.

Our results suggest that, regardless of the metric of interest, adding TIV as a covariate in a regression model (per region/vertex/voxel) is the best method for removing the effect of TIV on sex differences in aging trajectory models, compared with a gold standard where we enforced the same TIV distribution for both sexes. Importantly, in addition to TIV, we also evaluated using total SA (TSA), for adjusting regional and vertexwise SA, mean CT (MCT) for adjusting CT measures, and total cortical volume for vertexwise volume. In all cases, these metrics impacted the age estimations, suggesting that using these measures for adjustment biases study findings and the results will be suboptimal at best and biased at worst in comparison with TIV-based adjustment. This highlights the importance of using a measure that is consistent across the aging process, instead of one that is also affected by it (Brzezinski-Rittner et al., 2025; Crowley et al., 2018).

Previous studies had evaluated various adjustment methods for volumetric measures in different contexts (Greenberg et al., 2008; Nordenskjöld et al., 2013, 2013; O’Brien et al., 2006, 2011; Pell et al., 2008; Pintzka et al., 2015; Sanchis-Segura et al., 2019, 2020; Sanfilipo et al., 2004; van Eijk et al., 2020; Voevodskaya et al., 2014). The most common comparisons were between proportions and residuals methods, and in some cases, the covariate method. While these studies were mainly based on relatively small sample sizes (under 100 participants), overall, it was recognized that, apart from very specific cases where the goal of the study was to examine differences in the proportion of volume occupied by a certain structure, residuals and covariate methods yielded better results. Most of these studies focused either on a diseased versus control population, or only on sex differences; in this study, we also highlighted how each method affects age estimations in the context of aging studies. In terms of age, the only method that heavily impacted the estimations was the proportions method on DBM and CT estimations at the regional level. We showed that using TIV as a covariate not only properly removes the effect of TIV, but also has a minimal impact on age estimations. This suggests that while sex-related estimates in studies not accounting for TIV-related signals are significantly biased, the age estimates remain largely reliable. Additionally, adding TIV as a covariate has the advantage that, while removing the effect of brain size from other estimations, it also allows us to study how TIV might directly impact the phenomenon of interest with biological significance rather than being a covariate of no interest with an indirect relationship. Further, this method is recommended due to its greater flexibility when it is necessary to incorporate additional covariates (O’Brien et al., 2006; Sanchis-Segura et al., 2019).

To our knowledge, this study is the first attempt to perform a systematic analysis on the effects that different adjustment methods can exert on estimating sex differences using different MRI-derived brain measurements, that is, cortical thickness, surface area, volumes, and DBM compared with gold standard matching method. The effects we observed with surface area were very similar to those observed on volumetric data, which is expected as surface area and brain volume are closely related measures (Im et al., 2008; Sepehrband et al., 2018). Cortical thickness results were particularly interesting, where sex estimate correlations between the uncorrected non-matched and gold standard samples were higher than those of the other metrics, and the impact of using different correction methods was less evident. A potential explanation is that CT is poorly correlated with body and head size measures (Im et al., 2008; Labounek et al., 2025; Schwarz et al., 2016; Tamnes et al., 2010; Vuoksimaa et al., 2018). This is also the first systematic investigation of the effect of brain size adjustments on DBM. DBM processing includes a linear registration step which linearly scales the brain to the stereotaxic space prior to estimating voxelwise atrophy. As seen in the results, this scaling is not equivalent to adjusting for TIV and additional adjustment is necessary.

The various methods evaluated in this study take different elements into consideration. For instance, the proportions method was originally designed to examine the percentage of intracranial volume occupied by a given brain region. In the current study, across all analyses, the proportions correction method tended to result in more negative standardized estimates for sex, indicating larger values for females. This effect was more pronounced for CT and DBM measures, the two metrics that are less correlated with TIV (Dhamala et al., 2022; Im et al., 2008). Likewise, the correlations between the standardized estimates of sex for the models using proportions-adjusted data and the gold standard were consistently the lowest. Interestingly, for CT and DBM, the adjusted R^2^ mean value for the models utilizing proportions was higher than for other adjustment methods, and significantly diminished when we performed a partial correlation to control for the effect of TIV, which, considered alongside the correlation information, suggests that the proportions method might introduce TIV-related biases in measures not heavily impacted by it. Finally, Dahmala et al. (2022) reported that using the proportions method reduces the prediction accuracy for variations in cognitive ability when using gray matter volume or surface area information as features, but increases it when using CT (Dhamala et al., 2022), further suggesting that this method might induce additional information about TIV in CT.

The residuals method was intended for group comparisons in the context of a diseased versus a control population (Arndt et al., 1991; Mathalon et al., 1993), which would potentially hinder its use if the goal of a study is not contrasting different populations. The PCP method is based on considering the non-linear scaling relationship between a brain region and a global size measure (Liu et al., 2014) (i.e., brain allometry; de Jong et al., 2017; Reardon et al., 2018). Importantly, in our previous work, we reported that brain allometry is consistent between sexes and across a wide spectrum of brain sizes (Brzezinski-Rittner et al., 2025). Finally, the covariate method linearly residualizes any metric of interest by the adjustment metric. Interestingly, this suggests that the introduced biases for smaller and larger TIVs are mainly linear regardless of allometry, and this linear relationship can vary between regions. Meanwhile, for the residuals and PCP methods, there is a residual bias associated with allometry, potentially due to bias induced by the corresponding use of the slope of the relationship between the metric of interest and TIV, either as a multiplicative factor in the residuals method or as a power for the denominator (i.e., TIV) in the PCP method. In other words, within the age range under study, a linear relationship is the best representation of the scaling of the brain while neurodevelopmental stages warrant further investigations.

Evaluating the impact of various smoothing levels on vertexwise data, we found that independent of the selected adjustment method, an increase in smoothing level is associated with larger estimates both for age and sex, which leads to an increase in estimates’ variability and biases, in the three analyzed metrics, as well as an improvement in the model’s goodness of fit, as assessed via the adjusted R^2^. One of the primary rationales for applying smoothing kernels is that smoothing reduces individual variability (particularly with larger kernel sizes), thereby increasing the signal-to-noise ratio in group-level analyses. This, in turn, can increase effect sizes and statistical significance (Zeighami & Evans, 2021). Regarding TIV, following the same principle, the estimates become increasingly biased at higher levels of smoothing. Our results further suggest that, in the case of biases in sex difference estimations, regressing out the effect of TIV by adding it as a covariate in a regression model mitigates this effect. This emphasizes the necessity of using reliable correction methods at higher smoothing levels to counteract these biases and obtain reliable estimates. Nevertheless, our analyses provide both a more comprehensive characterization of these biases and guidance for interpreting sex differences as well as TIV-corrected results in future studies. While there is no straightforward answer to what an ideal smoothing level is Zhao et al. (2013), it is advisable to consider the implications of using a given smoothing level for any analysis and considering lower smoothing levels when subtle regional variabilities are of importance to the question of interest.

The comparison of the effect of using FreeSurfer’s estimated TIV versus using the TIV extracted by PELICAN showed that FS-TIV might introduce a bias in model estimations. Additionally, FS-TIV estimations were significantly larger than PELICAN TIV. Importantly, both pipelines use similar methods relying on linear registration to estimate TIV (Buckner et al., 2004; Dadar et al., 2025), but while PELICAN uses the MNI-ICBM2009 average template as registration target, FreeSurfer uses MNI305 (https://surfer.nmr.mgh.harvard.edu/fswiki/eTIV), which is larger. Further, Nordenskjöld et al. (2013) have previously reported that FS-TIV estimation shows a bias dependent on skull size (Nordenskjöld et al., 2013). Hence, we recommend considering these differences when using TIV correction methods.

We also acknowledge the limitations of our study. Despite being a large dataset for neuroimaging studies, the UKBB sample might not be fully representative of the general population, even within the area where it was collected (Ritchie et al., 2018). For instance, it has been noted that compared with the general population, the participants in the UKBB report lower smoking rates and alcohol intake, have fewer self-reported health conditions, are less likely to be obese, and more likely to live in less socioeconomically deprived areas (Littlejohns et al., 2020). Additionally, from the population who participated in the imaging acquisition protocol, over 96% were Caucasian, which limits the generalizability of the findings to other ethnic groups. Furthermore, while our reference gold standard (i.e., the TIV and age-matched sample) was not different from the whole sample in terms of education, socioeconomic level, disease prevalence, or BMI, we acknowledge that it encompasses a reduced range of heights and TIV, excluding the tails of the original distributions (i.e., females with the smallest TIV and males with the largest TIV). Despite this, our previous research (Brzezinski-Rittner et al., 2025) shows that allometry remains consistent across a large range of TIV, suggesting that this effect will not have a strong impact on our results.

Our study has several strengths that support the reliability of our findings. First, we had access to the UK Biobank dataset with over 35,000 imaging data points of participants ranging from 45 to 80 years old, which allowed us to create perfectly matched subsamples with over 10,000 participants in each subsample, thus assuring statistical power and robustness of our results. Second, we consistently used the same analytical methodology (i.e., linear regression) among different adjustment methods and metrics tested, assuring the comparability of our results. Third, visual quality control was part of our image preprocessing pipeline, ensuring the accuracy of the TIV estimations. Furthermore, other potential sources of error, such as excessive motion in the scanner, incidental findings, or incomplete field of view, were also excluded from our analyses.

In conclusion, performing a comprehensive systematic evaluation of four different TIV adjustment methods to assess sex differences in various neuroimaging metrics, we found that adding TIV as a covariate is optimal for removing the effect of brain size from sex differences estimations, at region, vertex, and voxel levels. This effect is more prominent in volumetric, surface area, and DBM calculations than for cortical thickness.

## Supplementary Material

Supplementary Material

Supplementary Table 1

Supplementary Table 2

## Data Availability

The UK Biobank dataset is open access and can be requested from https://www.ukbiobank.ac.uk/use-our-data/apply-for-access/. FreeSurfer is also open source and freely available at https://surfer.nmr.mgh.harvard.edu/. Similarly, PELICAN (Dadar et al., 2025), the image processing pipeline used to derive TIV and DBM measurements, is open source and freely available at https://github.com/VANDAlab/Preprocessing_Pipeline. The code used during this project is available at https://github.com/AgingLab/tiv_adjustment_brzezinskirittner_2026. Estimates and t-value maps obtained for the matched and not matched sample without adjustments and adding TIV as a covariate are available at https://doi.org/10.5281/zenodo.20029615.

## References

[IMAG.a.1235-b1] Alexander-Bloch, A. F., Shou, H., Liu, S., Satterthwaite, T. D., Glahn, D. C., Shinohara, R. T., Vandekar, S. N., & Raznahan, A. (2018). On testing for spatial correspondence between maps of human brain structure and function. NeuroImage, 178, 540–551. 10.1016/j.neuroimage.2018.05.070 29860082 PMC6095687

[IMAG.a.1235-b2] Amaral, R. S. C., Park, M. T. M., Devenyi, G. A., Lynn, V., Pipitone, J., Winterburn, J., Chavez, S., Schira, M., Lobaugh, N. J., Voineskos, A. N., Pruessner, J. C., & Chakravarty, M. M. (2018). Manual segmentation of the fornix, fimbria, and alveus on high-resolution 3T MRI: Application via fully-automated mapping of the human memory circuit white and grey matter in healthy and pathological aging. NeuroImage, Segmenting the Brain, 170, 132–150. 10.1016/j.neuroimage.2016.10.027 27765611

[IMAG.a.1235-b3] Arndt, S., Cohen, G., Alliger, R. J., Swayze, V. W., & Andreasen, N. C. (1991). Problems with ratio and proportion measures of imaged cerebral structures. Psychiatry Research: Neuroimaging, 40(1), 79–89. 10.1016/0925-4927(91)90031-K 1946842

[IMAG.a.1235-b4] Ashburner, J., Hutton, C., Frackowiak, R., Johnsrude, I., Price, C., & Friston, K. (1998). Identifying global anatomical differences: Deformation-based morphometry. Human Brain Mapping, 6(5–6), 348–357. 10.1002/(SICI)1097-0193(1998)6:5/6<348::AID-HBM4>3.0.CO;2-P 9788071 PMC6873376

[IMAG.a.1235-b5] Brzezinski-Rittner, A., Moqadam, R., Iturria-Medina, Y., Chakravarty, M. M., Dadar, M., & Zeighami, Y. (2025). Disentangling the effect of sex from brain size on brain organization and cognitive functioning. GeroScience, 47, 247–262. 10.1007/s11357-024-01486-5 39757311 PMC11872830

[IMAG.a.1235-b6] Buckner, R. L., Head, D., Parker, J., Fotenos, A. F., Marcus, D., Morris, J. C., & Snyder, A. Z. (2004). A unified approach for morphometric and functional data analysis in young, old, and demented adults using automated atlas-based head size normalization: Reliability and validation against manual measurement of total intracranial volume. NeuroImage, 23(2), 724–738. 10.1016/j.neuroimage.2004.06.018 15488422

[IMAG.a.1235-b7] Bycroft, C., Freeman, C., Petkova, D., Band, G., Elliott, L. T., Sharp, K., Motyer, A., Vukcevic, D., Delaneau, O., O’Connell, J., Cortes, A., Welsh, S., Young, A., Effingham, M., McVean, G., Leslie, S., Allen, N., Donnelly, P., & Marchini, J. (2018). The UK Biobank resource with deep phenotyping and genomic data. Nature, 562(7726), 203–209. 10.1038/s41586-018-0579-z 30305743 PMC6786975

[IMAG.a.1235-b8] Coupe, P., Yger, P., Prima, S., Hellier, P., Kervrann, C., & Barillot, C. (2008). An optimized blockwise nonlocal means denoising filter for 3-D magnetic resonance images. IEEE Transactions on Medical Imaging, 27(4), 425–441. 10.1109/TMI.2007.906087 18390341 PMC2881565

[IMAG.a.1235-b9] Crowley, S. J., Tanner, J. J., Ramon, D., Schwab, N. A., Hizel, L. P., & Price, C. C. (2018). Reliability and utility of manual and automated estimates of total intracranial volume. Journal of the International Neuropsychological Society, 24(2), 206–211. 10.1017/S1355617717000868 28978362 PMC7111586

[IMAG.a.1235-b10] Dadar, M., & Collins, D. L. (2021). BISON: Brain tissue segmentation pipeline using T1-weighted magnetic resonance images and a random forest classifier. Magnetic Resonance in Medicine, 85(4), 1881–1894. 10.1002/mrm.28547 33040404

[IMAG.a.1235-b11] Dadar, M., Fonov, V. S., & Collins, D. L. (2018). A comparison of publicly available linear MRI stereotaxic registration techniques. NeuroImage, 174, 191–200. 10.1016/j.neuroimage.2018.03.025 29548850

[IMAG.a.1235-b12] Dadar, M., Manera, A. L., Ducharme, S., & Collins, D. L. (2022). White matter hyperintensities are associated with grey matter atrophy and cognitive decline in Alzheimer’s disease and frontotemporal dementia. Neurobiology of Aging, 111, 54–63. 10.1016/j.neurobiolaging.2021.11.007 34968832

[IMAG.a.1235-b13] Dadar, M., Manera, A. L., Zinman, L., Korngut, L., Genge, A., Graham, S. J., Frayne, R., Collins, D. L., & Kalra, S. (2020). Cerebral atrophy in amyotrophic lateral sclerosis parallels the pathological distribution of TDP43. Brain Communications, 2(2), fcaa061. 10.1093/braincomms/fcaa061 33543125 PMC7846188

[IMAG.a.1235-b14] Dadar, M., Moqadam, R., Metz, A., Chadwick, K., Rittner, A. B., & Zeighami, Y. (2025). PELICAN: A longitudinal image processing pipeline for analyzing structural magnetic resonance images in aging and neurodegenerative disease populations (p. 2025.09.20.677546). bioRxiv. 10.1101/2025.09.20.677546

[IMAG.a.1235-b15] Dadar, M., Narayanan, S., Arnold, D. L., Collins, D. L., & Maranzano, J. (2021). Conversion of diffusely abnormal white matter to focal lesions is linked to progression in secondary progressive multiple sclerosis. Multiple Sclerosis Journal, 27(2), 208–219. 10.1177/1352458520912172 32202199

[IMAG.a.1235-b16] de Jong, L. W., Vidal, J.-S., Forsberg, L. E., Zijdenbos, A. P., Haight, T., Initiative, A. D. N., Sigurdsson, S., Gudnason, V., van Buchem, M. A., & Launer, L. J. (2017). Allometric scaling of brain regions to intra-cranial volume: An epidemiological MRI study. Human Brain Mapping, 38(1), 151–164. 10.1002/hbm.23351 27557999 PMC5148715

[IMAG.a.1235-b17] Deacon, T. W. (1988). Human brain evolution: II. Embryology and brain allometry. In H. J. Jerison & I. Jerison (Eds.), Intelligence and evolutionary biology (pp. 383–415). Springer. 10.1007/978-3-642-70877-0_20

[IMAG.a.1235-b18] Desikan, R. S., Ségonne, F., Fischl, B., Quinn, B. T., Dickerson, B. C., Blacker, D., Buckner, R. L., Dale, A. M., Maguire, R. P., Hyman, B. T., Albert, M. S., & Killiany, R. J. (2006). An automated labeling system for subdividing the human cerebral cortex on MRI scans into gyral based regions of interest. NeuroImage, 31(3), 968–980. 10.1016/j.neuroimage.2006.01.021 16530430

[IMAG.a.1235-b19] Dhamala, E., Ooi, L. Q. R., Chen, J., Kong, R., Anderson, K. M., Chin, R., Yeo, B. T. T., & Holmes, A. J. (2022). Proportional intracranial volume correction differentially biases behavioral predictions across neuroanatomical features, sexes, and development. NeuroImage, 260, 119485. 10.1016/j.neuroimage.2022.119485 35843514 PMC9425854

[IMAG.a.1235-b20] Eliot, L., Ahmed, A., Khan, H., & Patel, J. (2021). Dump the “dimorphism”: Comprehensive synthesis of human brain studies reveals few male-female differences beyond size. Neuroscience & Biobehavioral Reviews, 125, 667–697. 10.1016/j.neubiorev.2021.02.026 33621637

[IMAG.a.1235-b21] Farias, S. T., Mungas, D., Reed, B., Carmichael, O., Beckett, L., Harvey, D., Olichney, J., Simmons, A., & DeCarli, C. (2012). Maximal brain size remains an important predictor of cognition in old age, independent of current brain pathology. Neurobiology of Aging, 33(8), 1758–1768. 10.1016/j.neurobiolaging.2011.03.017 21531482 PMC3177982

[IMAG.a.1235-b22] Fischl, B. (2012). FreeSurfer. NeuroImage, 20 Years of fMRI, 62(2), 774–781. 10.1016/j.neuroimage.2012.01.021 PMC3685476 22248573

[IMAG.a.1235-b23] Greenberg, D. L., Messer, D. F., Payne, M. E., MacFall, J. R., Provenzale, J. M., Steffens, D. C., & Krishnan, R. R. (2008). Aging, gender, and the elderly adult brain: An examination of analytical strategies. Neurobiology of Aging, 29(2), 290–302. 10.1016/j.neurobiolaging.2006.09.016 17049410 PMC2694568

[IMAG.a.1235-b24] Griffiths-King, D., Seri, S., Catroppa, C., Anderson, V. A., & Wood, A. G. (2024). Network analysis of structural MRI predicts executive function in paediatric traumatic brain injury. NeuroImage: Clinical, 44, 103685. 10.1016/j.nicl.2024.103685 39423568 PMC11531611

[IMAG.a.1235-b25] Hentschel, S., & Kruggel, F. (2004). Determination of the intracranial volume: A registration approach. In G.-Z. Yang & T.-Z. Jiang (Eds.), Medical imaging and augmented reality (pp. 253–260). Springer. 10.1007/978-3-540-28626-4_31

[IMAG.a.1235-b26] Huxley, J. S., & Teissier, G. (1936). Terminology of relative growth. Nature, 137(3471), 780–781. 10.1038/137780b0

[IMAG.a.1235-b27] Im, K., Lee, J.-M., Lyttelton, O., Kim, S. H., Evans, A. C., & Kim, S. I. (2008). Brain size and cortical structure in the adult human brain. Cerebral Cortex, 18(9), 2181–2191. 10.1093/cercor/bhm244 18234686

[IMAG.a.1235-b28] Jack, C. R., Twomey, C. K., Zinsmeister, A. R., Sharbrough, F. W., Petersen, R. C., & Cascino, G. D. (1989). Anterior temporal lobes and hippocampal formations: Normative volumetric measurements from MR images in young adults. Radiology, 172(2), 549–554. 10.1148/radiology.172.2.2748838 2748838

[IMAG.a.1235-b29] Kamal, F., Moqadam, R., Morrison, C., & Dadar, M. (2025). Racial and ethnic differences in white matter hypointensities: The role of vascular risk factors. Alzheimer’s & Dementia, 21(3), e70105. 10.1002/alz.70105 PMC11947760 40145319

[IMAG.a.1235-b30] Klasson, N., Olsson, E., Eckerström, C., Malmgren, H., & Wallin, A. (2018). Estimated intracranial volume from FreeSurfer is biased by total brain volume. European Radiology Experimental, 2(1), 24. 10.1186/s41747-018-0055-4

[IMAG.a.1235-b31] Kotikalapudi, R., Kincses, B., Zunhammer, M., Schlitt, F., Asan, L., Schmidt-Wilcke, T., Kincses, Z. T., Bingel, U., & Spisak, T. (2023). Brain morphology predicts individual sensitivity to pain: A multicenter machine learning approach. PAIN, 164(11), 2516. 10.1097/j.pain.0000000000002958 37318027 PMC10578427

[IMAG.a.1235-b32] Kurth, F., Thompson, P. M., & Luders, E. (2018). Investigating the differential contributions of sex and brain size to gray matter asymmetry. Cortex, 99, 235–242. 10.1016/j.cortex.2017.11.017 29287244 PMC5816677

[IMAG.a.1235-b33] Labounek, R., Bondy, M. T., Paulson, A. L., Bédard, S., Abramovic, M., Alonso-Ortiz, E., Atcheson, N. T., Barlow, L. R., Barry, R. L., Barth, M., Battiston, M., Büchel, C., Budde, M. D., Callot, V., Combes, A., De Leener, B., Descoteaux, M., de Sousa, P. L., Dostál, M., … Nestrašil, I. (2025). Body size and intracranial volume interact with the structure of the central nervous system: A multi-center in vivo neuroimaging study. Imaging Neuroscience, 3, imag_a_00559. 10.1162/imag_a_00559 PMC12319740 40800833

[IMAG.a.1235-b34] Lajoie, I., Canadian ALS Neuroimaging Consortium (CALSNIC), Kalra, S., & Dadar, M. (2025). Regional cerebral atrophy contributes to personalized survival prediction in amyotrophic lateral sclerosis: A multicentre, machine learning, deformation-based morphometry study. Annals of Neurology, 97(6), 1144–1157. 10.1002/ana.27196 39985309 PMC12082021

[IMAG.a.1235-b35] Lerch, J. P. (2015). Cortical thickness mapping. Brain Mapping, 1, 351–355. 10.1016/B978-0-12-397025-1.00305-5

[IMAG.a.1235-b36] Lerch, J. P., & Evans, A. C. (2005). Cortical thickness analysis examined through power analysis and a population simulation. NeuroImage, 24(1), 163–173. 10.1016/j.neuroimage.2004.07.045 15588607

[IMAG.a.1235-b37] Lerch, J. P., Worsley, K., Shaw, W. P., Greenstein, D. K., Lenroot, R. K., Giedd, J., & Evans, A. C. (2006). Mapping anatomical correlations across cerebral cortex (MACACC) using cortical thickness from MRI. NeuroImage, 31(3), 993–1003. 10.1016/j.neuroimage.2006.01.042 16624590

[IMAG.a.1235-b38] Littlejohns, T. J., Holliday, J., Gibson, L. M., Garratt, S., Oesingmann, N., Alfaro-Almagro, F., Bell, J. D., Boultwood, C., Collins, R., Conroy, M. C., Crabtree, N., Doherty, N., Frangi, A. F., Harvey, N. C., Leeson, P., Miller, K. L., Neubauer, S., Petersen, S. E., Sellors, J., … Allen, N. E. (2020). The UK Biobank imaging enhancement of 100,000 participants: Rationale, data collection, management and future directions. Nature Communications, 11(1), Article 1. 10.1038/s41467-020-15948-9 PMC7250878 32457287

[IMAG.a.1235-b39] Liu, D., Johnson, H. J., Long, J. D., Magnotta, V. A., & Paulsen, J. S. (2014). The power-proportion method for intracranial volume correction in volumetric imaging analysis. Frontiers in Neuroscience, 8, 97423. 10.3389/fnins.2014.00356 PMC4222222 25414635

[IMAG.a.1235-b40] Luders, E., Gaser, C., Narr, K. L., & Toga, A. W. (2009). Why sex matters: Brain size independent differences in gray matter distributions between men and women. The Journal of Neuroscience, 29(45), 14265–14270. 10.1523/JNEUROSCI.2261-09.2009 19906974 PMC3110817

[IMAG.a.1235-b41] Lüders, E., Steinmetz, H., & Jäncke, L. (2002). Brain size and grey matter volume in the healthy human brain. NeuroReport, 13(17), 2371. 10.1097/00001756-200212030-00040 12488829

[IMAG.a.1235-b42] Luders, E., Toga, A. W., & Thompson, P. M. (2014). Why size matters: Differences in brain volume account for apparent sex differences in callosal anatomy: The sexual dimorphism of the corpus callosum. NeuroImage, 84, 820–824. 10.1016/j.neuroimage.2013.09.040 24064068 PMC3867125

[IMAG.a.1235-b43] Manera, A. L., Dadar, M., Collins, D. L., & Ducharme, S. (2019). Deformation based morphometry study of longitudinal MRI changes in behavioral variant frontotemporal dementia. NeuroImage: Clinical, 24, 102079. 10.1016/j.nicl.2019.102079 31795051 PMC6879994

[IMAG.a.1235-b44] Manera, A. L., Dadar, M., Fonov, V., & Collins, D. L. (2020). CerebrA, registration and manual label correction of Mindboggle-101 atlas for MNI-ICBM152 template. Scientific Data, 7(1), Article 1. 10.1038/s41597-020-0557-9 PMC7363886 32669554

[IMAG.a.1235-b45] Manera, A. L., Dadar, M., Swieten, J. C. V., Borroni, B., Sanchez-Valle, R., Moreno, F., Jr, R. L., Graff, C., Synofzik, M., Galimberti, D., Rowe, J. B., Masellis, M., Tartaglia, M. C., Finger, E., Vandenberghe, R., Mendonca, A. de, Tagliavini, F., Santana, I., Butler, C. R.,…FTLDNI Investigators. (2021). MRI data-driven algorithm for the diagnosis of behavioural variant frontotemporal dementia. Journal of Neurology, Neurosurgery & Psychiatry, 92(6), 608–616. 10.1136/jnnp-2020-324106 33722819

[IMAG.a.1235-b46] Mathalon, D. H., Sullivan, E. V., Rawles, J. M., & Pfefferbaum, A. (1993). Correction for head size in brain-imaging measurements. Psychiatry Research, 50(2), 121–139. 10.1016/0925-4927(93)90016-b 8378488

[IMAG.a.1235-b47] Metz, A., Zeighami, Y., Ducharme, S., Villeneuve, S., & Dadar, M. (2025). Frontotemporal dementia subtyping using machine learning, multivariate statistics and neuroimaging. Brain Communications, 7(1), fcaf065. 10.1093/braincomms/fcaf065 39990273 PMC11844796

[IMAG.a.1235-b48] Misquitta, K., Dadar, M., Louis Collins, D., & Tartaglia, M. C. (2020). White matter hyperintensities and neuropsychiatric symptoms in mild cognitive impairment and Alzheimer’s disease. NeuroImage: Clinical, 28, 102367. 10.1016/j.nicl.2020.102367 32798911 PMC7453140

[IMAG.a.1235-b49] Moqadam, R., Azizi, H., Brzezinski-Rittner, A., Ronat, L. A., Raeesi, S., Hanganu, A., Zeighami, Y., & Dadar, M. (2025). Apathy progression is associated with brain atrophy and white matter damage in Parkinson’s disease. Brain Communications, 7(5) fcaf355. 10.1093/braincomms/fcaf355 41063969 PMC12501503

[IMAG.a.1235-b50] Nordenskjöld, R., Malmberg, F., Larsson, E.-M., Simmons, A., Ahlström, H., Johansson, L., & Kullberg, J. (2015). Intracranial volume normalization methods: Considerations when investigating gender differences in regional brain volume. Psychiatry Research: Neuroimaging, 231(3), 227–235. 10.1016/j.pscychresns.2014.11.011 25665840

[IMAG.a.1235-b51] Nordenskjöld, R., Malmberg, F., Larsson, E.-M., Simmons, A., Brooks, S. J., Lind, L., Ahlström, H., Johansson, L., & Kullberg, J. (2013). Intracranial volume estimated with commonly used methods could introduce bias in studies including brain volume measurements. NeuroImage, 83, 355–360. 10.1016/j.neuroimage.2013.06.068 23827332

[IMAG.a.1235-b52] O’Brien, L. M., Ziegler, D. A., Deutsch, C. K., Frazier, J. A., Herbert, M. R., & Locascio, J. J. (2011). Statistical adjustments for brain size in volumetric neuroimaging studies: Some practical implications in methods. Psychiatry Research: Neuroimaging, 193(2), 113–122. 10.1016/j.pscychresns.2011.01.007 PMC3510982 21684724

[IMAG.a.1235-b53] O’Brien, L. M., Ziegler, D. A., Deutsch, C. K., Kennedy, D. N., Goldstein, J. M., Seidman, L. J., Hodge, S., Makris, N., Caviness, V., Frazier, J. A., & Herbert, M. R. (2006). Adjustment for whole brain and cranial size in volumetric brain studies: A review of common adjustment factors and statistical methods. Harvard Review of Psychiatry, 14(3), 141–151. 10.1080/10673220600784119 16787886

[IMAG.a.1235-b54] Pell, G. S., Briellmann, R. S., Chan, C. H. (Patrick), Pardoe, H., Abbott, D. F., & Jackson, G. D. (2008). Selection of the control group for VBM analysis: Influence of covariates, matching and sample size. NeuroImage, 41(4), 1324–1335. 10.1016/j.neuroimage.2008.02.050 18467131

[IMAG.a.1235-b55] Pintzka, C. W. S., Hansen, T. I., Evensmoen, H. R., & Håberg, A. K. (2015). Marked effects of intracranial volume correction methods on sex differences in neuroanatomical structures: A HUNT MRI study. Frontiers in Neuroscience, 9, 238. 10.3389/fnins.2015.00238 26217172 PMC4496575

[IMAG.a.1235-b56] Planche, V., Manjon, J. V., Mansencal, B., Lanuza, E., Tourdias, T., Catheline, G., & Coupé, P. (2022). Structural progression of Alzheimer’s disease over decades: The MRI staging scheme. Brain Communications, 4(3), fcac109. 10.1093/braincomms/fcac109 35592489 PMC9113086

[IMAG.a.1235-b57] Qiu, T., Liu, Z.-Q., Rheault, F., Legarreta, J. H., Valcourt Caron, A., St-Onge, F., Strikwerda-Brown, C., Metz, A., Dadar, M., Soucy, J.-P., Pichet Binette, A., Spreng, R. N., Descoteaux, M., Villeneuve, S., & PREVENT‐AD Research Group. (2024). Structural white matter properties and cognitive resilience to tau pathology. Alzheimer’s & Dementia, 20(5), 3364–3377. 10.1002/alz.13776 PMC11095478 38561254

[IMAG.a.1235-b58] Rakic, P. (2009). Evolution of the neocortex: A perspective from developmental biology. Nature Reviews Neuroscience, 10(10), 724–735. 10.1038/nrn2719 19763105 PMC2913577

[IMAG.a.1235-b59] Raykov, P. P., Correia, M., Tsvetanov, K., Henriques, R. N., Del Cerro-León, A., Bracher-Smith, M., Escott-Price, V., Raykov, Y. P., & Henson, R. N. (2025). Complementary MR measures of white matter and their relation to cardiovascular health and cognition. Scientific Reports, 15(1), 28890. 10.1038/s41598-025-13610-2 40775500 PMC12332191

[IMAG.a.1235-b60] Reardon, P. K., Seidlitz, J., Vandekar, S., Liu, S., Patel, R., Park, M. T. M., Alexander-Bloch, A., Clasen, L. S., Blumenthal, J. D., Lalonde, F. M., Giedd, J. N., Gur, R. C., Gur, R. E., Lerch, J. P., Chakravarty, M. M., Satterthwaite, T. D., Shinohara, R. T., & Raznahan, A. (2018). Normative brain size variation and brain shape diversity in humans. Science, 360(6394), 1222–1227. 10.1126/science.aar2578 29853553 PMC7485526

[IMAG.a.1235-b61] Ritchie, S. J., Cox, S. R., Shen, X., Lombardo, M. V., Reus, L. M., Alloza, C., Harris, M. A., Alderson, H. L., Hunter, S., Neilson, E., Liewald, D. C. M., Auyeung, B., Whalley, H. C., Lawrie, S. M., Gale, C. R., Bastin, M. E., McIntosh, A. M., & Deary, I. J. (2018). Sex differences in the adult human brain: Evidence from 5216 UK Biobank participants. Cerebral Cortex, 28(8), 2959–2975. 10.1093/cercor/bhy109 29771288 PMC6041980

[IMAG.a.1235-b62] Ruigrok, A. N. V., Salimi-Khorshidi, G., Lai, M.-C., Baron-Cohen, S., Lombardo, M. V., Tait, R. J., & Suckling, J. (2014). A meta-analysis of sex differences in human brain structure. Neuroscience & Biobehavioral Reviews, 39, 34–50. 10.1016/j.neubiorev.2013.12.004 24374381 PMC3969295

[IMAG.a.1235-b63] Sabuncu, M. R., Desikan, R. S., Sepulcre, J., Yeo, B. T. T., Liu, H., Schmansky, N. J., Reuter, M., Weiner, M. W., Buckner, R. L., Sperling, R. A., Fischl, B., & Alzheimer’s Disease Neuroimaging Initiative. (2011). The dynamics of cortical and hippocampal atrophy in Alzheimer Disease. Archives of Neurology, 68(8), 1040–1048. 10.1001/archneurol.2011.167 21825241 PMC3248949

[IMAG.a.1235-b64] Sanchis-Segura, C., Aguirre, N., Cruz-Gómez, Á. J., Félix, S., & Forn, C. (2022). Beyond “sex prediction”: Estimating and interpreting multivariate sex differences and similarities in the brain. NeuroImage, 257, 119343. 10.1016/j.neuroimage.2022.119343 35654377

[IMAG.a.1235-b65] Sanchis-Segura, C., Ibañez-Gual, M. V., Adrián-Ventura, J., Aguirre, N., Gómez-Cruz, Á. J., Avila, C., & Forn, C. (2019). Sex differences in gray matter volume: How many and how large are they really? *Biology of Sex Differences*, 10(1), 32. 10.1186/s13293-019-0245-7 PMC6604149 31262342

[IMAG.a.1235-b66] Sanchis-Segura, C., Ibañez-Gual, M. V., Aguirre, N., Cruz-Gómez, Á. J., & Forn, C. (2020). Effects of different intracranial volume correction methods on univariate sex differences in grey matter volume and multivariate sex prediction. Scientific Reports, 10(1), Article 1. 10.1038/s41598-020-69361-9 PMC7395772 32737332

[IMAG.a.1235-b67] Sanfilipo, M. P., Benedict, R. H. B., Zivadinov, R., & Bakshi, R. (2004). Correction for intracranial volume in analysis of whole brain atrophy in multiple sclerosis: The proportion vs. residual method. NeuroImage, 22(4), 1732–1743. 10.1016/j.neuroimage.2004.03.037 15275929

[IMAG.a.1235-b68] Schwarz, C. G., Gunter, J. L., Wiste, H. J., Przybelski, S. A., Weigand, S. D., Ward, C. P., Senjem, M. L., Vemuri, P., Murray, M. E., Dickson, D. W., Parisi, J. E., Kantarci, K., Weiner, M. W., Petersen, R. C., & Jack, C. R. (2016). A large-scale comparison of cortical thickness and volume methods for measuring Alzheimer’s disease severity. NeuroImage: Clinical, 11, 802–812. 10.1016/j.nicl.2016.05.017 28050342 PMC5187496

[IMAG.a.1235-b69] Sepehrband, F., Lynch, K. M., Cabeen, R. P., Gonzalez-Zacarias, C., Zhao, L., D’Arcy, M., Kesselman, C., Herting, M. M., Dinov, I. D., Toga, A. W., & Clark, K. A. (2018). Neuroanatomical morphometric characterization of sex differences in youth using statistical learning. NeuroImage, 172, 217–227. 10.1016/j.neuroimage.2018.01.065 29414494 PMC5967879

[IMAG.a.1235-b70] Sled, J. G., Zijdenbos, A. P., & Evans, A. C. (1998). A nonparametric method for automatic correction of intensity nonuniformity in MRI data. IEEE Transactions on Medical Imaging, 17(1), 87–97. 10.1109/42.668698 9617910

[IMAG.a.1235-b71] Sowell, E. R., Peterson, B. S., Kan, E., Woods, R. P., Yoshii, J., Bansal, R., Xu, D., Zhu, H., Thompson, P. M., & Toga, A. W. (2007). Sex differences in cortical thickness mapped in 176 healthy individuals between 7 and 87 years of age. Cerebral Cortex, 17(7), 1550–1560. 10.1093/cercor/bhl066 16945978 PMC2329809

[IMAG.a.1235-b72] Sudlow, C., Gallacher, J., Allen, N., Beral, V., Burton, P., Danesh, J., Downey, P., Elliott, P., Green, J., Landray, M., Liu, B., Matthews, P., Ong, G., Pell, J., Silman, A., Young, A., Sprosen, T., Peakman, T., & Collins, R. (2015). UK Biobank: An open access resource for identifying the causes of a wide range of complex diseases of middle and old age. PLoS Medicine, 12(3), e1001779. 10.1371/journal.pmed.1001779 25826379 PMC4380465

[IMAG.a.1235-b73] Tamnes, C. K., Østby, Y., Fjell, A. M., Westlye, L. T., Due-Tønnessen, P., & Walhovd, K. B. (2010). Brain maturation in adolescence and young adulthood: Regional age-related changes in cortical thickness and white matter volume and microstructure. Cerebral Cortex, 20(3), 534–548. 10.1093/cercor/bhp118 19520764

[IMAG.a.1235-b74] van Eijk, L., Hansell, N. K., Strike, L. T., Couvy-Duchesne, B., de Zubicaray, G. I., Thompson, P. M., McMahon, K. L., Zietsch, B. P., & Wright, M. J. (2020). Region-specific sex differences in the hippocampus. NeuroImage, 215, 116781. 10.1016/j.neuroimage.2020.116781 32278894

[IMAG.a.1235-b75] Voevodskaya, O., Simmons, A., Nordenskjöld, R., Kullberg, J., Ahlström, H., Lind, L., Wahlund, L.-O., Larsson, E.-M., Westman, E., & Initiative, A. D. N. (2014). The effects of intracranial volume adjustment approaches on multiple regional MRI volumes in healthy aging and Alzheimer’s disease. Frontiers in Aging Neuroscience, 6, 264. 10.3389/fnagi.2014.00264 25339897 PMC4188138

[IMAG.a.1235-b76] Vuoksimaa, E., Panizzon, M. S., Franz, C. E., Fennema-Notestine, C., Hagler, D. J., Lyons, M. J., Dale, A. M., & Kremen, W. S. (2018). Brain structure mediates the association between height and cognitive ability. Brain Structure and Function, 223(7), 3487–3494. 10.1007/s00429-018-1675-4 29748873 PMC6425087

[IMAG.a.1235-b77] Wang, J., Hill-Jarrett, T., Buto, P., Pederson, A., Sims, K. D., Zimmerman, S. C., DeVost, M. A., Ferguson, E., Lacar, B., Yang, Y., Choi, M., Caunca, M. R., La Joie, R., Chen, R., Glymour, M. M., & Ackley, S. F. (2024). Comparison of approaches to control for intracranial volume in research on the association of brain volumes with cognitive outcomes. Human Brain Mapping, 45(4), e26633. 10.1002/hbm.26633 38433682 PMC10910271

[IMAG.a.1235-b78] Wang, L., Shen, H., Tang, F., Zang, Y., & Hu, D. (2012). Combined structural and resting-state functional MRI analysis of sexual dimorphism in the young adult human brain: An MVPA approach. NeuroImage, 61(4), 931–940. 10.1016/j.neuroimage.2012.03.080 22498657

[IMAG.a.1235-b79] Wen, Z., Hammoud, M. Z., Siegel, C. E., Laska, E. M., Abu-Amara, D., Etkin, A., Milad, M. R., & Marmar, C. R. (2025). Neuroimaging-based variability in subtyping biomarkers for psychiatric heterogeneity. Molecular Psychiatry, 30(5), 1966–1975. 10.1038/s41380-024-02807-y 39511450 PMC12015113

[IMAG.a.1235-b80] Westman, E., Aguilar, C., Muehlboeck, J.-S., & Simmons, A. (2013). Regional magnetic resonance imaging measures for multivariate analysis in Alzheimer’s disease and mild cognitive impairment. Brain Topography, 26(1), 9–23. 10.1007/s10548-012-0246-x 22890700 PMC3536978

[IMAG.a.1235-b81] Williams, C. M., Peyre, H., Toro, R., & Ramus, F. (2021). Neuroanatomical norms in the UK Biobank: The impact of allometric scaling, sex, and age. Human Brain Mapping, 42(14), 4623–4642. 10.1002/hbm.25572 34268815 PMC8410561

[IMAG.a.1235-b82] Williams, C. M., Peyre, H., Toro, R., & Ramus, F. (2022). Comparing brain asymmetries independently of brain size. NeuroImage, 254, 119118. 10.1016/j.neuroimage.2022.119118 35318151

[IMAG.a.1235-b83] Williams, M. E., Fennema‐Notestine, C., Bell, T. R., Lin, S., Glatt, S. J., Kremen, W. S., & Elman, J. A. (2025). Neuroimaging predictors of cognitive resilience against Alzheimer’s disease pathology. Annals of Neurology, 97(6), 1038–1050. 10.1002/ana.27186 39891430 PMC12081995

[IMAG.a.1235-b84] Worsley, K. j., Andermann, M., Koulis, T., MacDonald, D., & Evans, A. c. (1999). Detecting changes in nonisotropic images. Human Brain Mapping, 8(2–3), 98–101. 10.1002/(SICI)1097-0193(1999)8:2/3<98::AID-HBM5>3.0.CO;2-F 10524599 PMC6873343

[IMAG.a.1235-b85] Wuestefeld, A., Xie, L., McGrew, E., Pichet‐Binette, A., Spotorno, N., van Westen, D., Mattsson‐Carlgren, N., Yushkevich, P. A., Das, S. R., Wolk, D. A., & Wisse, L. E. M. (2025). Tau, atrophy, and domain‐specific cognitive impairment in typical Alzheimer’s disease. Alzheimer’s & Dementia, 21(7), e70511. 10.1002/alz.70511 PMC12290488 40709494

[IMAG.a.1235-b86] Yau, Y., Zeighami, Y., Baker, T. E., Larcher, K., Vainik, U., Dadar, M., Fonov, V. S., Hagmann, P., Griffa, A., Mišić, B., Collins, D. L., & Dagher, A. (2018). Network connectivity determines cortical thinning in early Parkinson’s disease progression. Nature Communications, 9(1), 12. 10.1038/s41467-017-02416-0 PMC5750227 29295991

[IMAG.a.1235-b87] Young, A. L., Marinescu, R. V., Oxtoby, N. P., Bocchetta, M., Yong, K., Firth, N. C., Cash, D. M., Thomas, D. L., Dick, K. M., Cardoso, J., van Swieten, J., Borroni, B., Galimberti, D., Masellis, M., Tartaglia, M. C., Rowe, J. B., Graff, C., Tagliavini, F., Frisoni, G. B., … Alexander, D. C. (2018). Uncovering the heterogeneity and temporal complexity of neurodegenerative diseases with Subtype and Stage Inference. Nature Communications, 9(1), Article 1. 10.1038/s41467-018-05892-0 PMC6189176 30323170

[IMAG.a.1235-b88] Zeighami, Y., & Evans, A. C. (2021). Association vs. prediction: The impact of cortical surface smoothing and parcellation on brain age. Frontiers in Big Data, 4, 637724. 10.3389/fdata.2021.637724 34027399 PMC8131952

[IMAG.a.1235-b89] Zeighami, Y., Fereshtehnejad, S.-M., Dadar, M., Collins, D. L., Postuma, R. B., & Dagher, A. (2019). Assessment of a prognostic MRI biomarker in early de novo Parkinson’s disease. NeuroImage: Clinical, 24, 101986. 10.1016/j.nicl.2019.101986 31514113 PMC6742805

[IMAG.a.1235-b90] Zhao, L., Boucher, M., Rosa-Neto, P., & Evans, A. C. (2013). Impact of scale space search on age- and gender-related changes in MRI-based cortical morphometry. Human Brain Mapping, 34(9), 2113–2128. 10.1002/hbm.22050 22422546 PMC6870203

[IMAG.a.1235-b91] Zhou, Q., Goryawala, M., Cabrerizo, M., Barker, W., Duara, R., & Adjouadi, M. (2014). Significance of normalization on anatomical MRI measures in predicting Alzheimer’s disease. The Scientific World Journal, 2014, 541802. 10.1155/2014/541802 24550710 PMC3914452

