# Supplementary material for: Intracranial volume: To adjust or not to adjust? It is not a matter of if, but how
Source: Imaging Neurosci (Camb). 2026 May 22;4:IMAG.a.1235. doi: 10.1162/IMAG.a.1235 (PMC13206449; doi:10.1162/IMAG.a.1235)
Supplement: Supplementary Material [file IMAG.a.1235_supp.pdf]

Intracranial Volume: To Adjust or Not to Adjust? It's Not a Matter of If, but How

Supplementary Material

Brzezinski-Rittner, et.al.

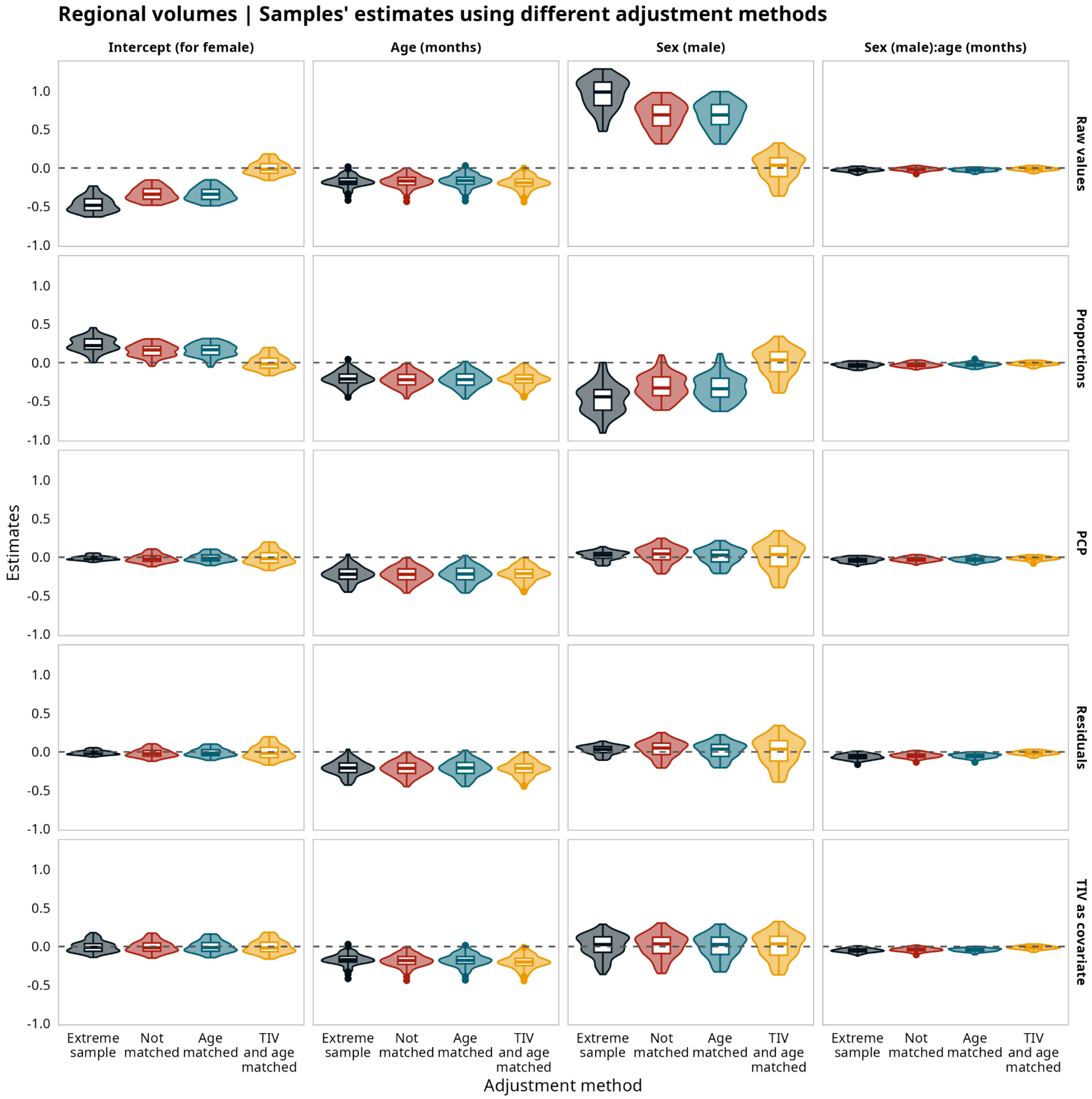

Supplementary Figure 1. Regional volumes. Model's estimates for regional volumes for all the samples and all the adjustment methods.

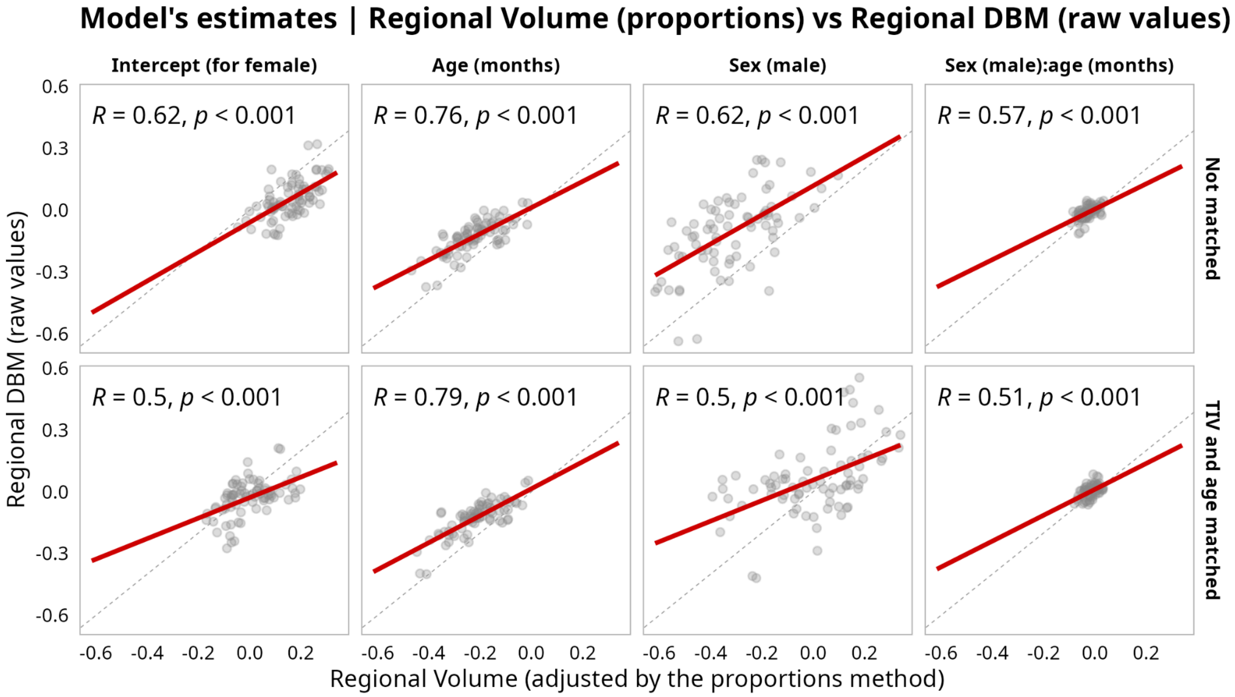

Supplementary Figure 2. Comparison between the model's estimates for volumetric data adjusted using the proportions method versus the estimates for regional deformation based morphometry data without adjustment.

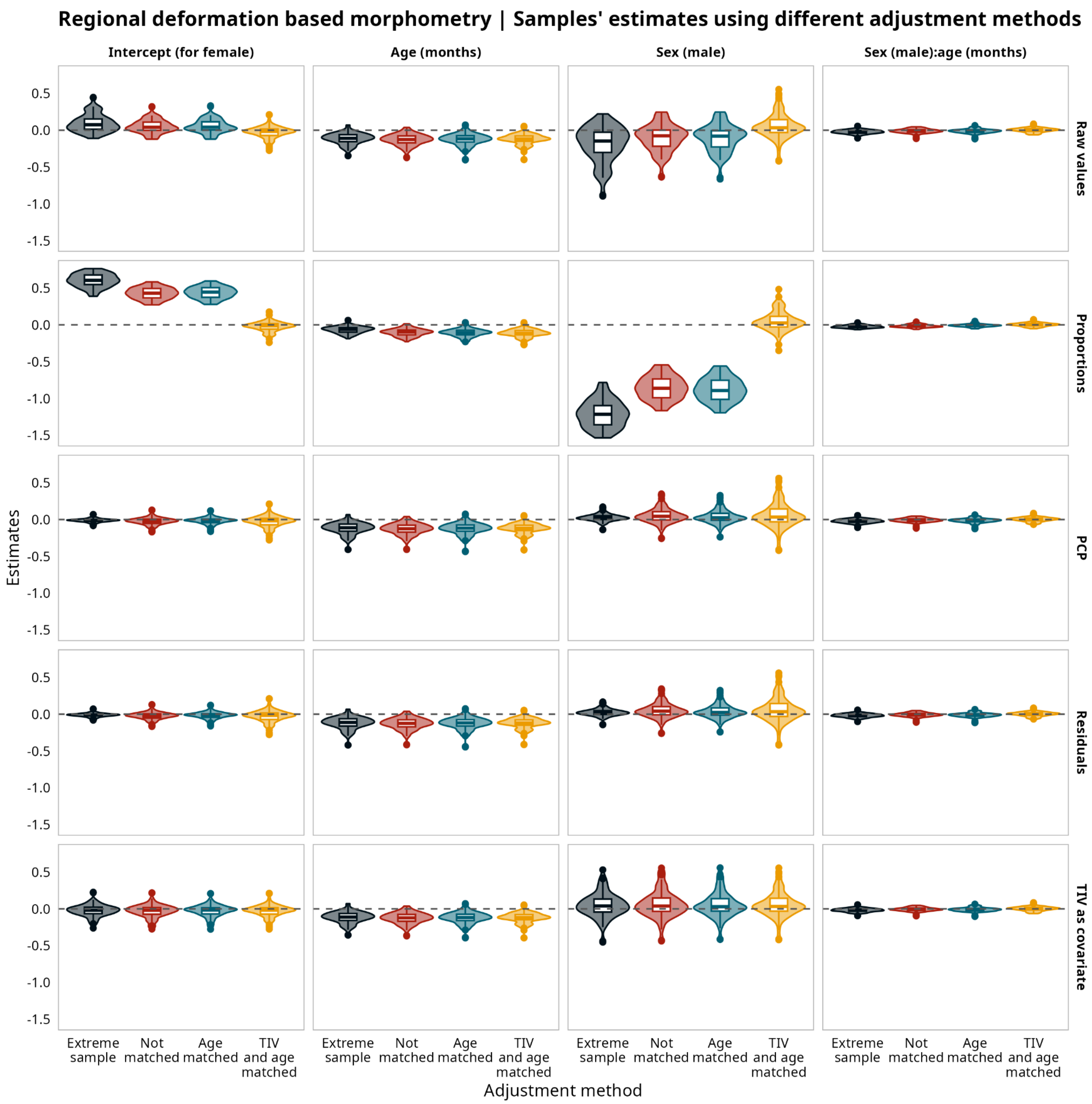

Supplementary Figure 3. Regional deformation based morphometry (DBM). Model's estimates for regional DBM for all the samples and all the adjustment methods.

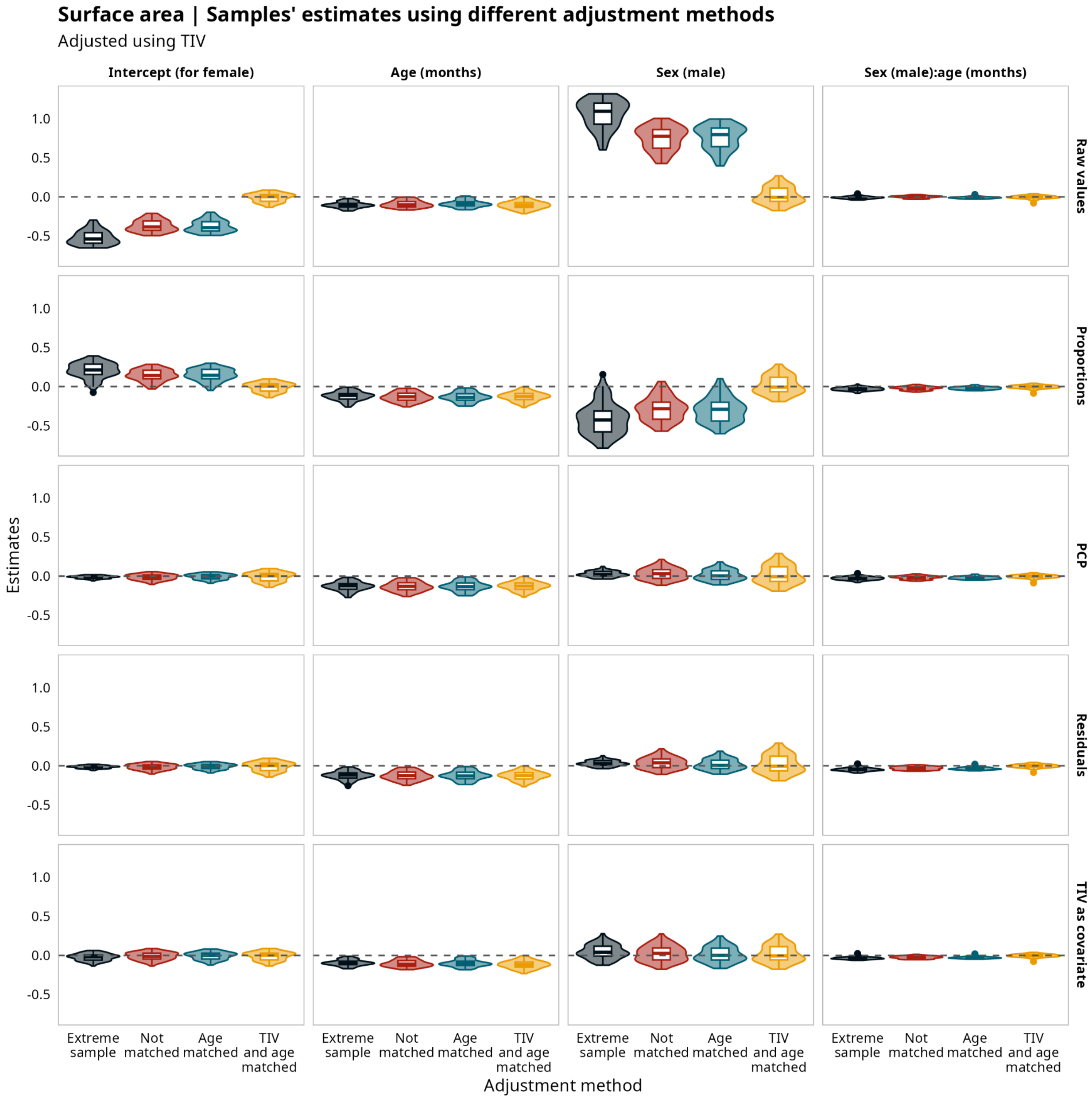

Supplementary Figure 4. Regional surface area. Model's estimates for regional surface area adjusted using total intracranial volume (TIV) for all the samples and all the adjustment methods.

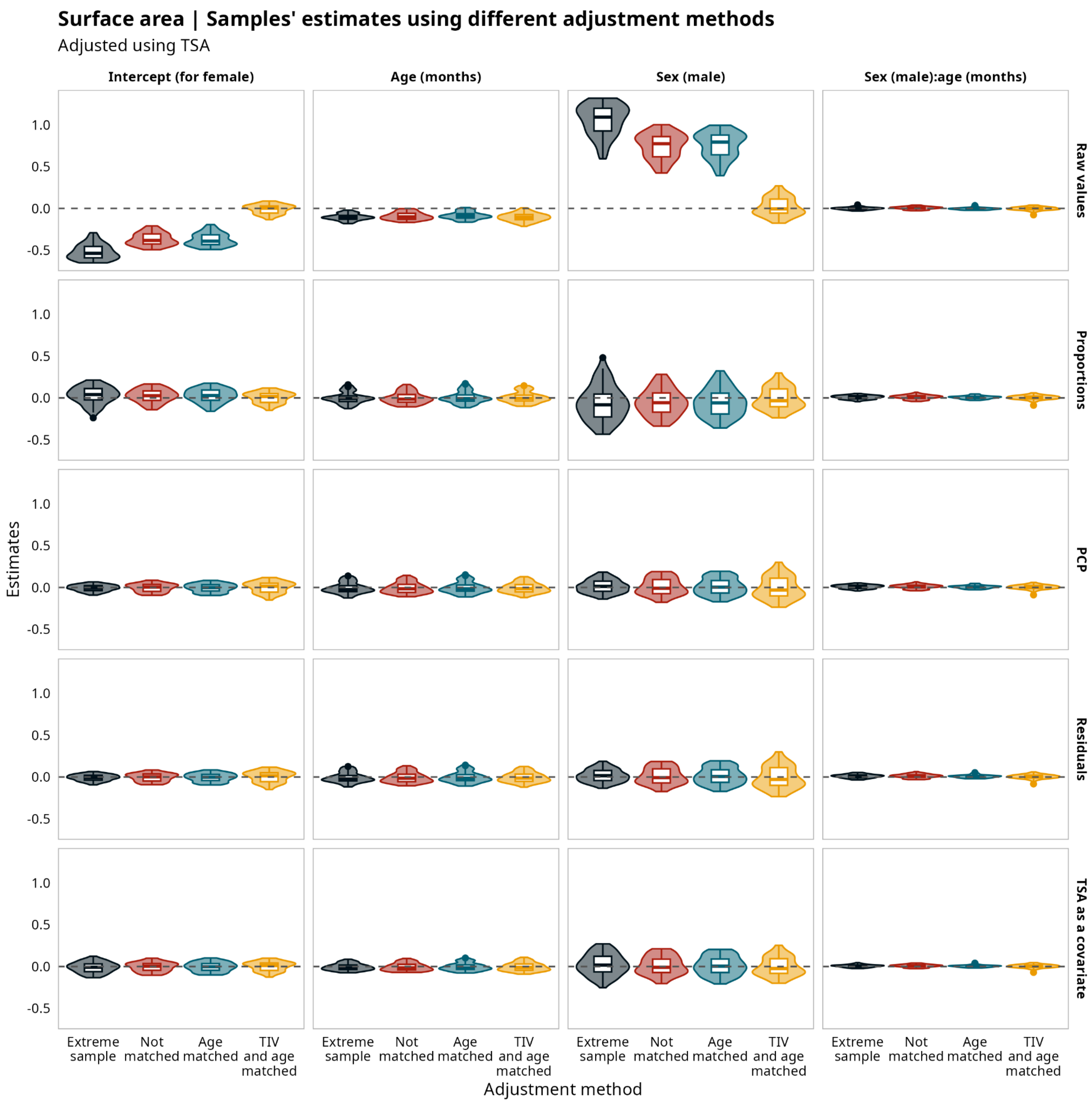

Supplementary Figure 5. Regional Surface Area. Model's estimates for regional surface area corrected using total surface area (TSA) for all the samples and all the adjustment methods.

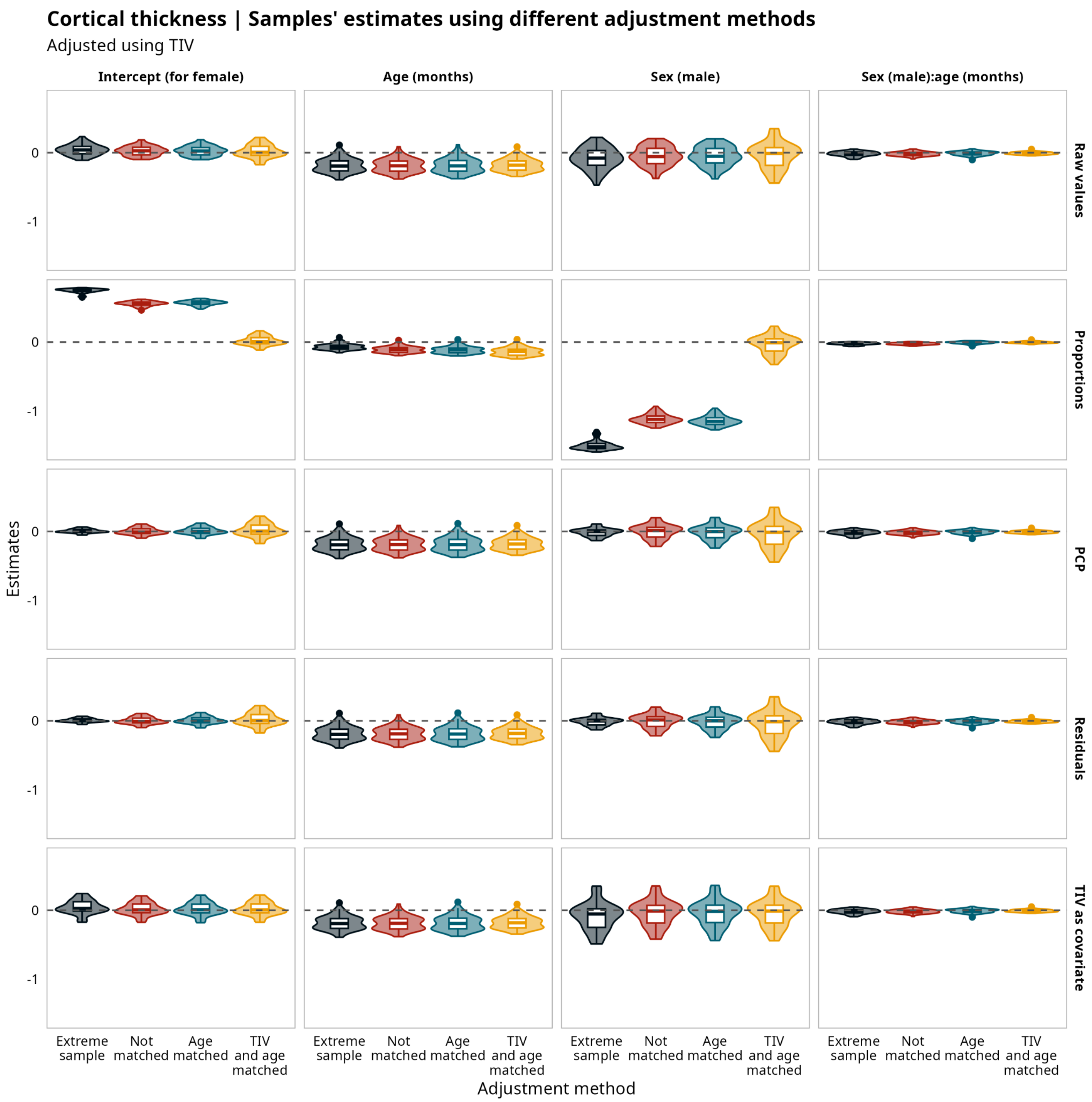

Supplementary Figure 6. Regional Cortical Thickness. Model's estimates for regional cortical thickness adjusted using total intracranial volume (TIV) for all the samples and all the adjustment methods.

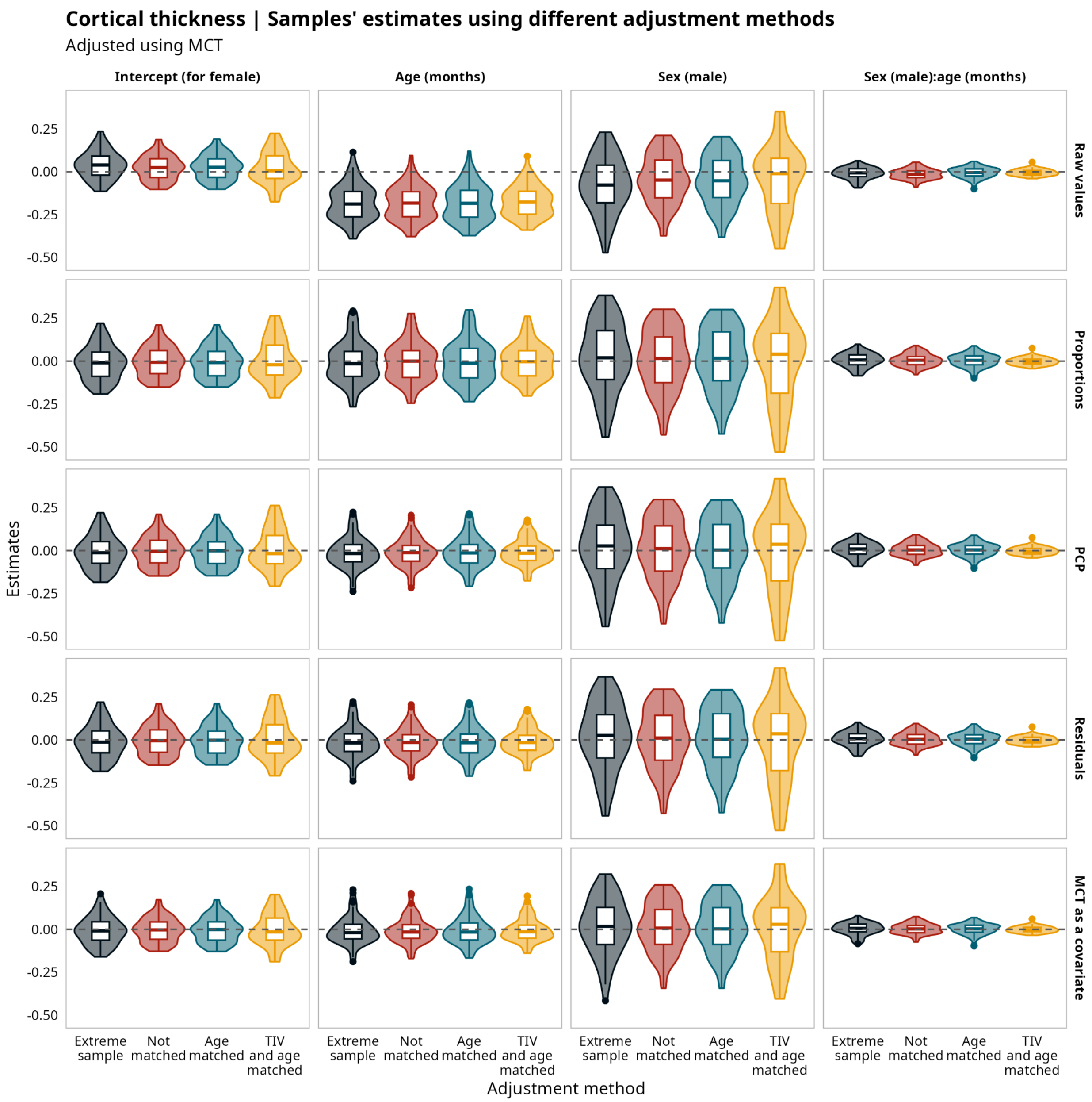

Supplementary Figure 7. Regional Cortical Thickness. Model's estimates for regional cortical thickness adjusted using mean cortical thickness (MCT) for all the samples and all the adjustment methods.

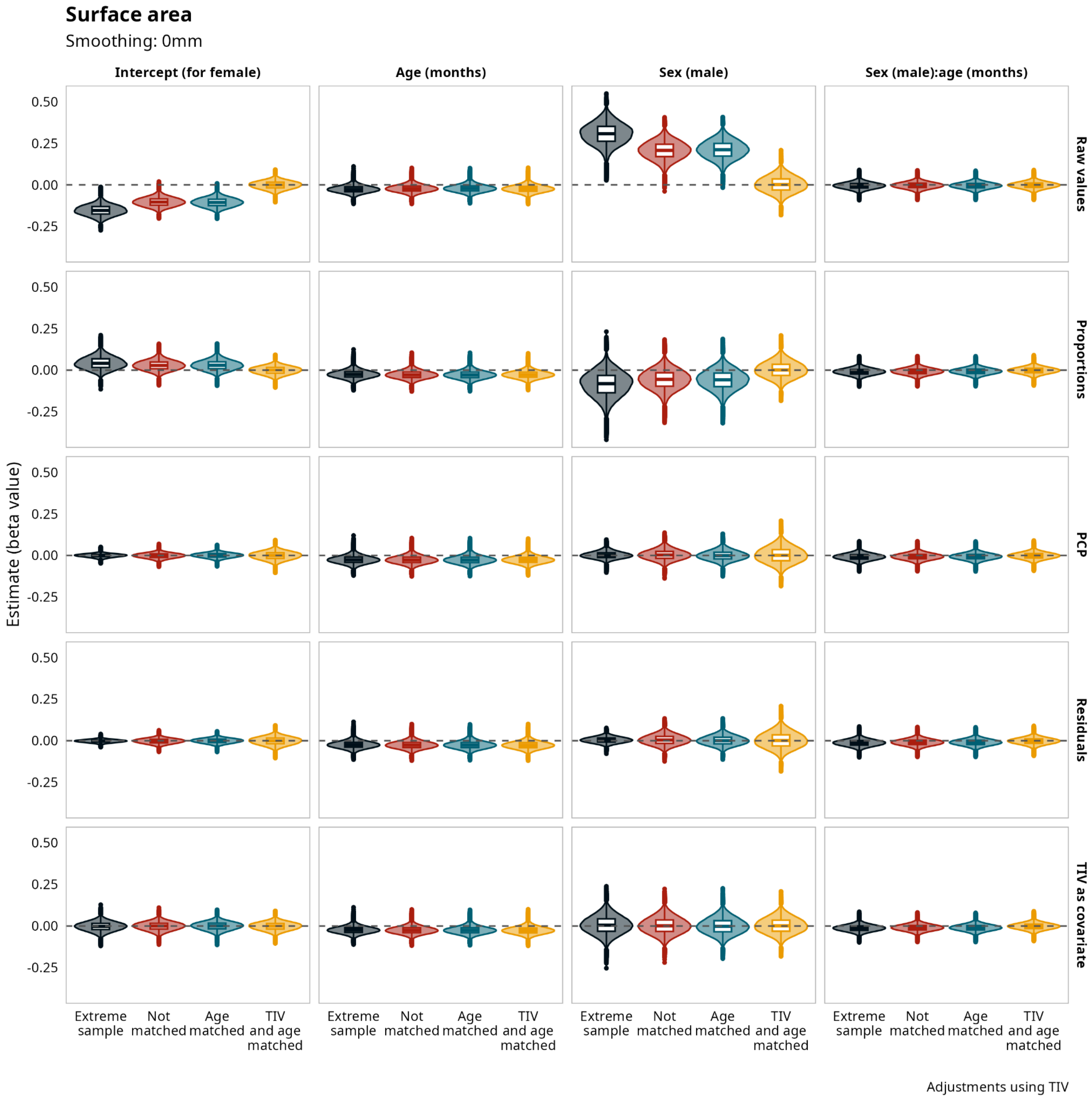

Supplementary Figure 8. Vertexwise Surface Area. Model's estimates for vertexwise surface area without smoothing corrected using total intracranial volume (TIV) for all the samples and all the adjustment methods.

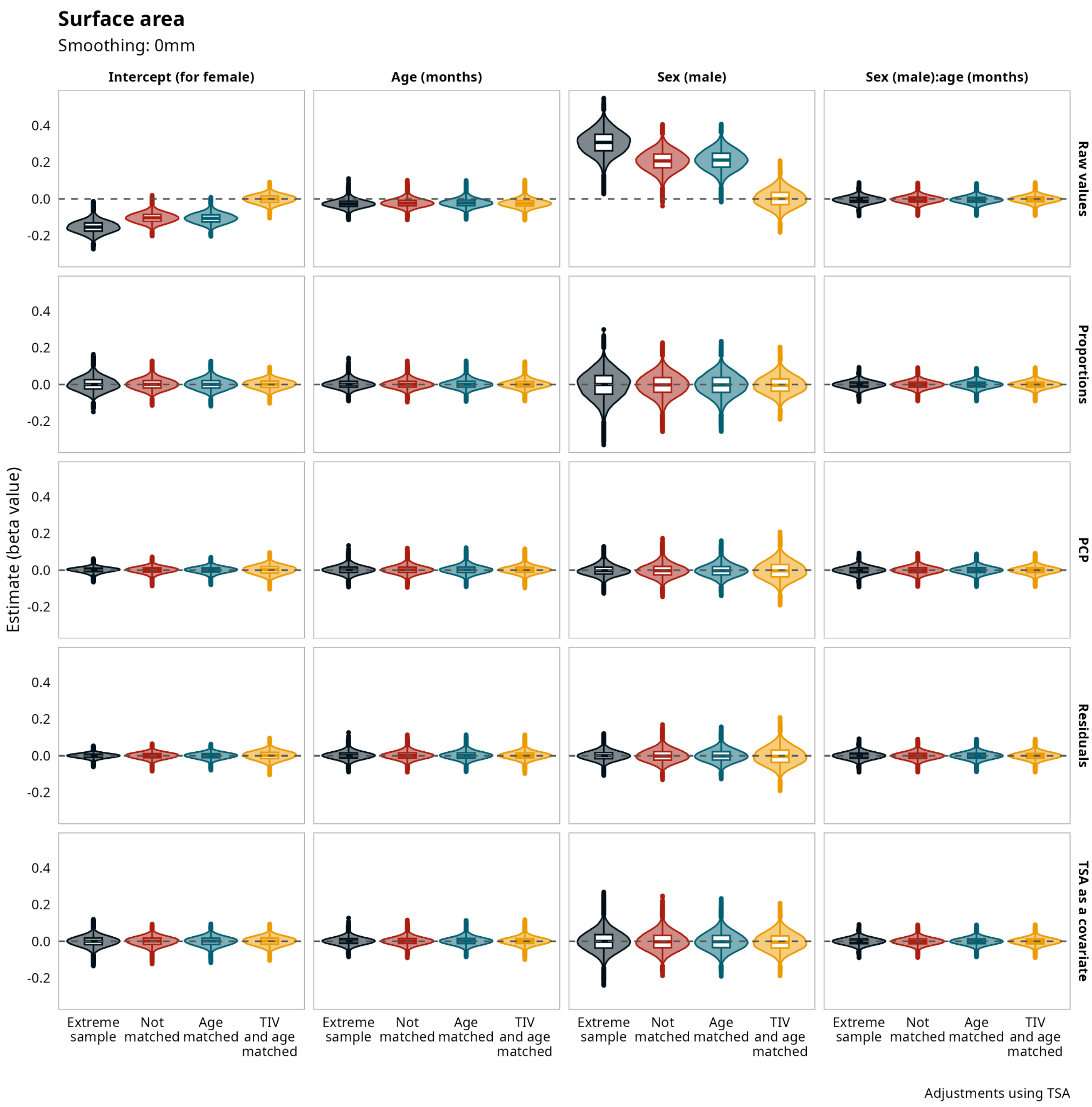

Supplementary Figure 9. Vertexwise Surface Area. Model's estimates for vertexwise surface area without smoothing corrected using total surface area (TSA) for all the samples and all the adjustment methods.

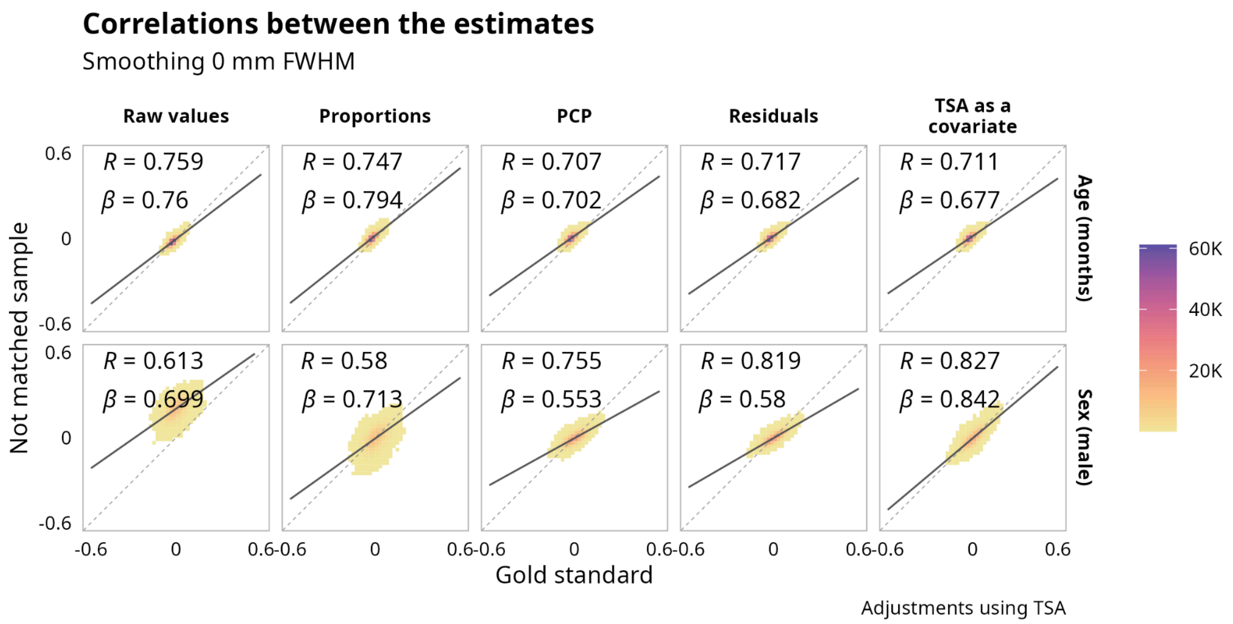

Supplementary Figure 10. Vertexwise Surface Area. Correlations between estimates for the matched sample without adjustment and the estimates after using different adjustment methods with total surface area (TSA) and the not matched sample, with data without smoothing.

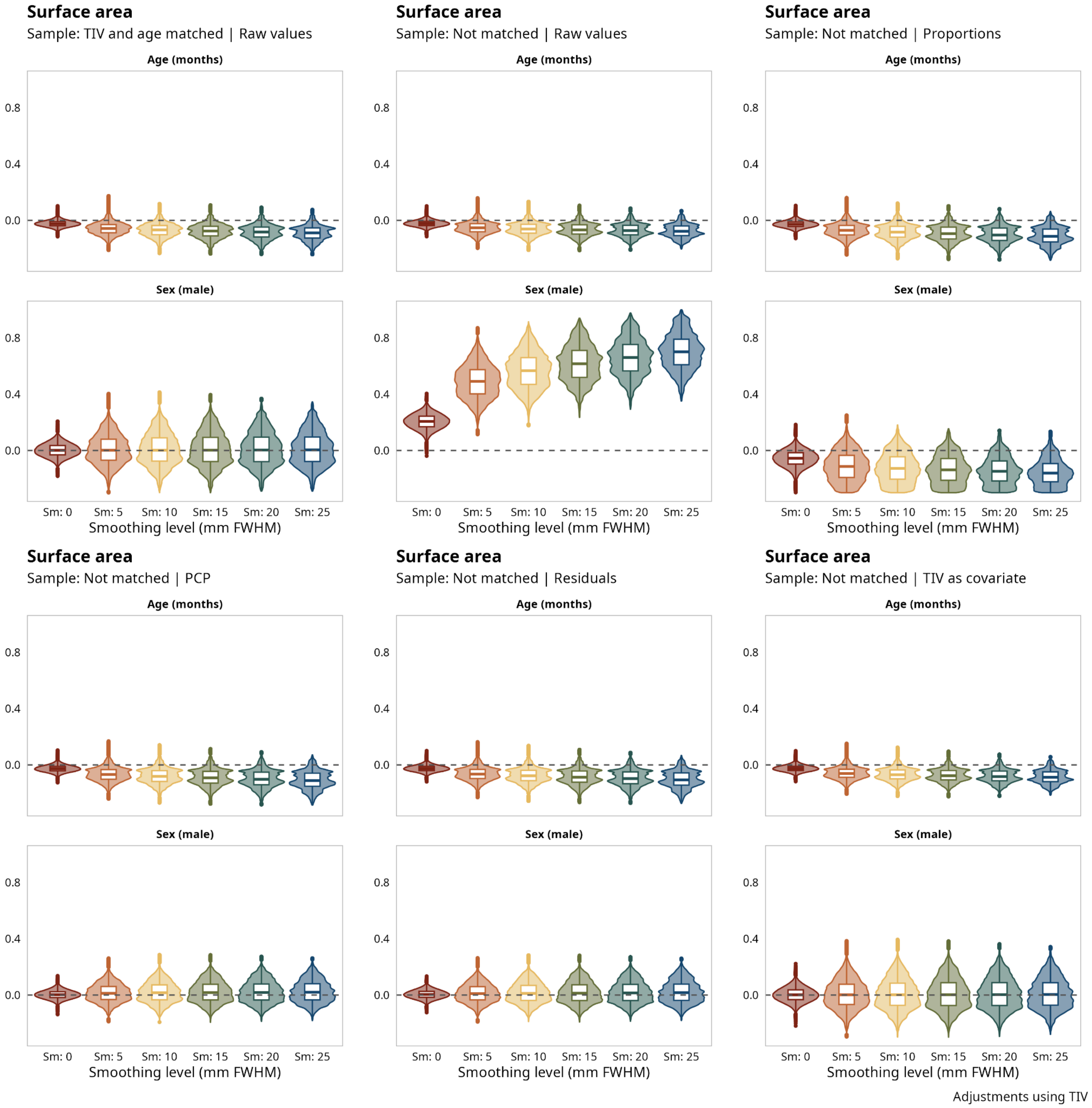

Supplementary Figure 11. Vertexwise Surface Area. Effect of different levels of smoothing on age and sex estimation, using the raw values in the matched and not matched samples and adjusting using total intracranial volume (TIV) in the not matched sample.

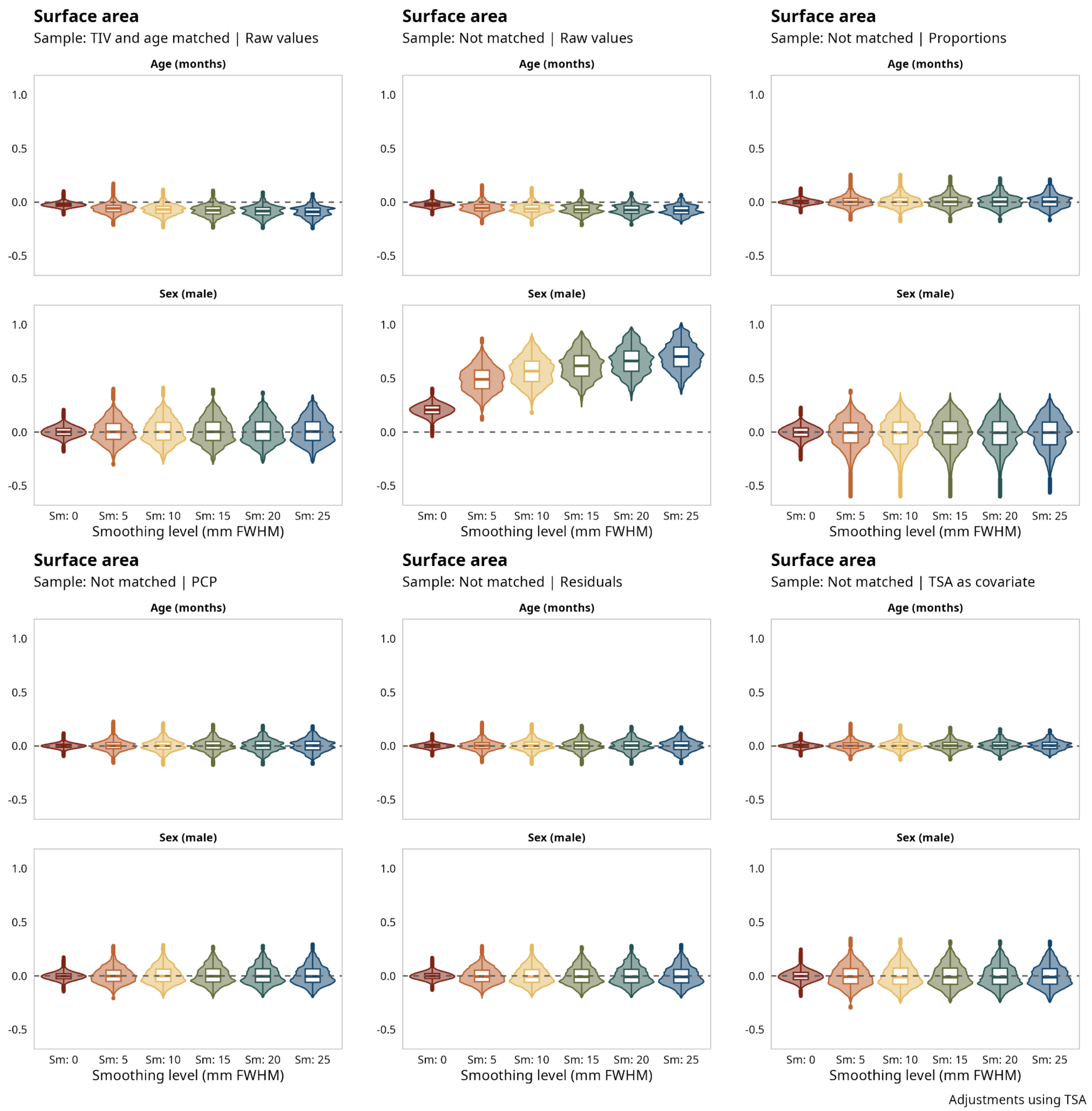

Supplementary Figure 12. Vertexwise Surface Area. Effect of different levels of smoothing on age and sex estimation, using the raw values in the matched and not matched samples and adjusting using total surface area (TSA) in the not matched sample.

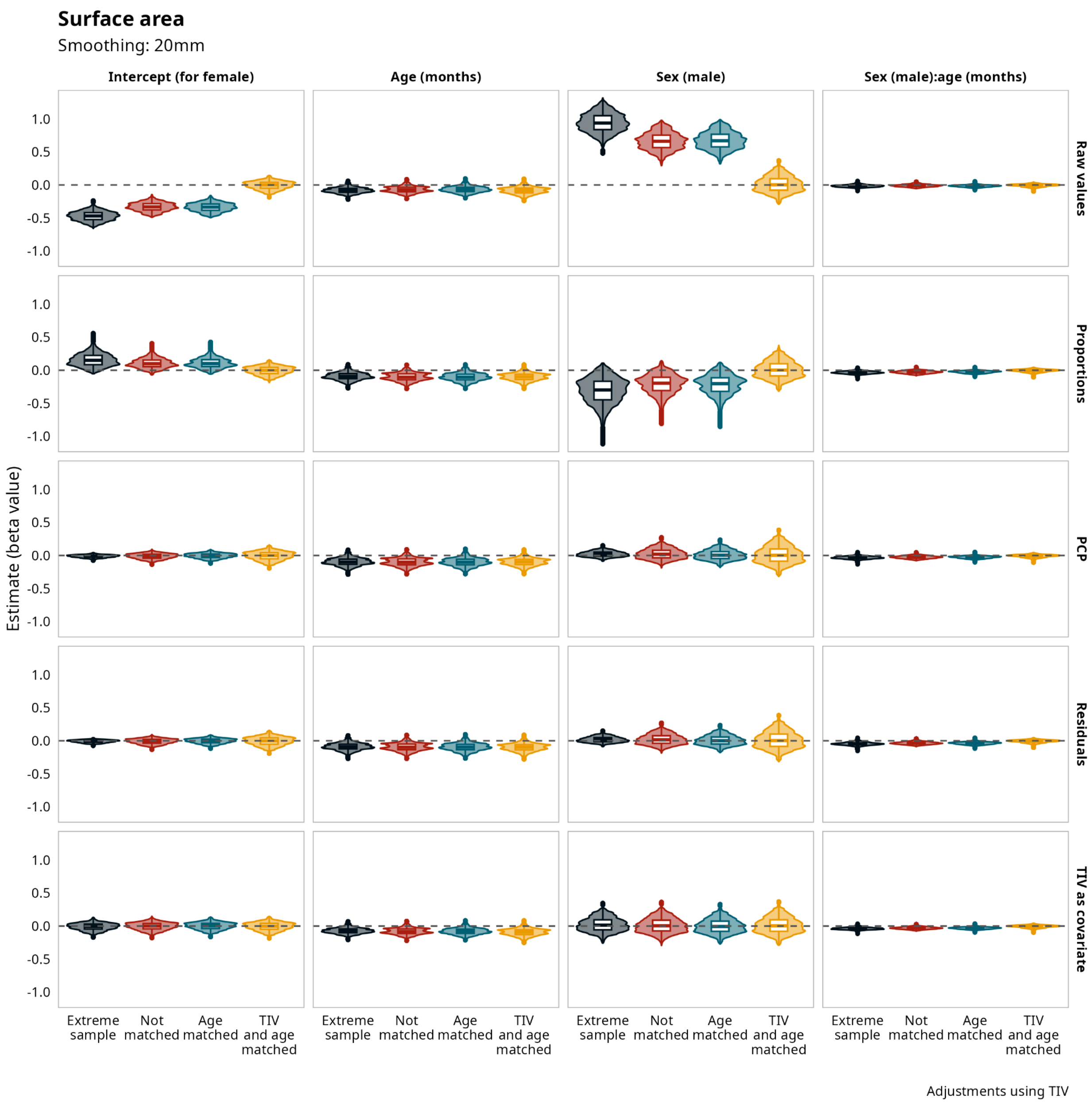

Supplementary Figure 13. Vertexwise Surface Area. Model's estimates for vertexwise surface area smoothed 20mm FWHM, corrected using total intracranial volume (TIV) for all the samples and all the adjustment methods.

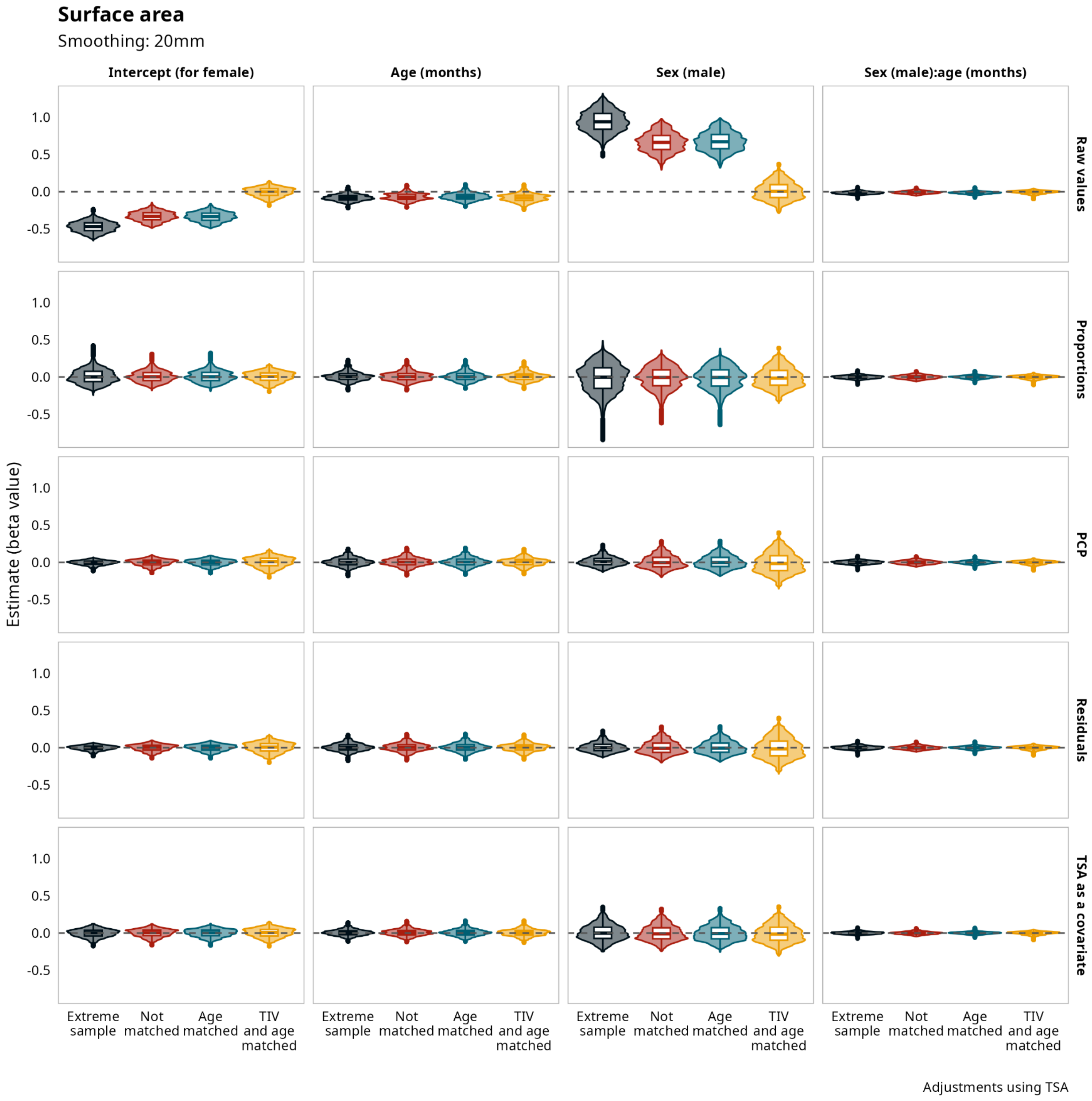

Supplementary Figure 14. Vertexwise Surface Area. Model's estimates for vertexwise surface area smoothed 20mm FWHM, corrected using total surface area (TSA) for all the samples and all the adjustment methods.

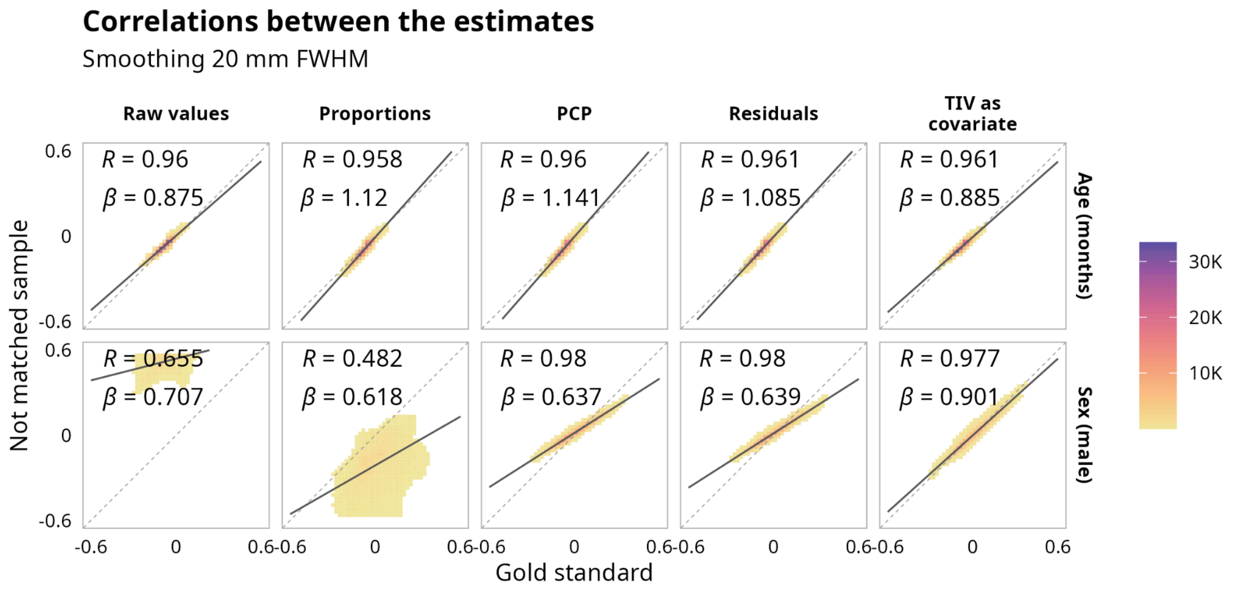

Supplementary Figure 15. Vertexwise Surface Area. Correlations between estimates for the matched sample without adjustment and the estimates after using different adjustment methods with total intracranial volume (TIV) and the not matched sample, with data smoothed 20mm FWHM.

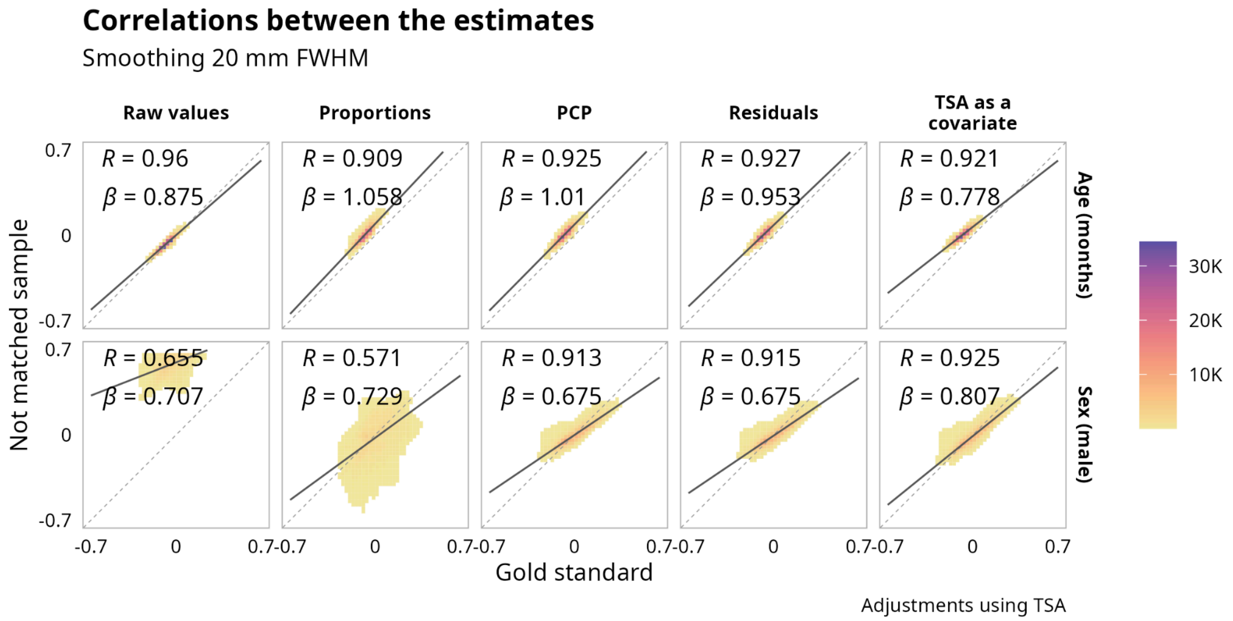

Supplementary Figure 16. Vertexwise Surface Area. Correlations between estimates for the matched sample without adjustment and the estimates after using different adjustment methods with total surface area (TSA) and the not matched sample, with data smoothed 20mm FWHM.

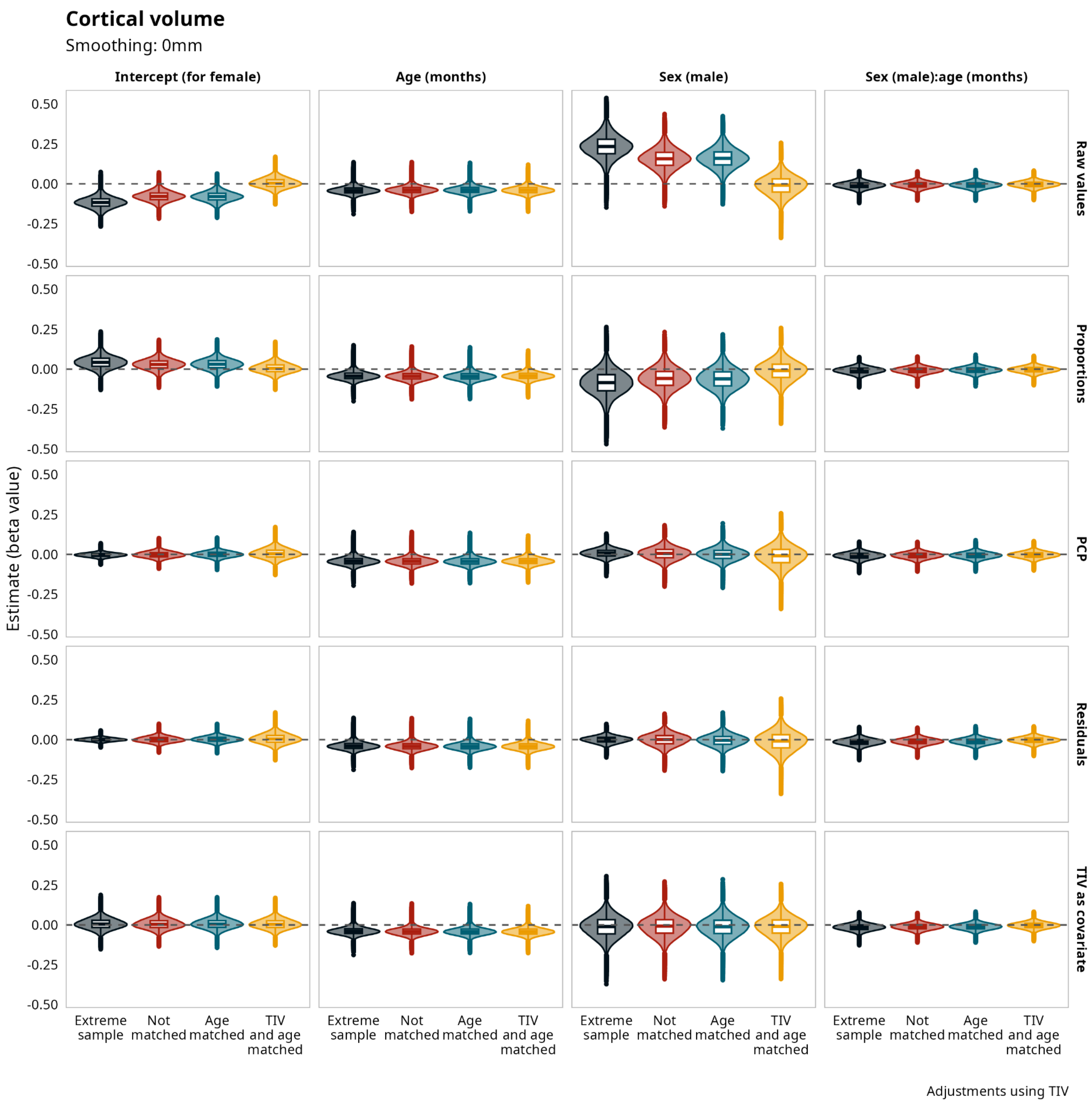

Supplementary Figure 17. Vertexwise Cortical Volume. Model's estimates for vertexwise cortical volume without smoothing corrected using total intracranial volume (TIV) for all the samples and all the adjustment methods.

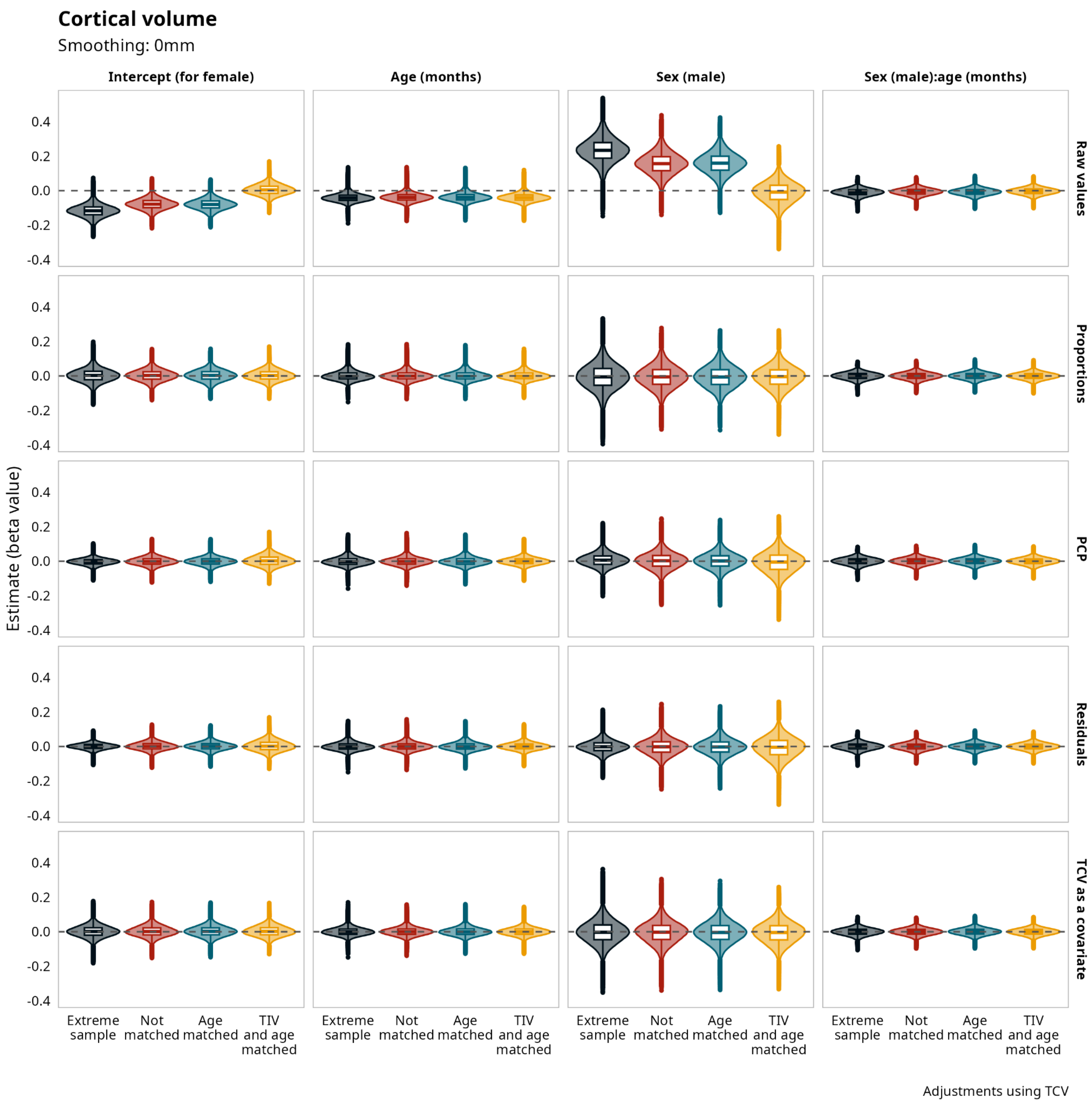

Supplementary Figure 18. Vertexwise Cortical Volume. Model's estimates for vertexwise cortical volume without smoothing corrected using total cortical volume (TCV) for all the samples and all the adjustment methods.

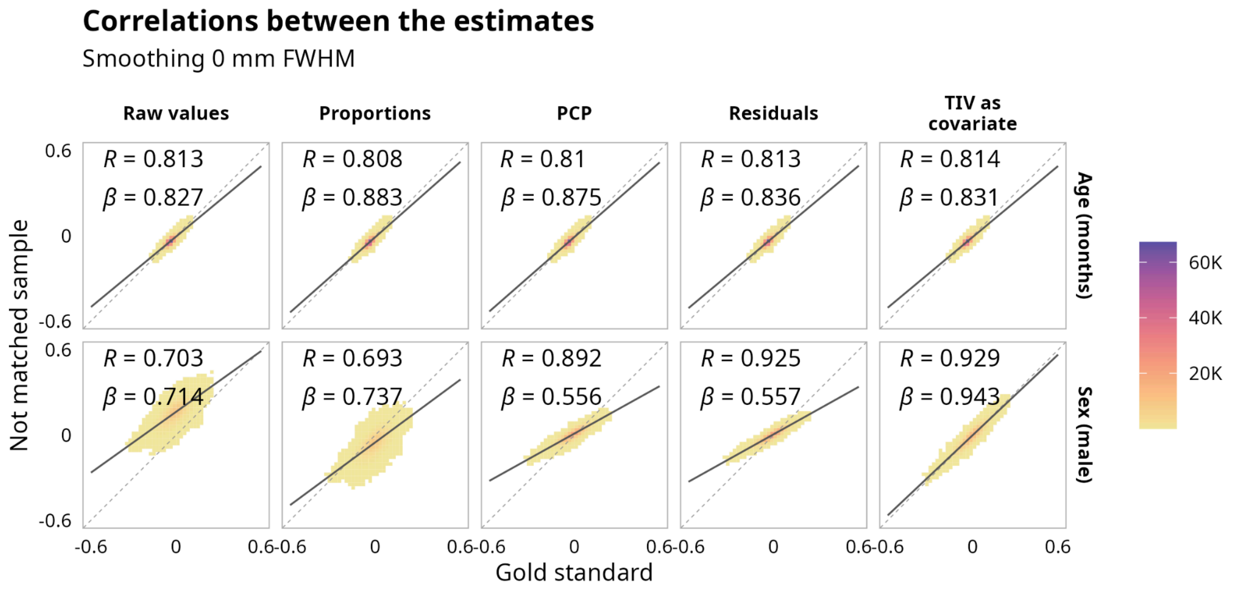

Supplementary Figure 19. Vertexwise Cortical Volume. Correlations between estimates for the matched sample without adjustment and the estimates after using different adjustment methods with total intracranial volume (TIV) and the not matched sample, with data without smoothing.

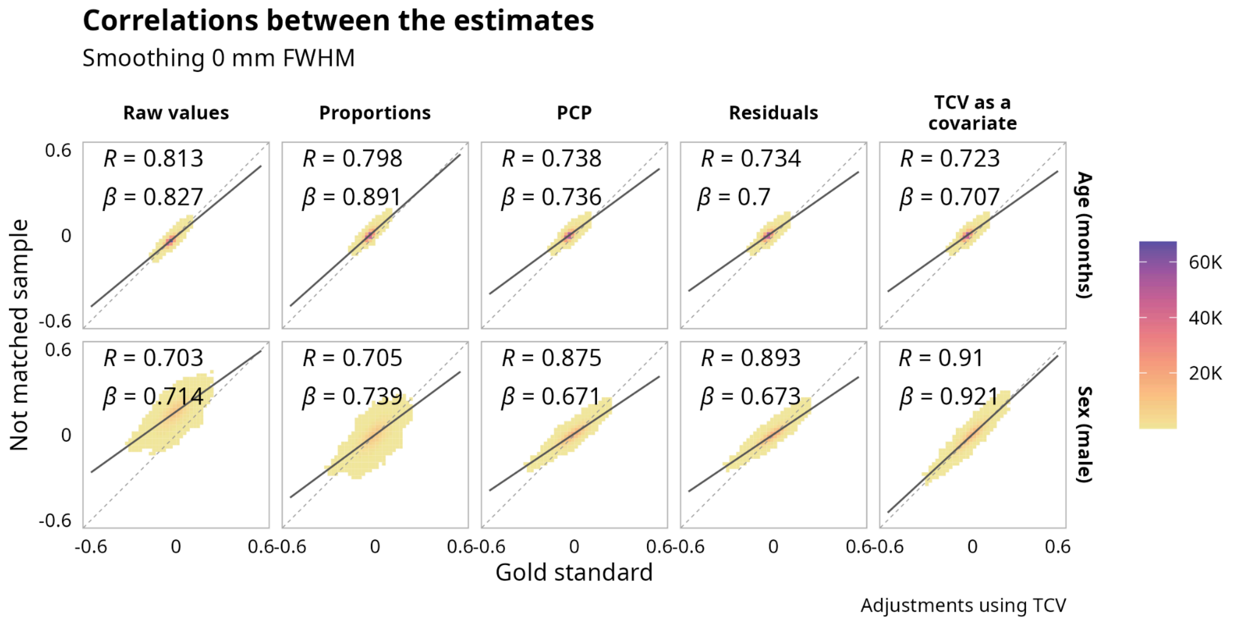

Supplementary Figure 20. Vertexwise Cortical Volume. Correlations between estimates for the matched sample without adjustment and the estimates after using different adjustment methods with total cortical volume (TCV) and the not matched sample, with data without smoothing.

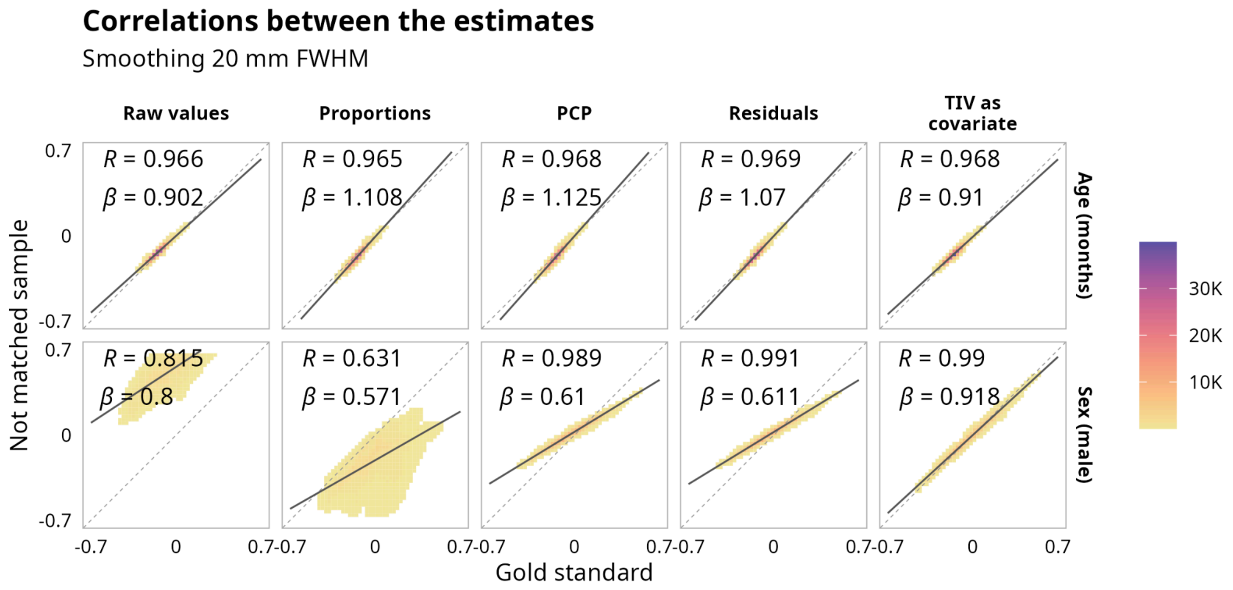

Supplementary Figure 21. Vertexwise Cortical Volume. Correlations between estimates for the matched sample without adjustment and the estimates after using different adjustment methods with total intracranial volume (TIV) and the not matched sample, with data smoothed at 20mm FWHM.

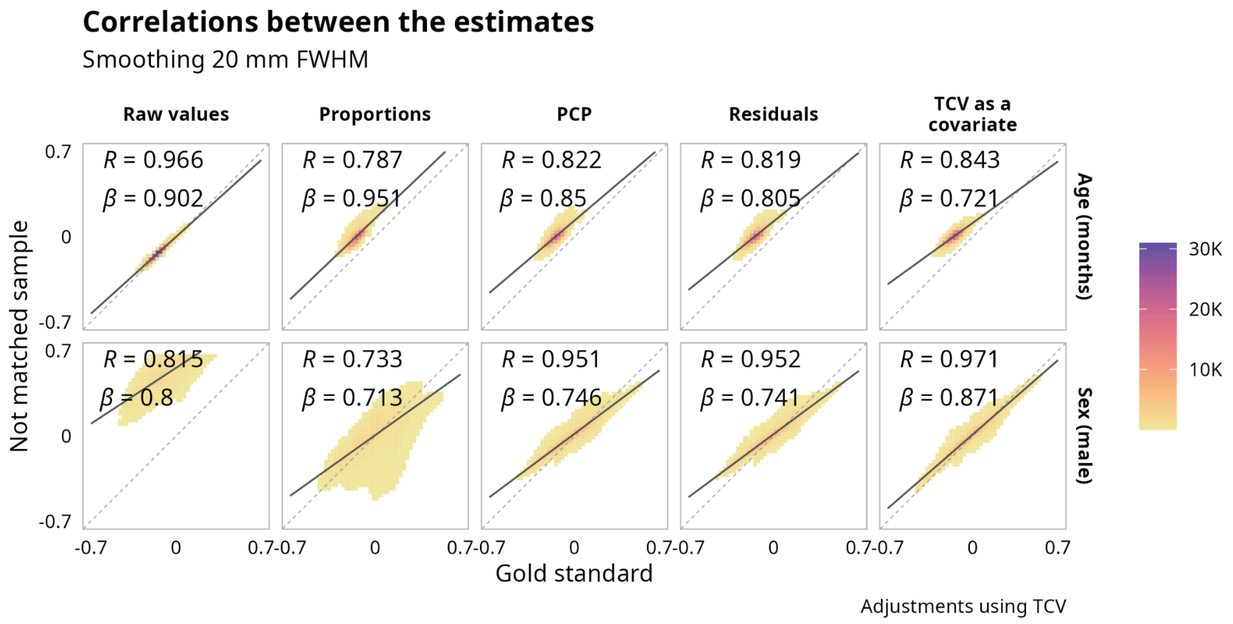

Supplementary Figure 22. Vertexwise Cortical Volume. Correlations between estimates for the matched sample without adjustment and the estimates after using different adjustment methods with total cortical volume (TCV) and the not matched sample, with data smoothed at 20mm FWHM.

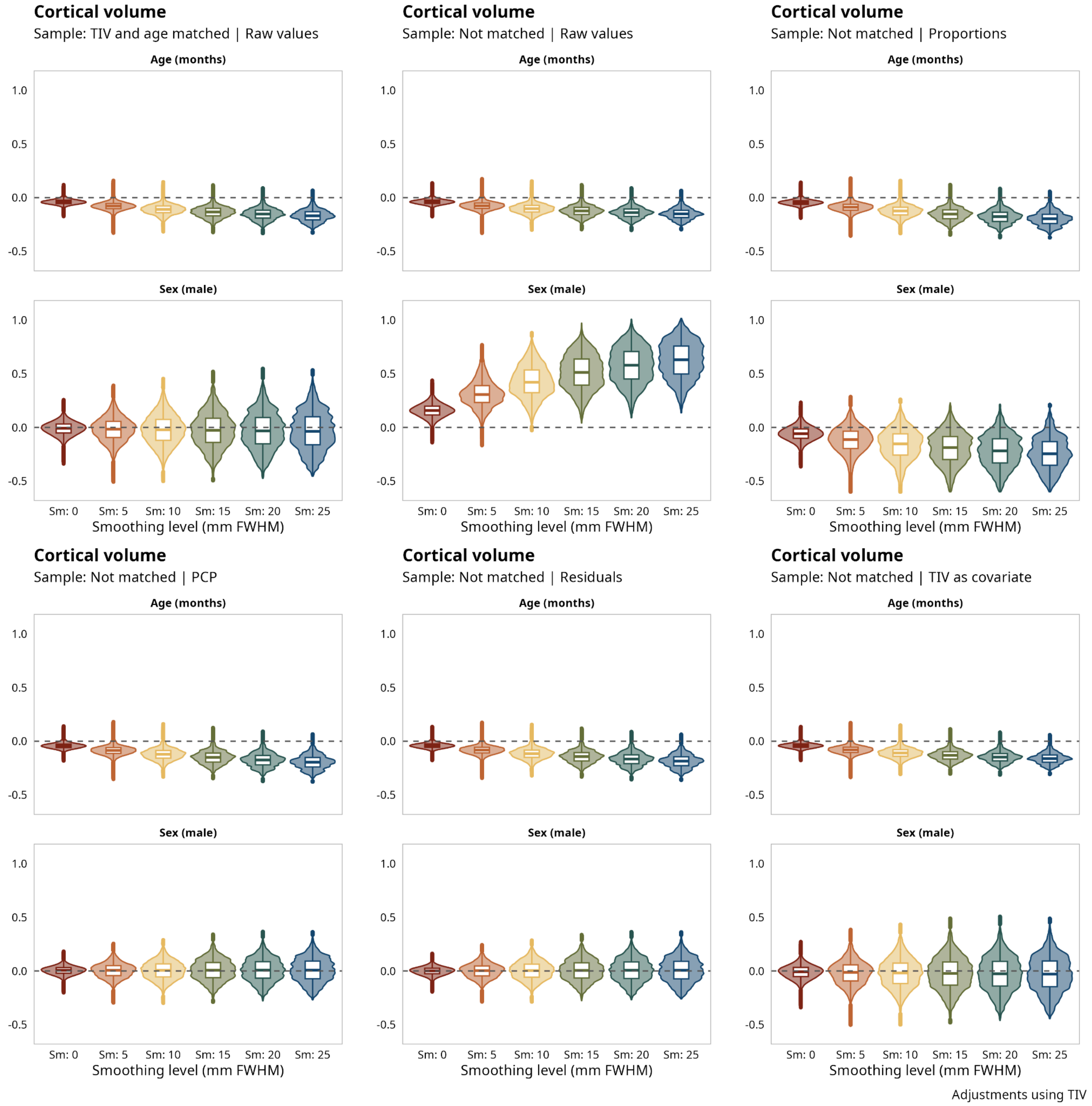

Supplementary Figure 23. Vertexwise Cortical Volume. Effect of different levels of smoothing on age and sex estimation, using the raw values in the matched and not matched samples and adjusting using total intracranial volume (TIV) in the not matched sample.

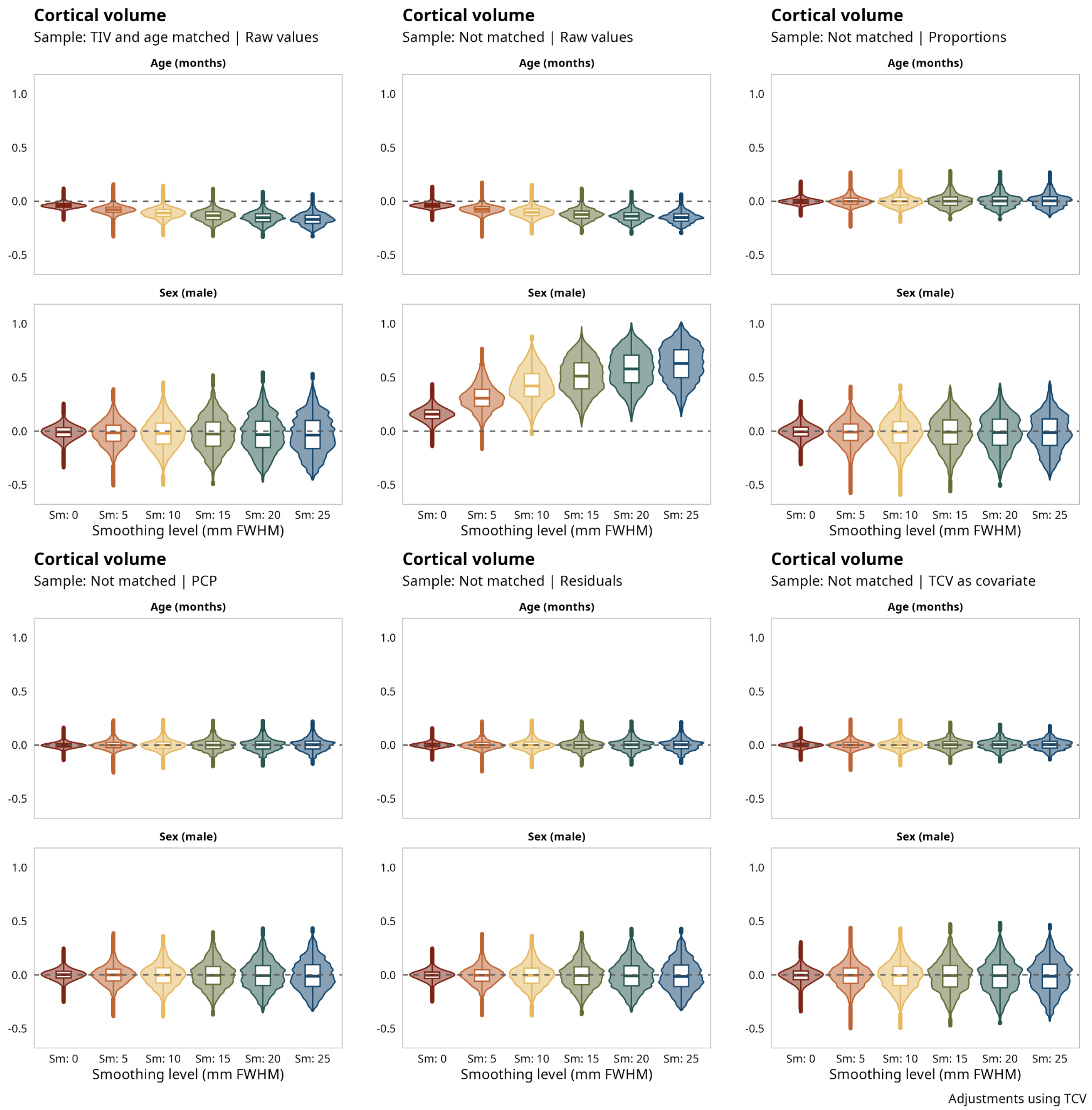

Supplementary Figure 24. Vertexwise Cortical Volume. Effect of different levels of smoothing on age and sex estimation, using the raw values in the matched and not matched samples and adjusting using total cortical volume (TCV) in the not matched sample.

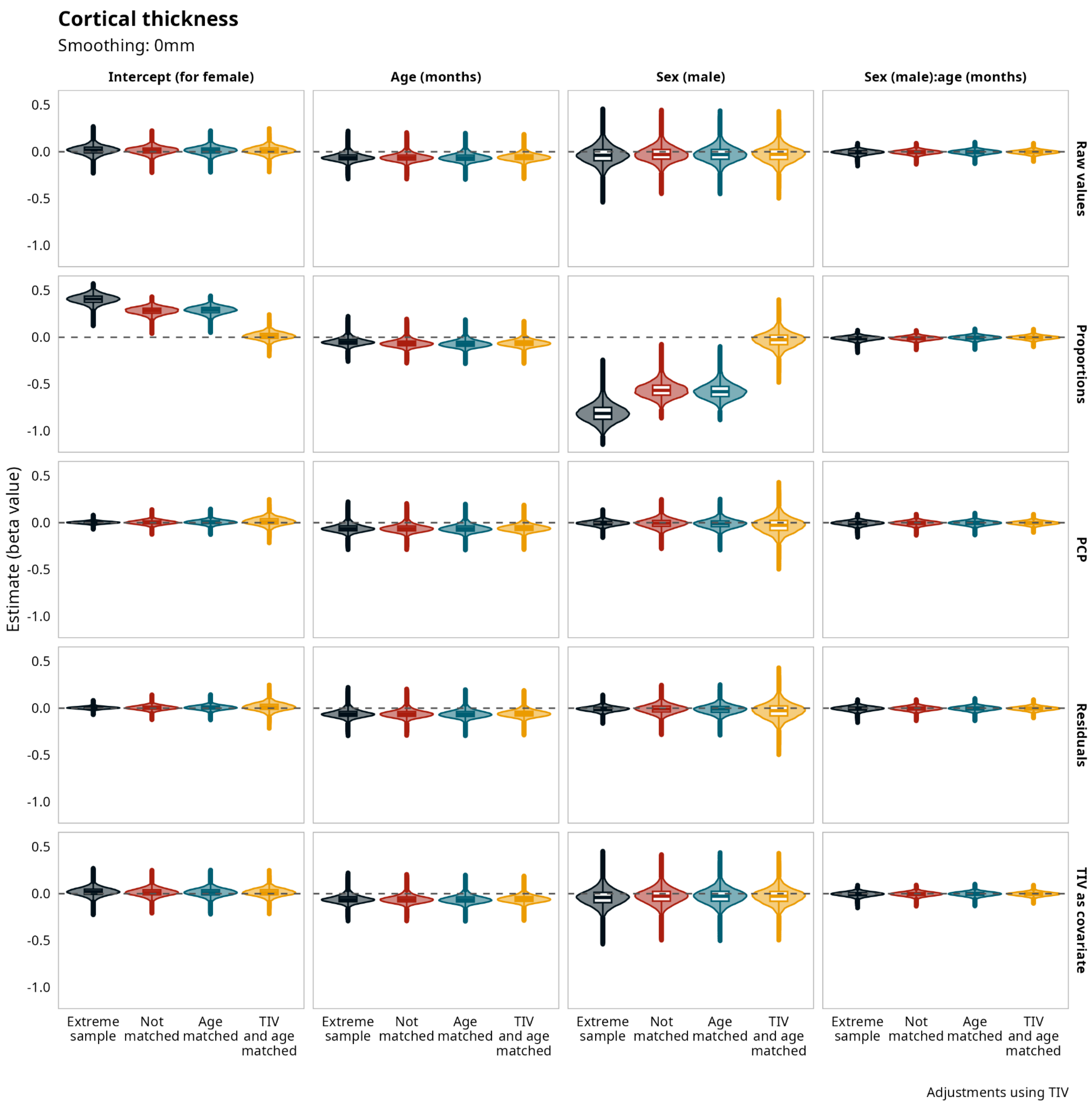

Supplementary Figure 25. Vertexwise Cortical Thickness. Model's estimates for vertexwise cortical thickness without smoothing corrected using total intracranial volume (TIV) for all the samples and all the adjustment methods.

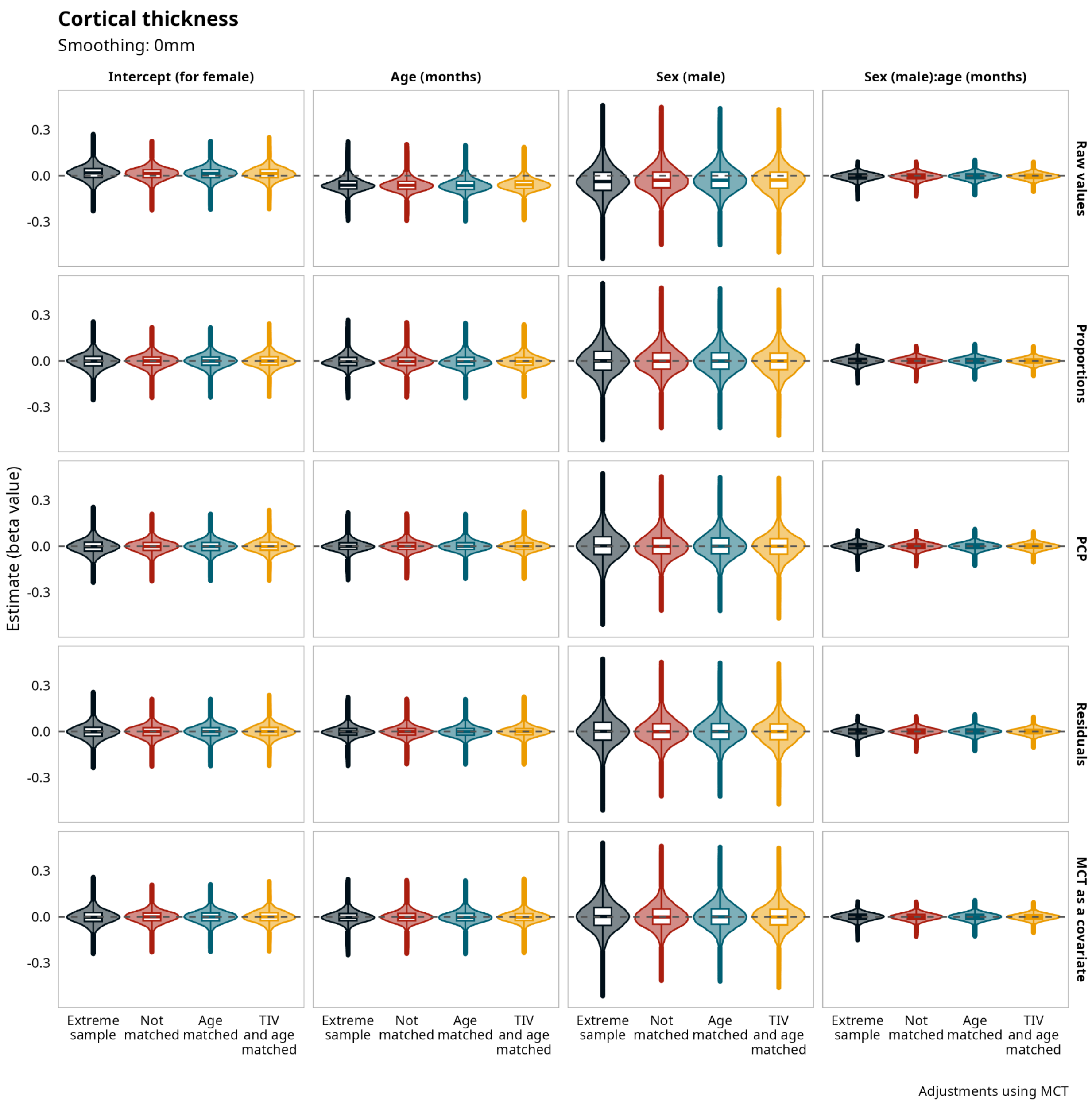

Supplementary Figure 26. Vertexwise Cortical Thickness. Model's estimates for vertexwise cortical thickness without smoothing, corrected using mean cortical thickness (MCT) for all the samples and all the adjustment methods.

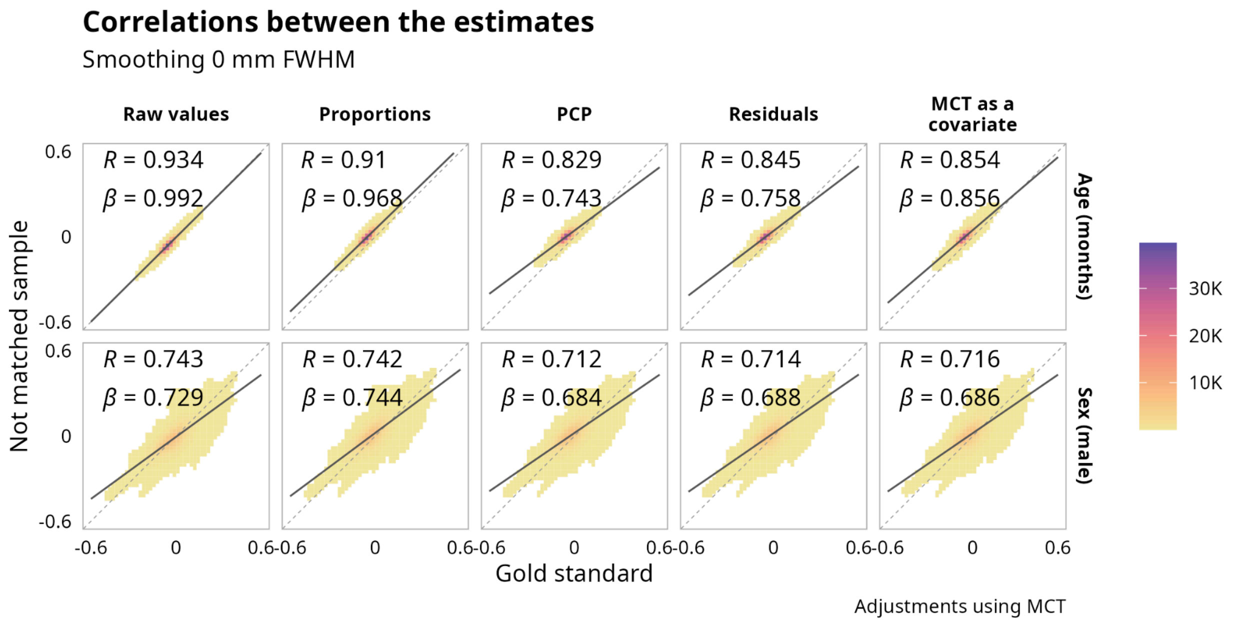

Supplementary Figure 27. Vertexwise Cortical Thickness. Correlations between estimates for the matched sample without adjustment and the estimates after using different adjustment methods with mean cortical thickness (MCT) and the not matched sample, with data without smoothing.

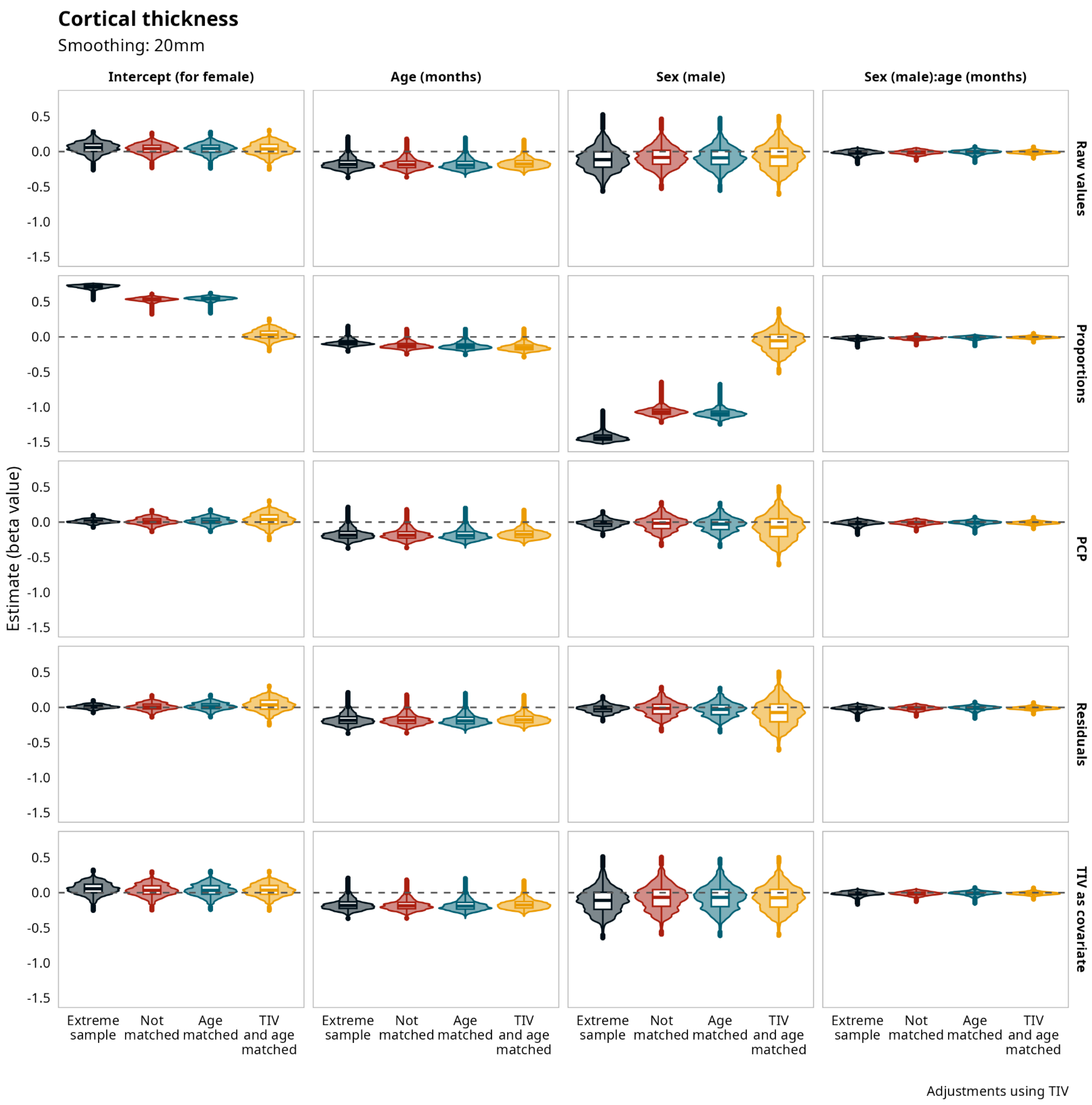

Supplementary Figure 28. Vertexwise Cortical Thickness. Model's estimates for vertexwise cortical thickness smoothed at 20mm FWHM corrected using total intracranial volume (TIV) for all the samples and all the adjustment methods.

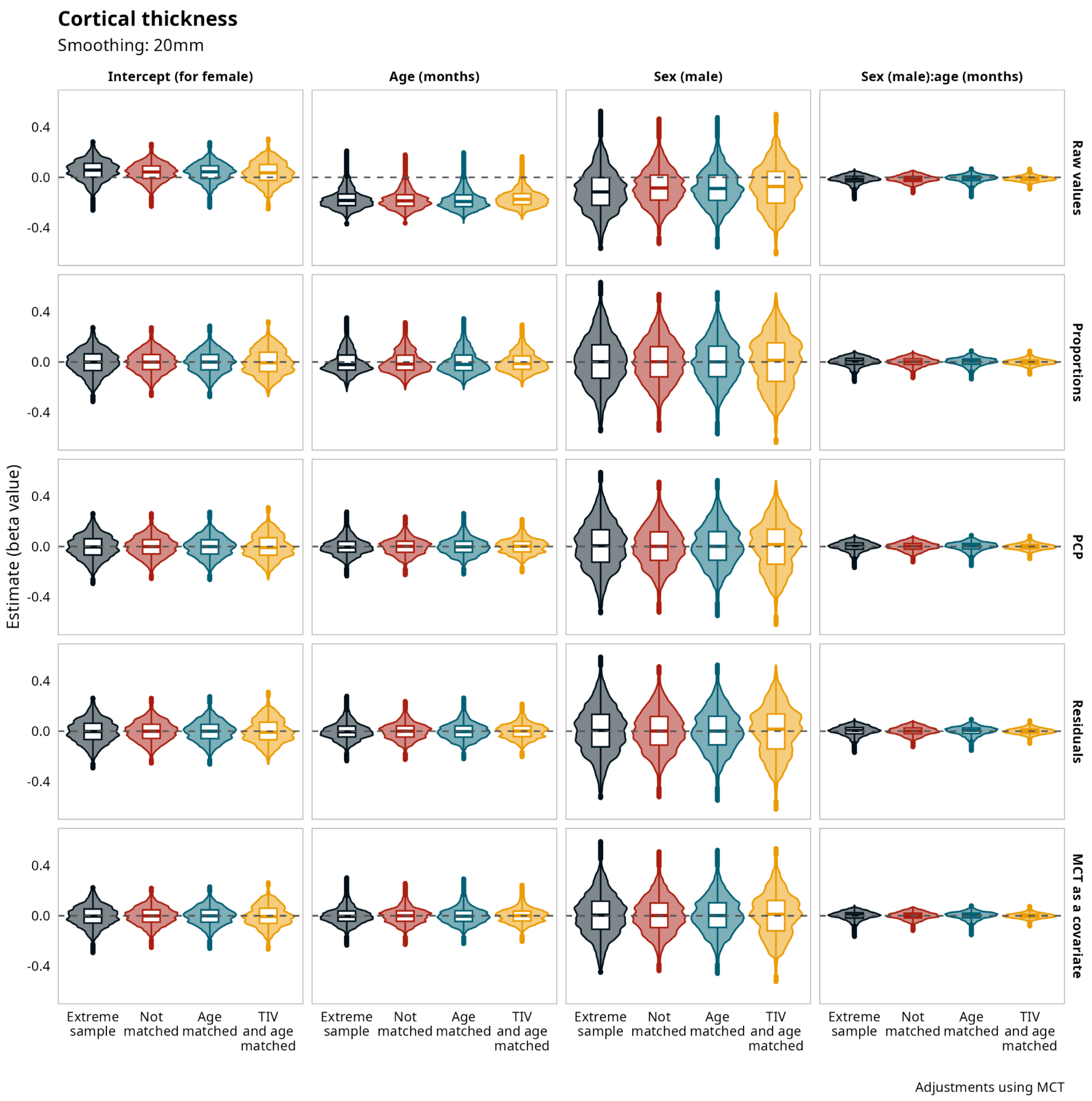

Supplementary Figure 29. Vertexwise Cortical Thickness. Model's estimates for vertexwise cortical thickness smoothed at 20mm FWHM corrected using mean cortical thickness (MCT) for all the samples and all the adjustment methods.

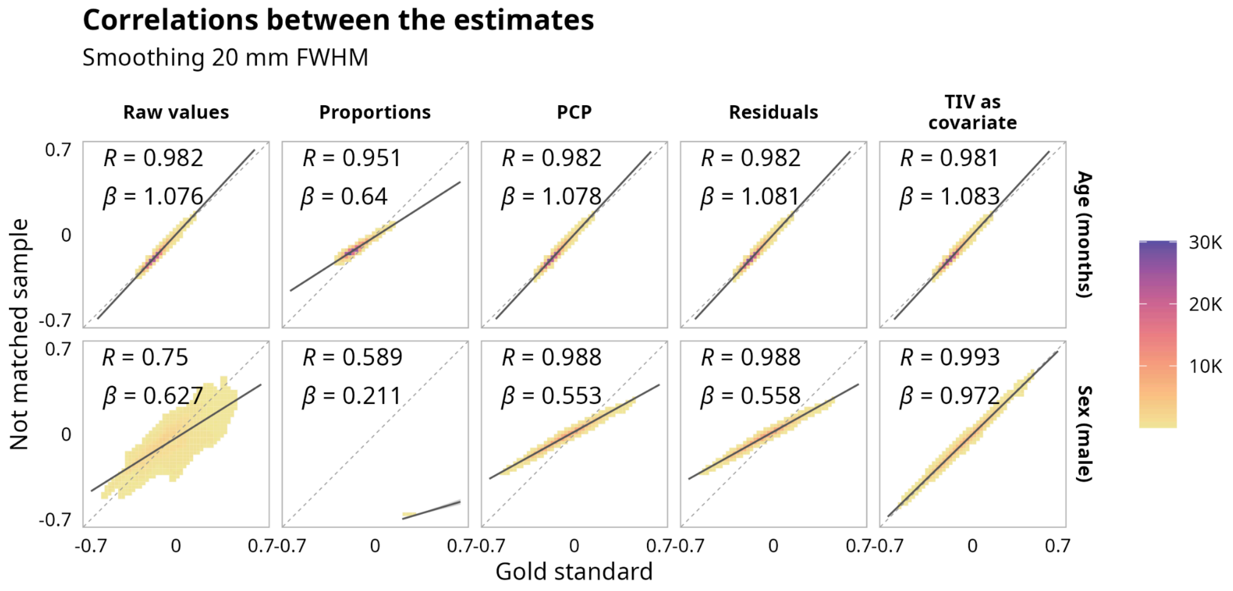

Supplementary Figure 30. Vertexwise Cortical Thickness. Correlations between estimates for the matched sample without adjustment and the estimates after using different adjustment methods with total intracranial volume (TIV) and the not matched sample, with data smoothed 20mm FWHM.

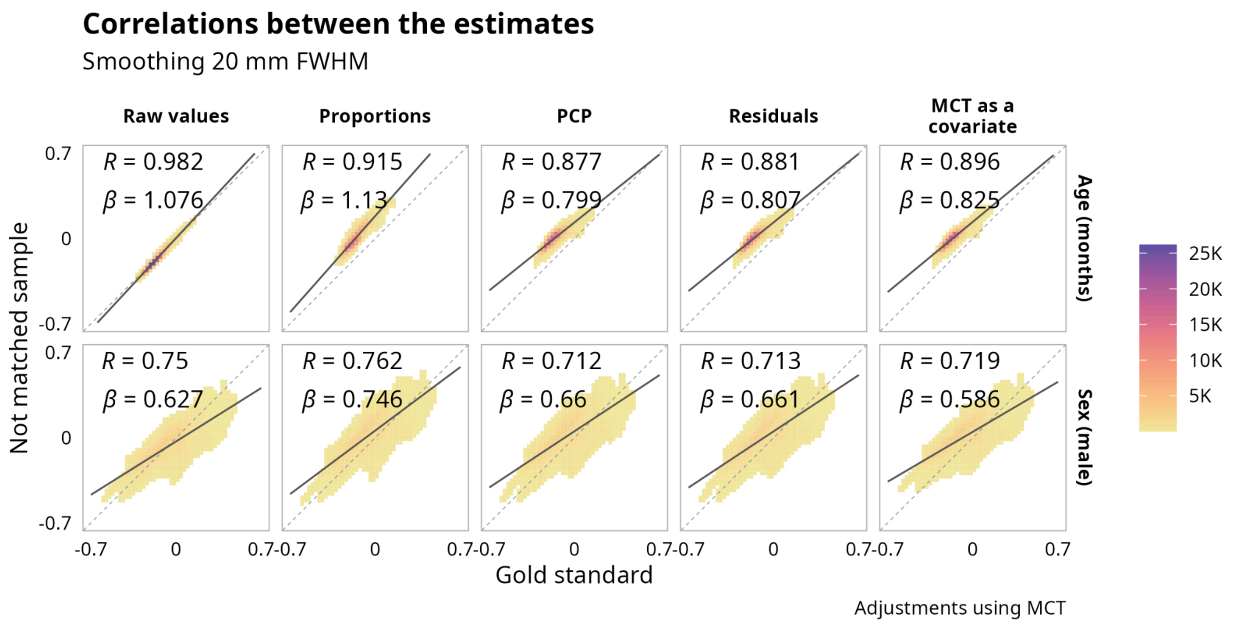

Supplementary Figure 31. Vertexwise Cortical Thickness. Correlations between estimates for the matched sample without adjustment and the estimates after using different adjustment methods with mean cortical thickness (MCT) and the not matched sample, with data smoothed 20mm FWHM.

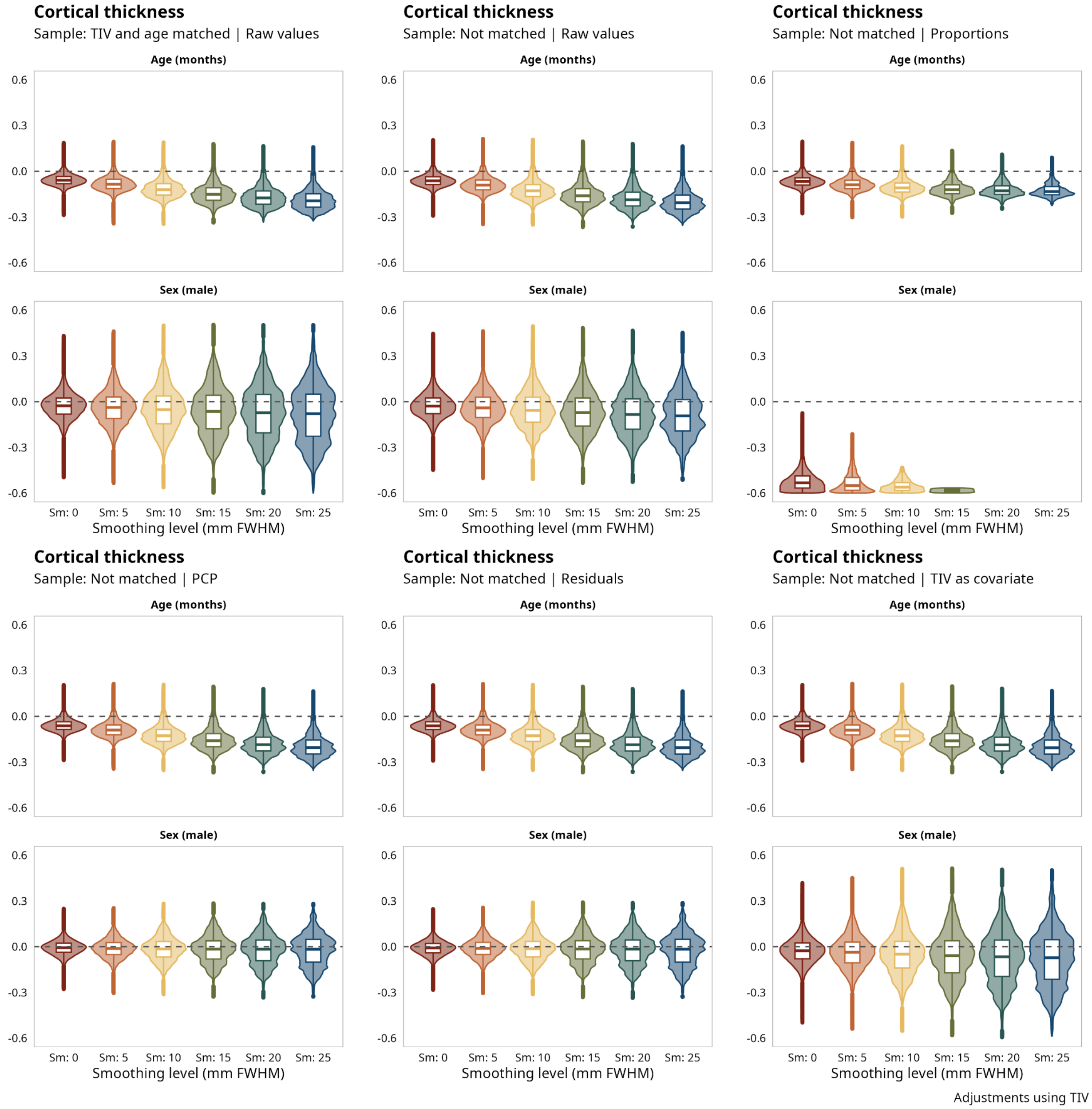

Supplementary Figure 32. Vertexwise Cortical Thickness. Effect of different levels of smoothing on age and sex estimation, using the raw values in the matched and not matched samples and adjusting using total intracranial volume (TIV) in the not matched sample.

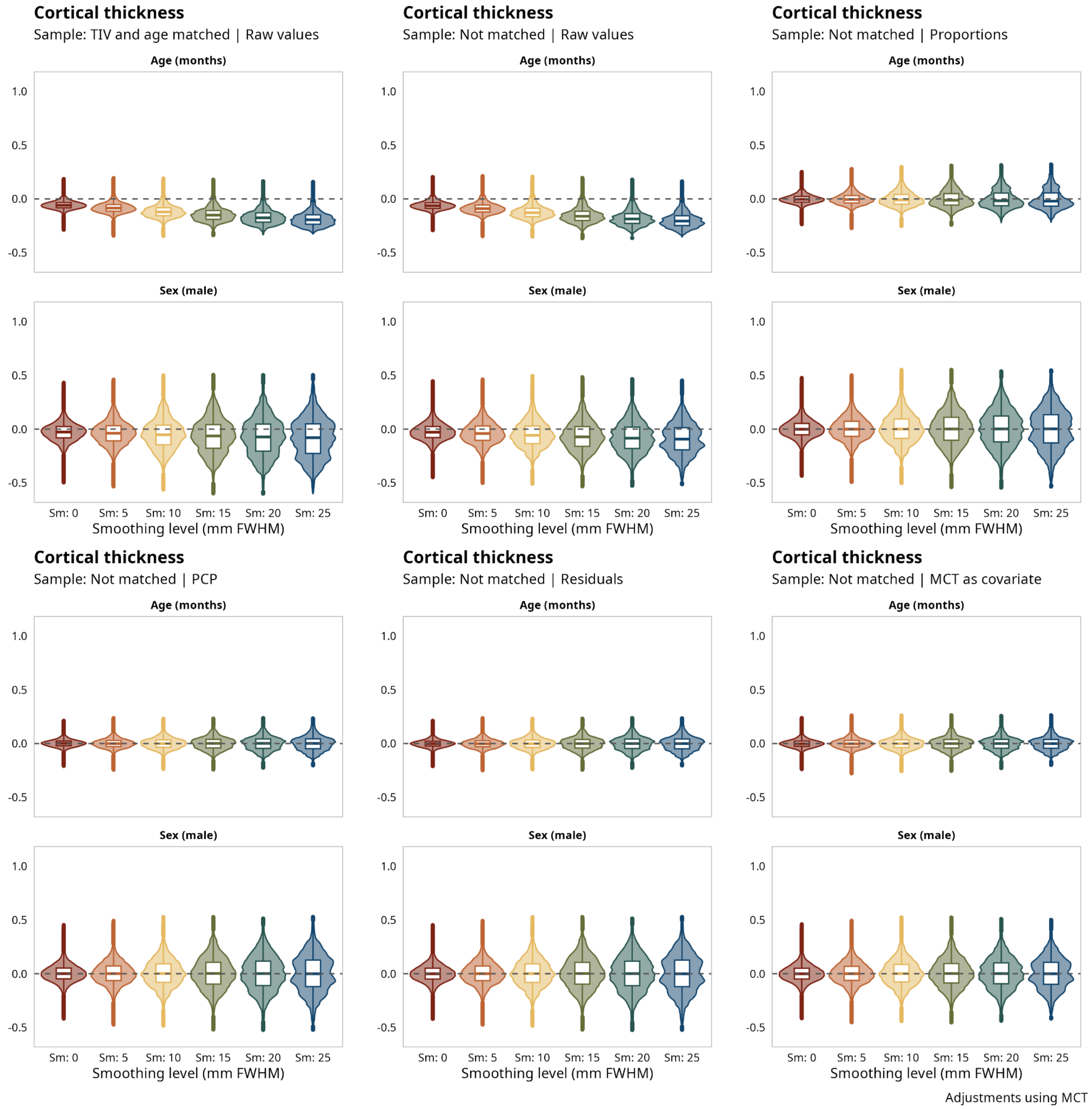

Supplementary Figure 33. Vertexwise Cortical Thickness. Effect of different levels of smoothing on age and sex estimation, using the raw values in the matched and not matched samples and adjusting using mean cortical thickness (MCT) in the not matched sample.

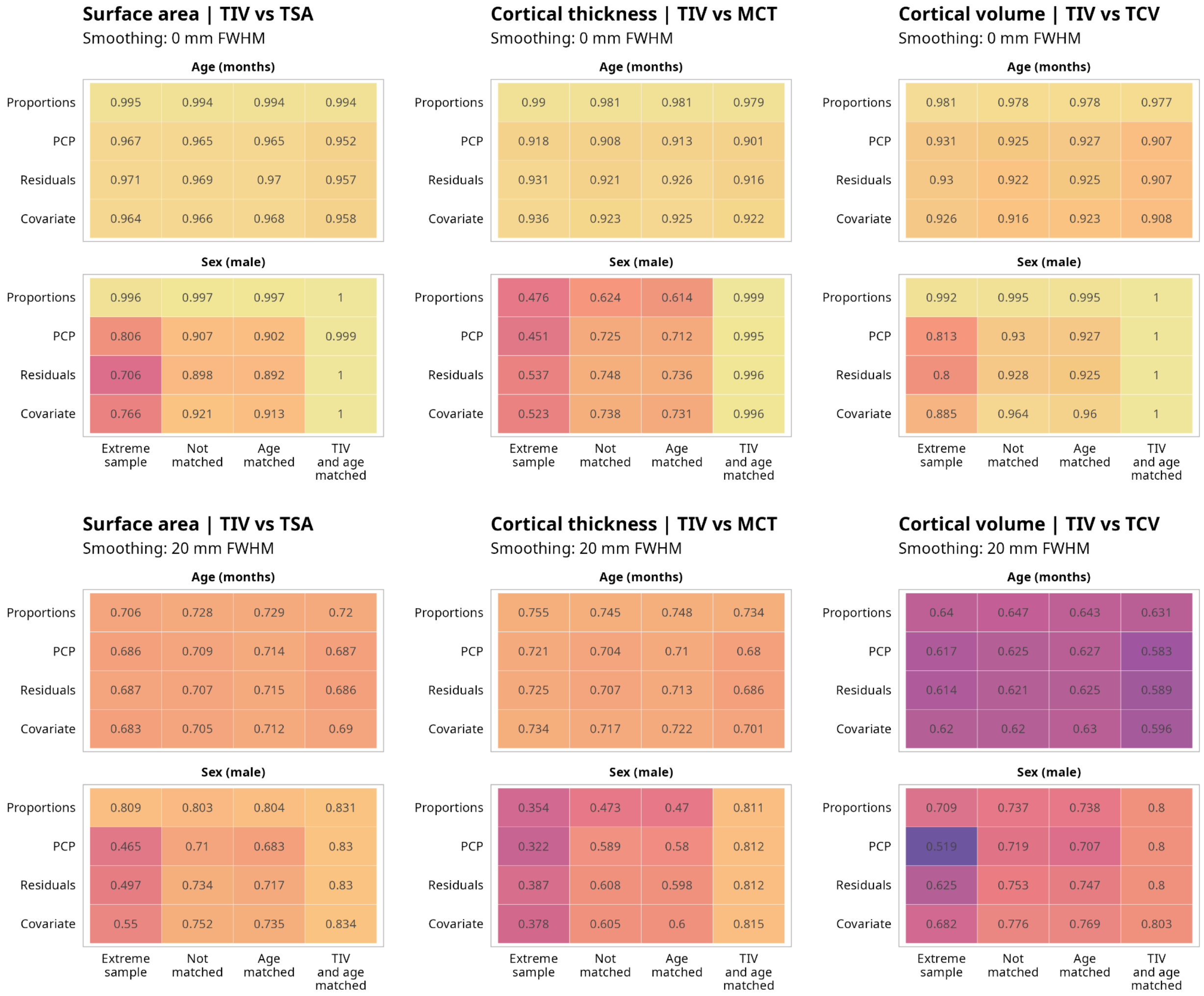

Supplementary Figure 34. Correlations between the estimates using different global measures for adjustment.

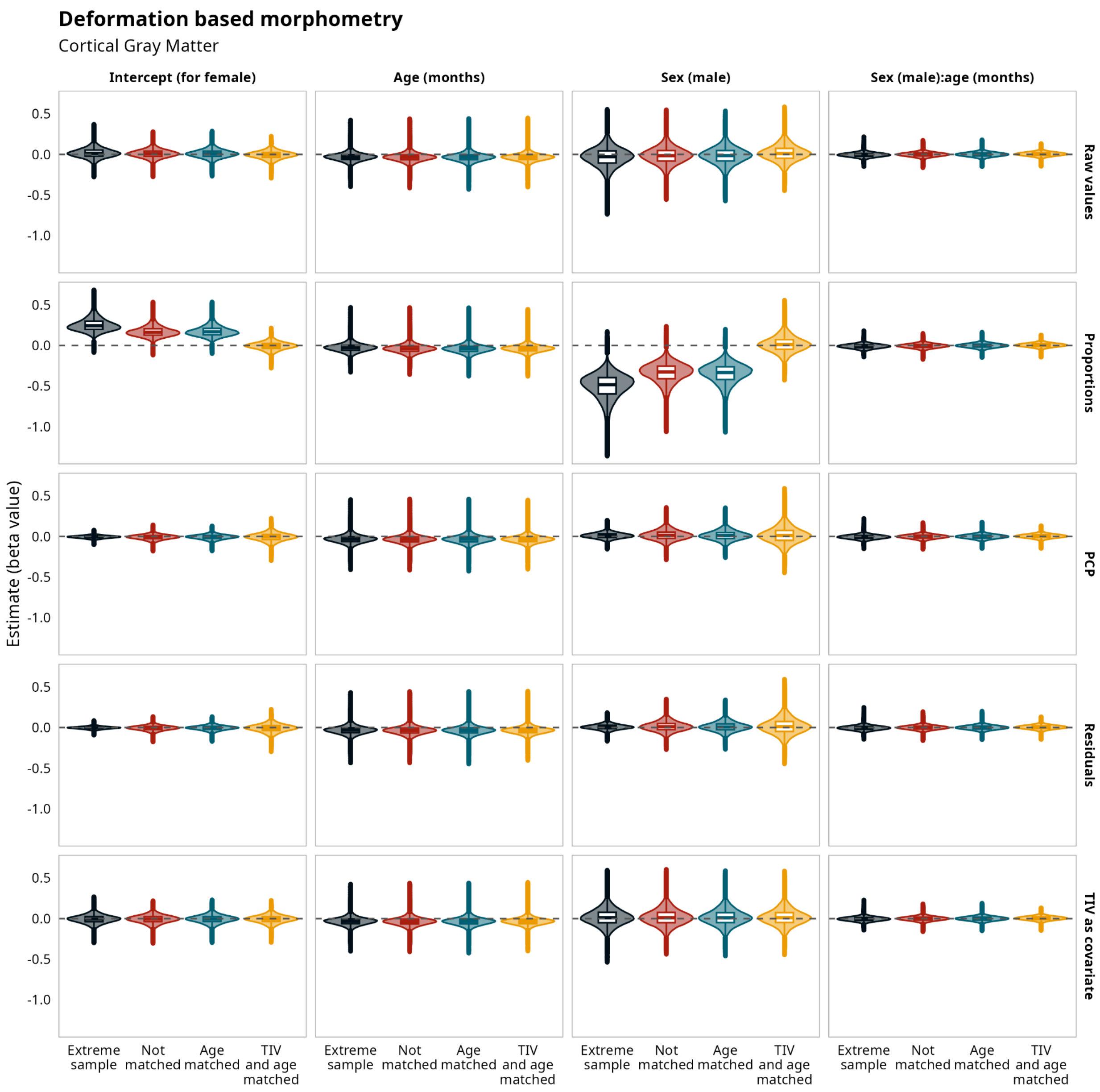

Supplementary Figure 35. Voxelwise deformation based morphometry. Model's estimates for voxelwise deformation based morphometry for the cortical gray matter for all the samples and all the adjustment methods.

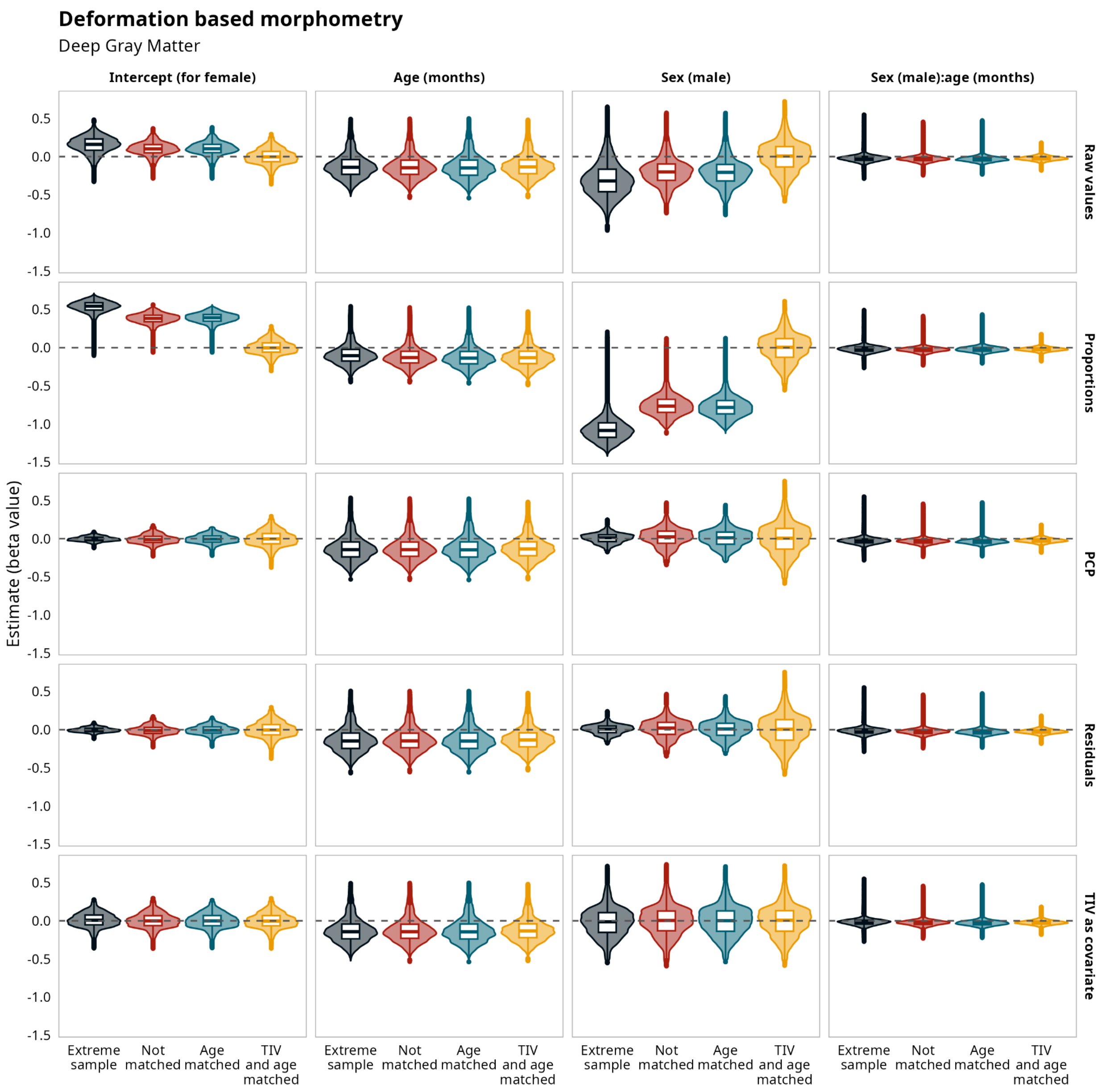

Supplementary Figure 36. Voxelwise deformation based morphometry. Model's estimates for voxelwise deformation based morphometry for the deep gray matter for all the samples and all the adjustment methods.

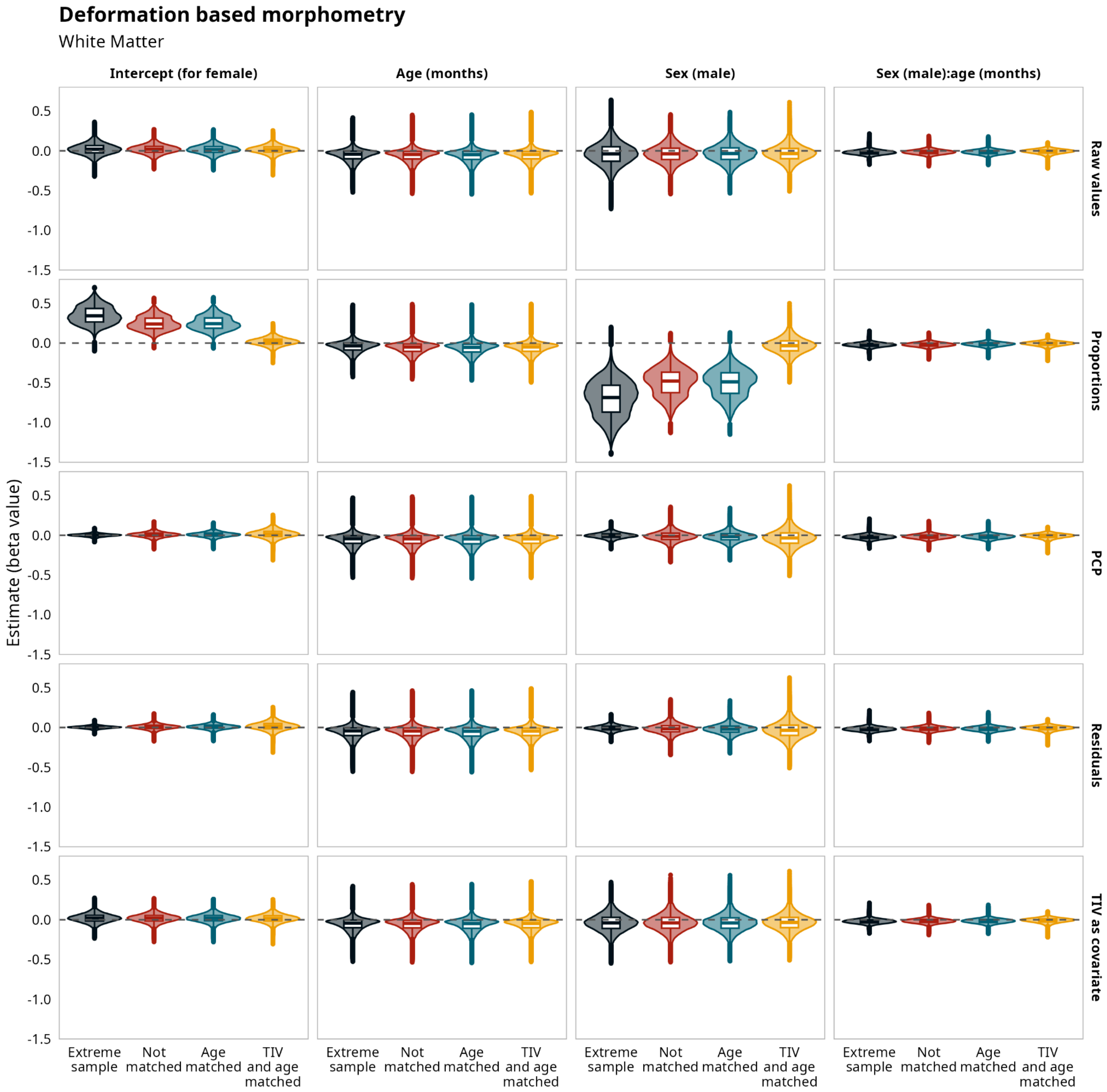

Supplementary Figure 37. Voxelwise deformation based morphometry. Model's estimates for voxelwise deformation based morphometry for the white matter for all the samples and all the adjustment methods.

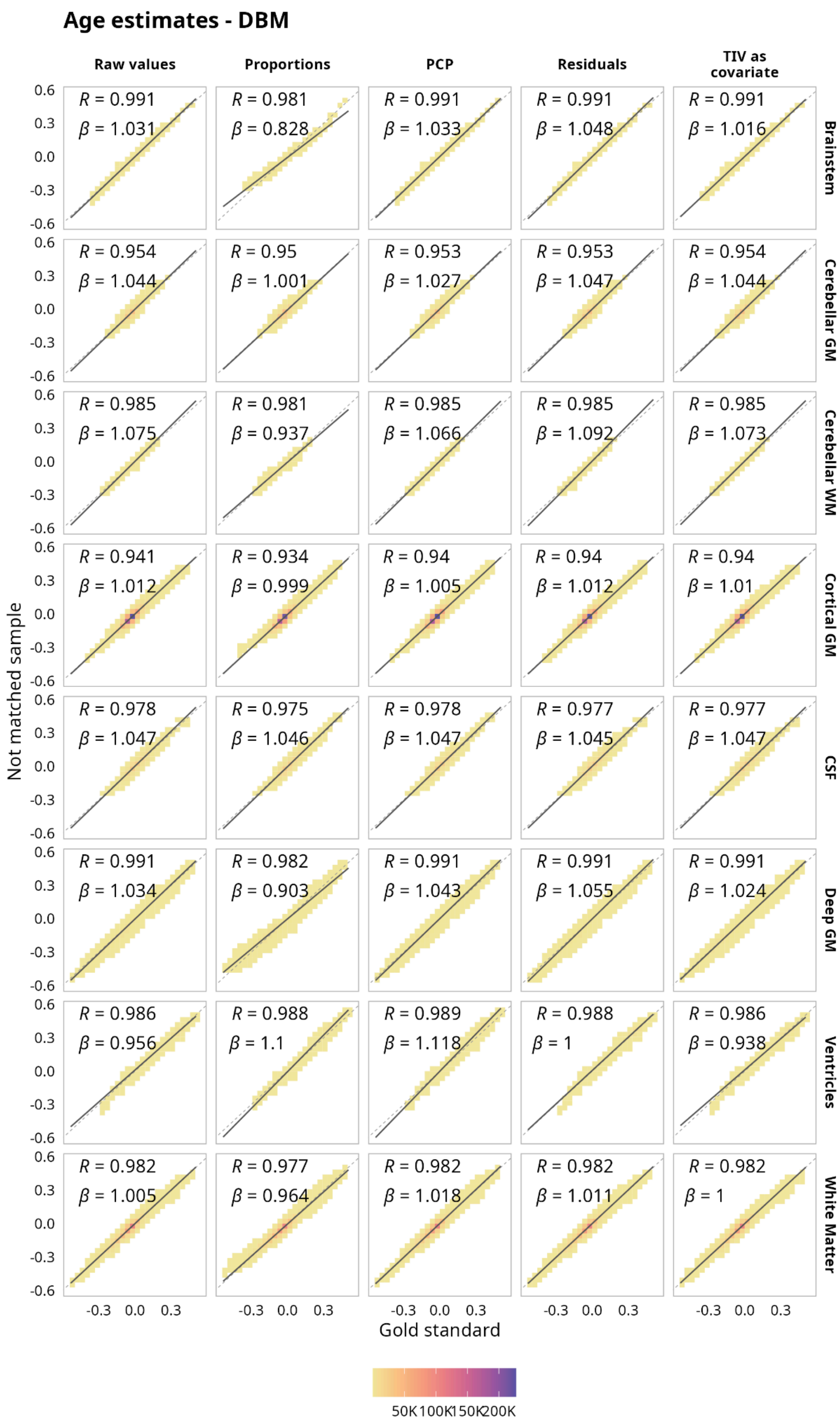

Supplementary Figure 38. Voxelwise Deformation Based Morphometry. Correlations between the age estimates for the matched sample without adjustment and the estimates after using different adjustment methods for the not matched sample on Voxelwise Deformation Based Morphometry across different tissue types.

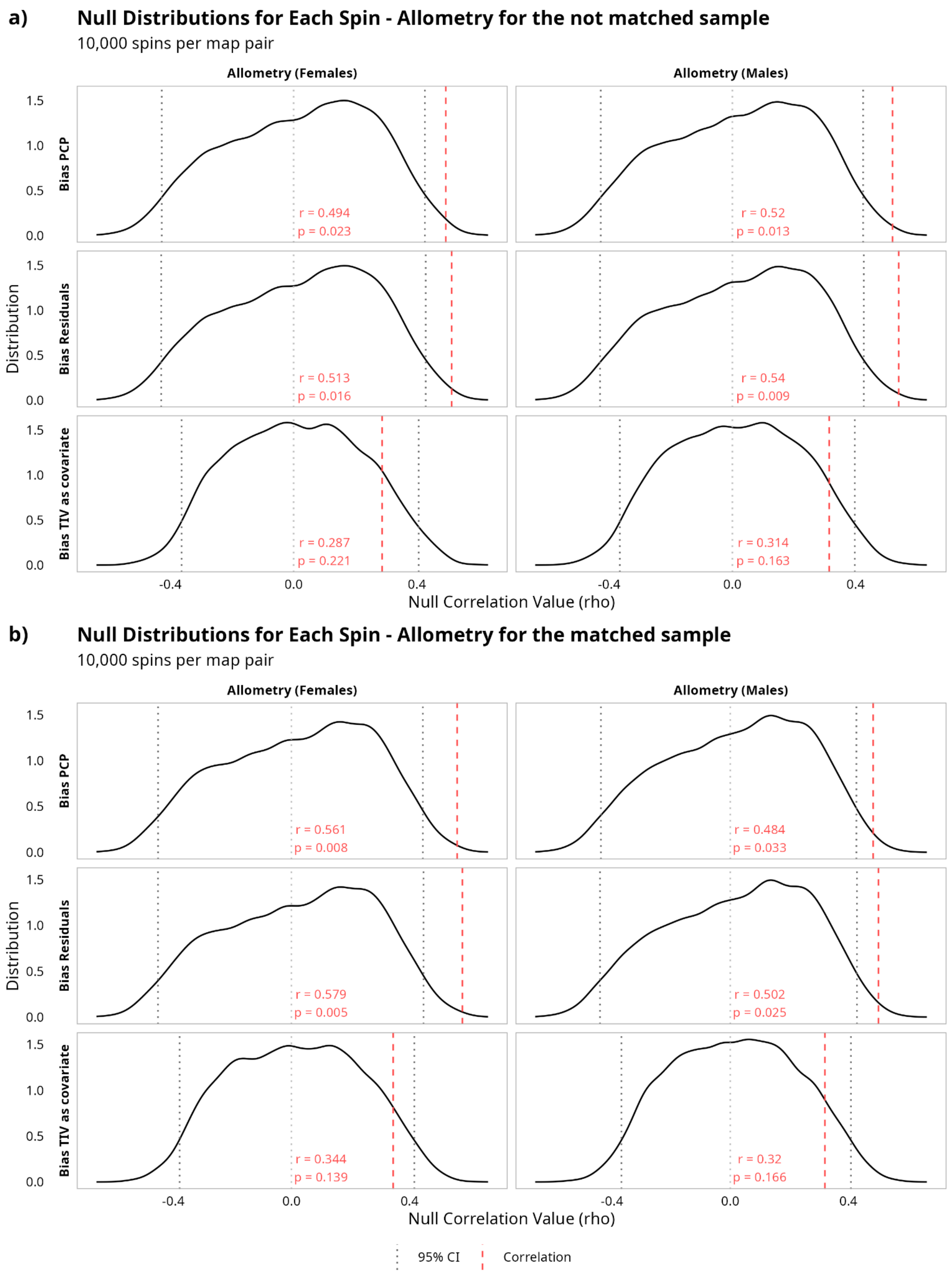

Supplementary Figure 39. Null distribution of the correlation between the bias maps for regional volumes and allometry estimates.

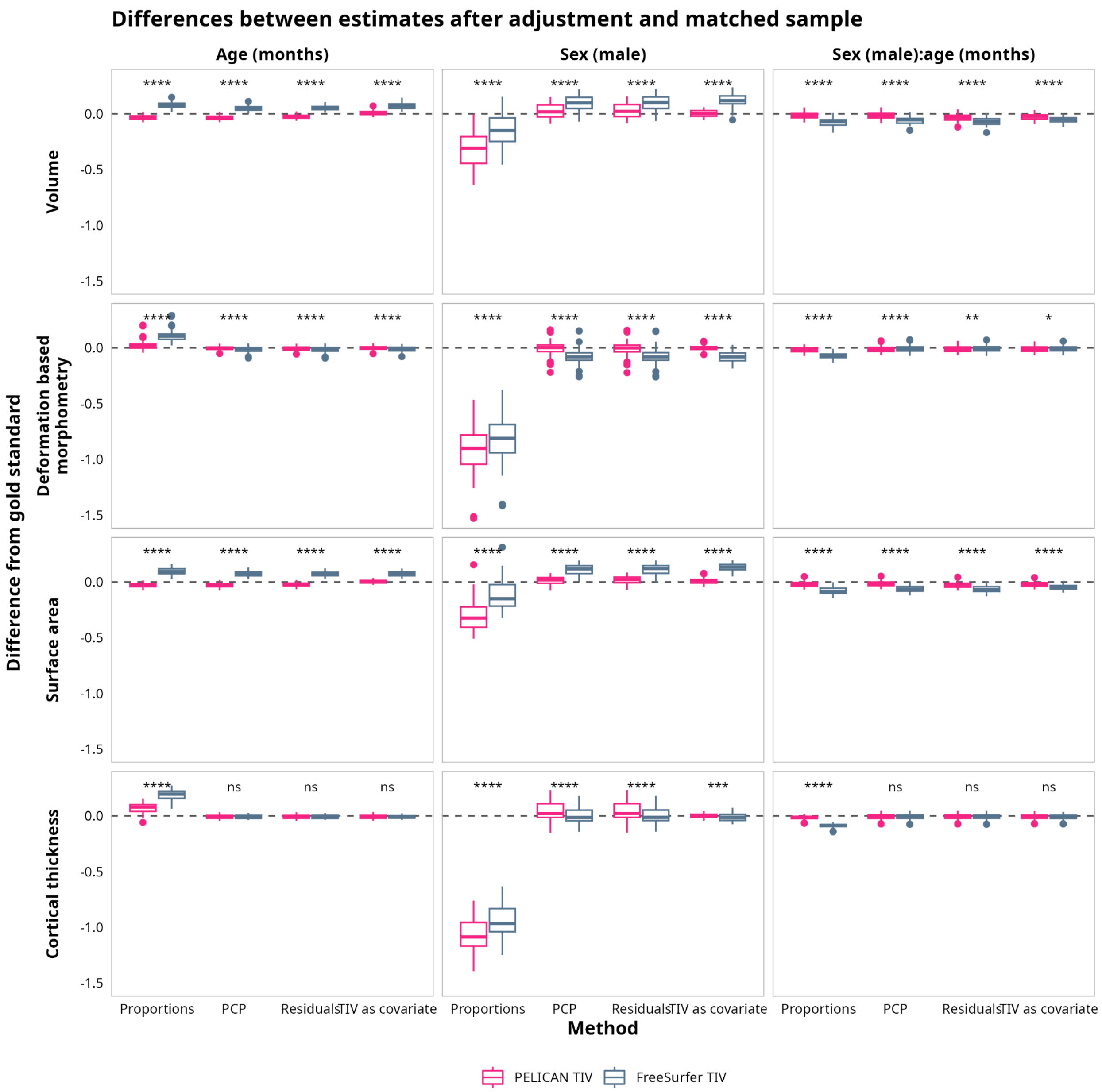

Supplementary Figure 40. PELICAN extracted TIV vs FreeSurfer's estimated TIV. Comparison of the differences between the standardized estimates in the not matched sample after adjusting the region of interest, using PELICAN TIV estimation or FreeSurfer's estimation, and the estimates obtained in the matched sample without any adjustment.

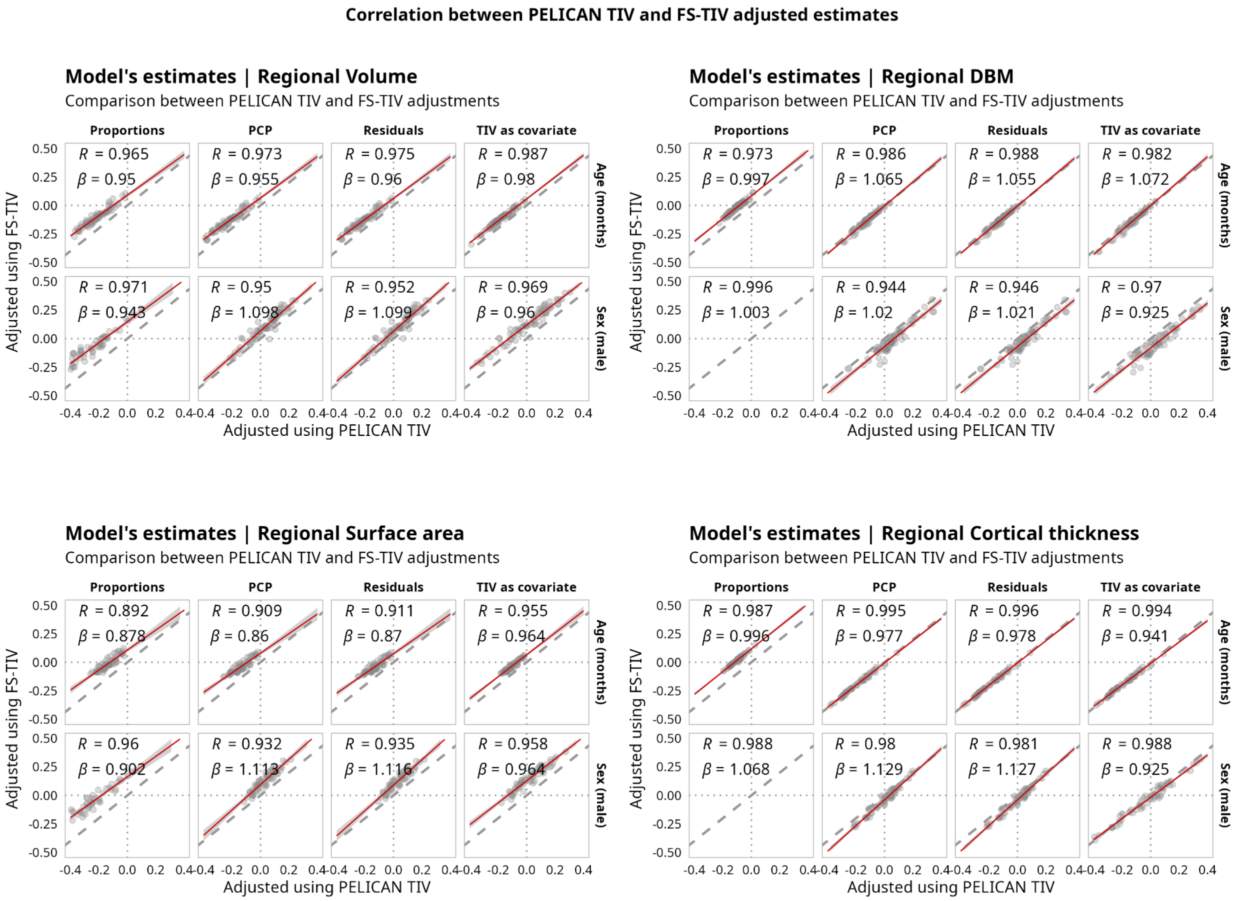

Supplementary Figure 41. PELICAN extracted TIV vs FreeSurfer's estimated TIV. Correlation between the estimates obtained when using PELICAN TIV vs FreeSurfer's TIV in the not matched sample, for each adjustment method and each regional metric. Note that the ranges are restricted.
